# Quantifying the evolution of harmony and novelty in western classical music

**DOI:** 10.1371/journal.pone.0356548

**Published:** 2026-08-20

**Authors:** Alfredo González-Espinoza, Joshua B. Plotkin

**Affiliations:** 1 Department of Biology, University of Pennsylvania, Philadelphia, Pennsylvania, United States of America; 2 University Libraries, Carnegie Mellon University, Pittsburgh, Pennsylvania, United States of America; 3 Meiji Institute for Advanced Study of Mathematical Sciences, Meiji University, Nakano, Tokyo, Japan; Universidad Nacional de Tres de Febrero, ARGENTINA

## Abstract

Music is a complex socio-cultural construct that fascinates researchers across many fields. Understanding its historical development may inform questions of perception and cognition, and offer insight into cultural transmission, creativity, and innovation. Here we study musical features related to harmony and document how they evolved over 400 years in western classical music. We develop a variant of the center of effect algorithm to assign the most likely key to a given set of notes, representing a piece as a sequence of local keys computed bar by bar, and we define information-theoretic measures of key uncertainty, key diversity, and novelty in key transitions. We illustrate these measures with specific examples and give them a musical interpretation, showing how they can be used to study the evolution of harmony. Applying them across the corpus, we confirm several trends previously reported by musicologists and scientists, with some discrepancies during the Classical period: in particular, we find a decline in harmonic innovation during the Early Classical period followed by a steep increase in the Late Classical, for which we offer an explanation consistent with accounts by music theorists. Comparing composers directly by their key-transition vocabularies, we further find that they group by historical period, and that both the overall arrangement and the individual nearest-neighbor relations recover connections consistent with the documented historical record. Finally, we discuss the limitations of this approach for cross-cultural studies and the need for more expressive yet tractable score representations, and a large and reliable corpus, for future study.

## Introduction

Music represents an important part of our lives, whether for listening or playing, as a hobby or profession. From early societies to today, music has been part of everyday life. For this reason, music itself has been a subject of intense study in fields beyond musicology, including cognitive science, sociology, and history. A wide range of scholars have established the value of exploring music through an interdisciplinary lens [1,2].

Studies by musicologists on the evolution of music have provided valuable insight and identified trends in musical style. Although there is recent work on quantitative and statistical analysis of music, most studies have been qualitative. In recent years, the development of technology and digital formats have made access to music data easier for researchers from different fields, allowing them to address questions ranging from social interactions and cultural evolution [3,4] to creativity and innovation [5–7]. However, even with access to digital formats, there remain several challenges unique to music, distinct from those in studies of other recorded cultural traits such as written language, names, or artistic designs. Like the sciences and the other arts, music is a way of representing human experience; the meaning we draw from it is grounded in our perceptual capacities [8,9], and the models we build to describe it are formal abstractions of that experience [10,11], each capturing some aspects of music while setting others aside. One of the key challenges in quantifying musical change is to accurately represent music with quantities that are both mathematically and computationally tractable, yet still musically meaningful even to non-experts.

Three musical elements are often the focus of score analysis: melody, harmony, and rhythm. Although there are many more dimensions to consider, including dynamics and tempo among others, there is a trade-off between the detail and the dimensionality of the musical representation for the purposes of systematic analysis. For example, considering notes from a melody alone would not include the contextual information from the harmony in the chords. On the other hand, considering all notes in a score through formal analysis would include both melody and harmony but increase the alphabet size and dimensionality of the representation. While most studies have focused on melodic representations of musical pieces [12–20], the number of studies considering harmonic properties has increased considerably in recent years [7,21–27].

Harmony and tonality are musical concepts that have been extensively described by musicians, music theorists [11,28–31], psychologists [8,9] and mathematicians [10,32]. Multiple approaches to quantifying harmony have been proposed, such as defining a measure of consonance [33] for chords [25,34] or for individual notes played simultaneously (such as codewords) [7,35]. We focus our study on features related to harmony – specifically the concept of tonality– while seeking to preserve information relevant to the melody and rhythm. We use a mathematical and computational model for tonality based on human cognition, built around musical features of a score that have previously been proposed as tonal indicators, in an effort to preserve as much musical information as possible using features that are both scientifically and musically interpretable. We provide simple examples of our tonal representations for selected pieces and composers, which illustrate the measures we define and quantify in this work, in the hope of making the study accessible to a broad audience.

## Materials and methods

Reducing dimensionality while preserving information from a musical score can be achieved by defining a higher-order feature, beyond the series of individual notes in the score itself. One possibility is to consider chords instead of individual notes, as most music theorists do in harmonic analysis [25,36,37]). This approach requires defining a mapping from sets of notes to chords – e.g., the notes C-E-G map to the C chord. However, developing algorithms to systematically re-label sets of notes as chords across a large corpus is difficult without a clear definition or metric of harmony, and it does not account for note duration and rhythm.

One alternative approach is to define a *local key* given a set of notes in a contiguous region of the score, although this remains an open problem without clear definitions. Several algorithms have been developed to address the problem of local key identification [38–40]. We implement a variation of the *center of effect* algorithm introduced by Chew [41], which has been used for music information retrieval tasks [42–44] and has been shown to outperform other algorithms when evaluated against gold-standard hand annotations by musicologists [45].

### Key representation in the spiral array

**Fig 1:**
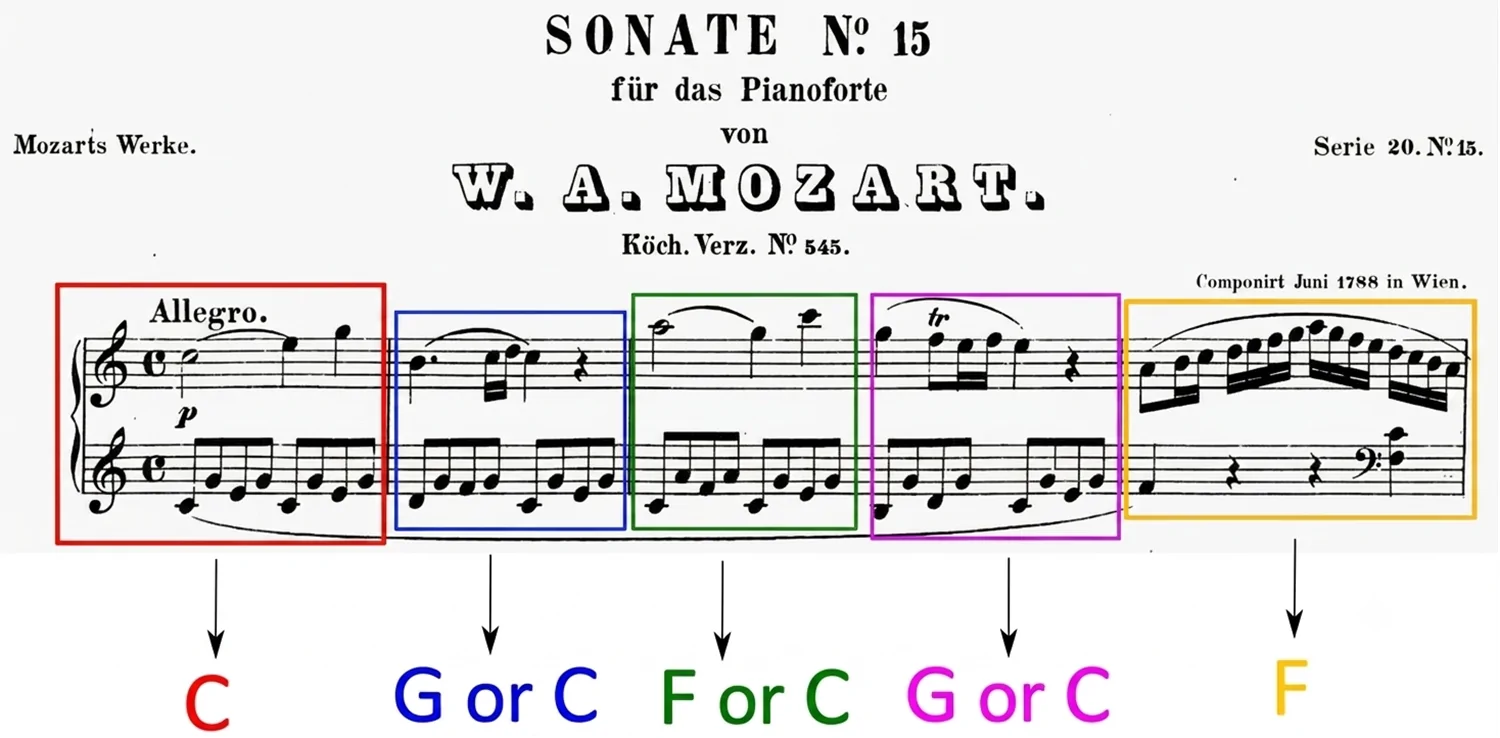
Example of determining the “local key” of each bar. The figure shows the first five bars of the piano sonata K. 545 in C by W. A. Mozart, along with the most likely key (or two most likely keys) as determined by the CoE algorithm.

We represent a musical score as an ordered sequence of elements: $\xi =(e_{1},e_{2},...,e_{n})$, where each element corresponds to the local key of a given bar (or a fixed number of bars) in the piece (see Fig 1). We use a geometric representation in which notes, chords, and keys are represented as points $(x,y,z)\in {\mathbb{R}}^{3}$ in a helix-type configuration known as the spiral array [45]. Throughout we assume both octave equivalence and enharmonic equivalence, so that pitches an octave apart, and pitches with different names but the same pitch class (such as C♯ and D♭), are treated as identical. The enharmonic assumption is required by our 12-note MIDI encoding rather than adopted on theoretical grounds; without it the representation would form a torus instead of a helix. We return to this modification of the original spiral array below and in S2 Appendix.

Using the spiral representation and the center of effect as a model for tonality allows us not only to analyze a large number of musical scores but also to relate their mathematical features to concepts from information theory. Derived from the Tonnetz network in Riemann’s theory [46–48], the spiral representation preserves the hierarchical structure of tonality: key representations are generated from combining chords, and chord representations from combining notes, while representing all these structures in the same space (${\mathbb{R}}^{3}$). Because notes ordered by fifths, the chords built from them, and the keys built from those chords all inhabit this same space, Euclidean proximity in the spiral array is harmonic proximity: tonally close keys—such as a tonic and its dominant—map to nearby points, while harmonically remote keys map far apart. This geometric fidelity, inherited from the Tonnetz, is what makes a distance-based measure of tonal ambiguity musically meaningful, and it underlies the key-uncertainty measure defined below.

Notes in the spiral array are defined as


$$\begin{array}{c} \vec{p}(k)=[\begin{array}{c} x_{k}\\ y_{k}\\ z_{k} \end{array}]=[\begin{array}{c} r\sin\frac{k\pi }{2}\\ r\cos\frac{k\pi }{2}\\ kh \end{array}], \end{array}$$ (1)

where *r* and *h* are parameters (see S1 Appendix) and *k* is an integer representing a specific note. The starting note *k*_0_ is arbitrary; for simplicity, we define *k*_0_ as the C note. Notes *k* and *k* + *n* are separated by *n* fifths, preserving the harmonic relationships among notes, chords, and keys (e.g., with *k*_0_ = C, *k* + 1 = G, *k* + 2 = D and so on). Major and minor chords (${\vec{C}}_{M}$ and ${\vec{C}}_{m}$) are constructed as linear combinations of notes and major and minor keys (${\vec{T}}_{M}$ and ${\vec{T}}_{m}$) as linear combinations of chords (see S1 Appendix).

The *center of effect* is based on the analogy that a set of notes has the equivalent of a center of mass. A set of notes $𝒩=\{{\vec{p}}_{1},{\vec{p}}_{2},...,{\vec{p}}_{N}\}$ has an effective center of mass in the form of a linear combination of its elements


$$\begin{array}{c} {\vec{c}}_{e}=\sum_{i=1}^{N}\omega_{i}\vec{p_{i}}. \end{array}$$ (2)

Here, the coefficients $\omega_{i}$ are normalized ($\sum \omega_{i}=1$) and they represent the *importance* of each note. These coefficients can be defined in multiple ways (see S2 Appendix) but we choose to use the normalized duration of each note – so that our representation captures some aspects of rhythmic structure. The center of effect (CoE) key-finding algorithm we develop uses the vector ${\vec{c}}_{e}$ for a given set of notes, and defines the most likely key as:


$$\begin{array}{c} arg\;min_{T\in T}||{\vec{c}}_{e}-\vec{T}||, \end{array}$$ (3)

which corresponds to the key *T* for which the Euclidean distance to the center of effect of that set of notes is minimized. Here T is the set of all possible major and minor keys: $T=\{T_{M}(k)\forall k\}\cup \{T_{m}(k)\forall k\}$. The CoE method also assigns a likelihood to the most likely local key, based on the Euclidean distance above, as well as a likelihood to the nearest alternative keys based on their respective distances (Eq (6)).

The CoE algorithm has proven effective and to perform better than other methods when identifying a key from a small amount of information [45], and has proven useful not only for identifying a key but for other applications such as spelling [42] and passage segmentation, even with post-tonal music by composers such as Messiaen [43]. By construction, the spiral representation is enharmonically inequivalent, meaning that it distinguishes between sharp and flat notes that equal temperament would consider to be the same (e.g., C# and Db). This can, in most cases, be an advantage of the model. However, for our purpose of using a high-order representation, we modified the CoE algorithm to make it compatible with a 12-note representation and with enharmonic equivalence — the convention that two notes with different names but the same pitch are treated as identical, such as C♯ and D♭, which sound the same on a piano even though they are written differently. The distinction becomes relevant in microtonal and other non-equal-tempered tunings, where the two can take slightly different pitches; under the equal temperament assumed here they coincide, so we treat them as the same (see S2 Appendix).

We evaluated the accuracy of our implementation of the CoE (center of effect), including the various weights we introduce in this context, by comparing its output to a gold standard of local keys annotated by hand, bar by bar, for Beethoven’s string quartets [37] and Mozart’s piano sonatas [49]. These annotated datasets are some of the most detailed sets of such information in the literature; it contains information about the key and chord in functional harmony for each bar. Our CoE implementation matches the hand annotations for chords in more than 60% of the bars in the corpus on average, and mismatches are often musically plausible alternatives (see S3 Appendix). For local keys, we obtained more than 80% accuracy for the Mozart piano sonatas, and more than 55% of accuracy for the Beethoven’s string quartets. Additionally, we compared our implementation of the CoE algorithm with the Krumhansl-Schmuckler (KS) key-finding algorithm, finding approximately a 10 percentage point difference in accuracy in favor of our CoE implementation.

### Key diversity and uncertainty

Leonard B. Meyer’s information-theoretic account of music holds that musical style is inherently probabilistic, and that listening is an ongoing process of expectation and resolution under uncertainty [50]. Building on this view, we describe the harmonic content of a piece with two information-theoretic quantities defined on its sequence of local keys: *key diversity* and *key uncertainty*. Both are Shannon entropies of a probability distribution over keys, and differ only in which distribution and at what scale. We use Shannon’s entropy, the expected information content of a random variable *X*, $H(X)=-\sum_{x\in X}p(x)\log p(x)$, which is low when probability concentrates on a single outcome and high when it spreads evenly across many. Key diversity applies this functional *across* the bars of a piece, key uncertainty applies it *within* a single bar.

#### Key diversity.

Key diversity captures how varied the tonal centers of a piece are: whether it stays on a few keys or ranges widely across many. For each piece $\xi =(e_{1},...,e_{n})$ of assigned per-bar keys, we build the distribution of keys by their normalized frequency,


$$\begin{array}{c} p(e_{i})=\frac{f(e_{i})}{\sum_{e_{j}\in \xi }f(e_{j})}, \end{array}$$ (4)

where $f(e_{i})$ counts the bars assigned key $e_{i}$ and the sum runs over the distinct keys appearing in ξ. Key diversity is the entropy of this distribution,


$$\begin{array}{c} \text{Key Diversity}=-\sum_{e\;\in \;\xi }p(e)\;\log p(e), \end{array}$$ (5)

this feature is low when the piece remains in a few keys and high when many keys occur with comparable frequency.

#### Key uncertainty.

Key uncertainty quantifies how *ambiguous* the tonal reference of a bar is, whether its notes point clearly to one key or could belong to more than one. Because distance in the spiral array corresponds to harmonic proximity, this ambiguity is already encoded in the geometry. A bar’s center of effect ${\vec{c}}_{e}$ is a single point, and its placement relative to the surrounding keys provides the following insight: when ${\vec{c}}_{e}$ sits close to one key, the tonal reference is clear, when the center is roughly equidistant from several keys, none of them is favored and the reference is ambiguous. This implies that the probability of ${\vec{c}}_{e}$ to be in a key should be a function to its distance to that key.

To turn this geometric feature into a number, we first need to map the distances from ${\vec{c}}_{e}$ to the candidate keys onto a probability distribution over those keys. The center of effect algorithm returns the keys ordered by distance, with $d(T_{\left(r\right)})=\|{\vec{c}}_{e}-T_{\left(r\right)}\|$ the distance to the *r*-th nearest key. A natural choice is the Boltzmann (softmax) map, in which probability decays smoothly with distance:


$$\begin{array}{c} p(T_{\left(r\right)})=\frac{e^{-\lambda d(T_{\left(r\right)})}}{\sum_{s=1}^{N}e^{-\lambda d(T_{\left(s\right)})}}, \end{array}$$ (6)

evaluated over the *N* = 12 keys nearest to ${\vec{c}}_{e}$, ordered $d_{\left(1\right)}\leq \cdots \leq d_{\left(N\right)}$. The form is borrowed from statistical mechanics, where the distances act as energies and λ as an inverse temperature, so a key receives high probability when it is harmonically close to ${\vec{c}}_{e}$ and low probability when it is remote—just as a thermal system is found most often in its low-energy states. Among all distributions consistent with the distance vector, this is the least-committal (maximum-entropy) choice: it introduces no assumption beyond “harmonically closer is more probable.” The spiral array’s distances are in arbitrary units, so a scale factor is needed to turn them into probabilities, and that factor is λ. Rather than tune it to the data, we fix it from the geometry alone, through a single requirement: a bar whose center of effect lands exactly on a key should be assigned that key with near-certainty (*p*_0_ > 0.98). The distances to the competing keys are then nothing other than the fixed key-to-key spacings of the spiral array. This requirement therefore leaves λ as the only unknown and pins it to a unique value, λ≈12 (S9 Appendix).

Key uncertainty is the entropy of this per-bar distribution, computed once per bar, and the quantity presented is the median per-piece value over the *M* bars of the piece:


$$\begin{array}{c} \text{Key Uncertainty}=\underset{m\in \{1,...,M\}}{\text{median}}\left(-\sum_{r=1}^{N}p_{m}(T_{\left(r\right)})\log p_{m}(T_{\left(r\right)})\right), \end{array}$$ (7)

where *m* is the bar index and the inner sum runs over the *N* = 12 nearest keys of Eq (6). The interpretation follows: a bar whose distribution concentrates on one key has entropy near zero (an unambiguous tonal reference), whereas one spread over several comparably close keys has high entropy (an ambiguous reference). This is tonal ambiguity in the per-bar tonal reference, and not structural or systemic complexity in the sense studied in the complex-systems literature [51,52]. Restricting the sum to the local neighborhood rather than all 24 keys leaves the per-bar values essentially unchanged, because remote keys carry negligible probability mass; we confirm this cutoff invariance, and the robustness of the reported trends to λ, in S9 Appendix. Summarizing a whole piece by the median of its per-bar values is deliberately reductive: the distribution of per-bar uncertainties is typically broad and often multimodal, so a single scalar cannot capture how tonal ambiguity rises and falls within a piece. We use the median here for comparability across the corpus, but a fuller treatment would follow the per-bar uncertainty profile at higher temporal resolution, and we regard the development of such a dynamic representation as an important direction for future work.

### Innovation in key transitions

Following the same insights from Meyer, in his analysis of musical experience: “... the importance of uncertainty in musical communication, the probabilistic nature of musical style, and the operation in musical experience of what I have since learned to be the Markoff process” [53], we use Markov models to represent musical pieces. Although notes in a musical score are known to exhibit long-range dependencies [23,54], several studies have used first-order Markov chains to model musical scores [7,25,55], where the state $s_{t+1}$ in a musical sequence depends only on the previous state $s_{t}$ (or a set of previous states $\left\{s_{t},s_{t-1}...\right\}$ in higher-order models). Markov chains on higher-order representations, such as *local keys* viewed as harmonic transitions, are likely more useful than Markov Chains on individual notes, which lack contextual information [20]. We implemented a Bayesian Information Criterion (ΔBIC) test and a more adequate permutation test for this type of system that supports our Markov-1 order assumption (see S7 Appendix).

A piece $\xi =(e_{1},e_{2},...,e_{n})$ can be seen as a Markov chain, itself the outcome of a Markov process, whose states are the elements (local keys), drawn from an alphabet 𝒜 of |𝒜|=24 keys (12 major + 12 minor). Indexing the keys by i∈{1,...,|𝒜|}, a transition is an ordered pair i→j, and the chain is described by a transition matrix *P* with entries p(j∣i), the probability of i→j (initial state *e*_1_). Given an empirical score represented as a series of local keys, *P* is estimated by maximum likelihood:


$$\begin{array}{c} p(j|i)=\frac{n_{ij}}{n_{i}},\;n_{i}=\sum_{j}n_{ij}, \end{array}$$ (8)

where $n_{ij}$ is the count of the transition i→j in the piece and $n_{i}$ the number of times key *i* is followed by any key, equal to the frequency of *i* as a context.

One of our goals is to evaluate how *innovative* a given piece ξ is when compared with a set of prior works. This comparison is made by assuming that a model *Q* representing all prior works can be used to generate ξ. We quantify how well *Q* reproduces *P*. This quantity is directly related to the Kullback-Leibler divergence, which measures the additional information required to encode a distribution *P* using a reference distribution *Q*. For stochastic matrices the KL-divergence rate is defined as (see S5 Appendix):


$$\begin{array}{c} D_{KL_{R}}(P\|Q_{\beta })=\sum_{i}\mu_{i}\sum_{j}p(j|i)\;\log\left(\frac{p(j|i)}{q_{\beta }(j|i)}\right), \end{array}$$ (9)

where $\mu_{i}$ is the stationary distribution of elements in ξ and $q_{\beta }(j|i)$ are the entries of the smoothed historical transition matrix $Q_{\beta }$ defined below. Here, $D_{KL_{R}}(P\|Q_{\beta })$ quantifies the information per step required for the model $Q_{\beta }$ to reproduce *P*.

To quantify the innovation of a piece composed in year *t*, we aggregate all key transitions from pieces composed in years earlier than *t* into a cumulative historical pool $Q_{\beta }^{<t}$, with counts $m_{ij}$ and $m_{i}=\sum_{j}m_{ij}$. Because an empirical pool assigns zero probability to any transition it has never seen, $Q_{\beta }^{<t}$ is regularized by additive smoothing (Dirichlet) with a single parameter β:


$$\begin{array}{c} q_{\beta }(j|i)=\frac{m_{ij}+\beta }{\;m_{i}+|𝒜|\;\beta \;}, \end{array}$$ (10)

where |𝒜|=24 represents the possible states. We then compute the divergence rate $D_{KL_{R}}(P\;\|\;Q_{\beta }^{<t})$ as in Eq (9) and refer to this value as the novelty of a piece with respect to all preceding scores. We select a single β=0.1 value by held-out predictive cross-entropy, and we include details of this selection process and a sensitivity analysis in the supplementary materials (S6 Appendix).

Among candidate information-theoretic measures, the KLD rate is the one that isolates novelty relative to history. Mutual information characterizes a sequence’s internal sequential dependency rather than its departure from prior works, while a raw information-content or surprisal score conflates a piece’s own entropy with its novelty. Symmetric divergences such as the Jensen–Shannon divergence quantify mutual dissimilarity with no preferred direction. By contrast, the KLD rate ($D_{KL_{R}}(P\|Q)$) measures the asymmetric excess information per step required to describe a piece under the historical model *Q* rather than under its own model *P*. This directionality mirrors both the forward unfolding of musical transitions and the forward accumulation of historical context, so that novelty relative to the past is precisely the quantity being measured. Other quantitative accounts of harmonic evolution have used instruments of a different kind: one estimates transmission dynamics from time-varying chord-substitution probabilities [27], while another describes tonal organization through the correlation of pitch-class frequency trajectories [36]. Neither provides what we compute here, a directed, per-step measure of how far an individual piece departs from its accumulated past; the correlation in [36], in particular, is symmetric and so carries no preferred direction. $D_{KL_{R}}(P\|Q)$ also differs from the information content used in previous work to quantify novelty [7], in that KLD rate weights transitions by the asymptotic distribution of the elements $\mu_{i}$ (the more repetitive, the less novel) and does not strongly depend on the length of the sequence (see S5 Appendix).

### Comparing composers’ harmonic vocabularies

As mentioned above, we can make use of other information-theoretic measures to gain insights from musical pieces with our framework. One of the questions we could ask is: *How similar are two composers’ harmonic vocabularies?* Which is a way to indirectly quantify or compare composers’ harmonic styles using their harmonic transitions as proxy. This question has no direction (compared to the innovation one), since the resemblance of *a* to *b* is the resemblance of *b* to *a*.

We build a composer’s harmonic vocabulary by aggregating the key transitions of all pieces by a composer *c* into pool counts $m_{ij}^{\left(c\right)}$ (as in the historical pool of Eq (10), but grouped by composer rather than by year) gives the joint transition distribution


$$\begin{array}{c} P^{\left(c\right)}(i\;\to \;j)=\frac{m_{ij}^{\left(c\right)}}{M^{\left(c\right)}},\;(i\;\to \;j)\in 𝒜\times 𝒜,\;M^{\left(c\right)}=\sum_{ij}m_{ij}^{\left(c\right)}. \end{array}$$ (11)

Because the comparison has no preferred direction, we use the Jensen–Shannon divergence [56,57] rather than the directed $D_{KL_{R}}$,


$$\begin{array}{c} D_{JS}\;(P^{\left(a\right)},P^{\left(b\right)})=\frac{1}{2}D_{KL}\;(P^{\left(a\right)}\|\bar{P})+\frac{1}{2}D_{KL}\;(P^{\left(b\right)}\|\bar{P}),\;\bar{P}=\frac{1}{2}(P^{\left(a\right)}+P^{\left(b\right)}), \end{array}$$ (12)

with base-2 logarithms, so that $0\leq D_{JS}\leq 1$ bit and the similarity $S_{ab}=1-D_{JS}\in [0,1]$ is bounded with $S_{aa}=1$. Unlike $D_{KL_{R}}$, the mixture reference $\bar{P}$ is finite without smoothing, and $\sqrt{D_{JS}}$ is a metric [57]. Because a composer’s pool is a finite sample and $D_{JS}$ is biased upward for small samples, we balance the comparison by *rarefying* every composer to a common number of transitions before computing the similarity (see explanation in S8 Appendix).

To display the results we get from this similarity analysis, we use metric multidimensional scaling (MDS), a method that can be interpreted as a map reconstruction from distances. This method has been effectively applied to a set of intercity distances, and it has shown to recover the actual geographic layout of the cities [58,59]. In our case, we use $\sqrt{D_{JS}}$ between composers’ key-transition distributions and let it map each composer’s value to a point, so that nearby points are composers with similar harmonic vocabulary. This means that we would expect for composers that share similar transitions in their harmonic vocabularies to be closer to each other, similarly to a city, composers would be grouped as if they were in neighborhoods (clusters) in a city map. This reconstruction is possible because $\sqrt{D_{JS}}$ behaves like an ordinary straight-line (Euclidean) distance: it can be reproduced exactly by points in a space of sufficiently many dimensions (S8 Appendix), of which we plot the two most informative.

As with any map rebuilt from distances alone, the result is fixed only up to sliding, rotating, or mirror-reflecting it. There is no built-in compass, so “north” may point in any direction; only the relative arrangement of composers carries meaning, that is, who lies near whom and how the neighborhoods separate, not the orientation or the axes themselves. The city example has an external map against which the reconstruction can be checked, but the composer layout has none, since the arrangement is what we are estimating. Instead, we report the fraction of the pairwise distances that the two plotted axes preserve (percentage of JSD variance explained by the 2 dimensions), a measure of how faithfully the flattening onto a page reflects the full set of similarities.

As a complement to the MDS map, which compresses all pairwise similarities into two axes, we also report nearest-neighbor glyphs: for each composer we identify its three most similar composers by the exact $S_{ab}=1-D_{JS}$ on the full rarefied matrix, with bubble area encoding similarity and color the historical period (see S8 Appendix).

### Corpus

We use a set of MIDI files from the Kunst der Fugue website [60]. This dataset has been used in previous studies [7,14,23,35], it consists of a compilation of approximately 18,000 MIDI files from more than 79 Western composers, spanning the years 1200–1950. We retained only those pieces to which we could assign a year of composition (see S10 Appendix), reducing the dataset to 4,638 MIDI files. And after a second filtering process (Markov-1 permutation test), we used a reduced subset of 3,483 pieces for the novelty values. We processed the pieces using a Julia script to extract the information needed to compute the center of effect – such as the number of bars, pitch, and duration of all notes (see S10 Appendix for details).

## Results

In general, the ability to match hand annotations for the majority of chords and keys validates our key-finding methodology (see S3 Appendix for details), especially considering that professional musicians and musicologists may disagree about the key of a bar, even within the well-structured Beethoven quartets. An additional advantage is that the CoE algorithm provides several alternatives, with associated likelihoods, for each bar – since the entropy of this distribution provides a measure of key uncertainty, which is itself an interesting feature of music.

### Key diversity and uncertainty

**Fig 2:**
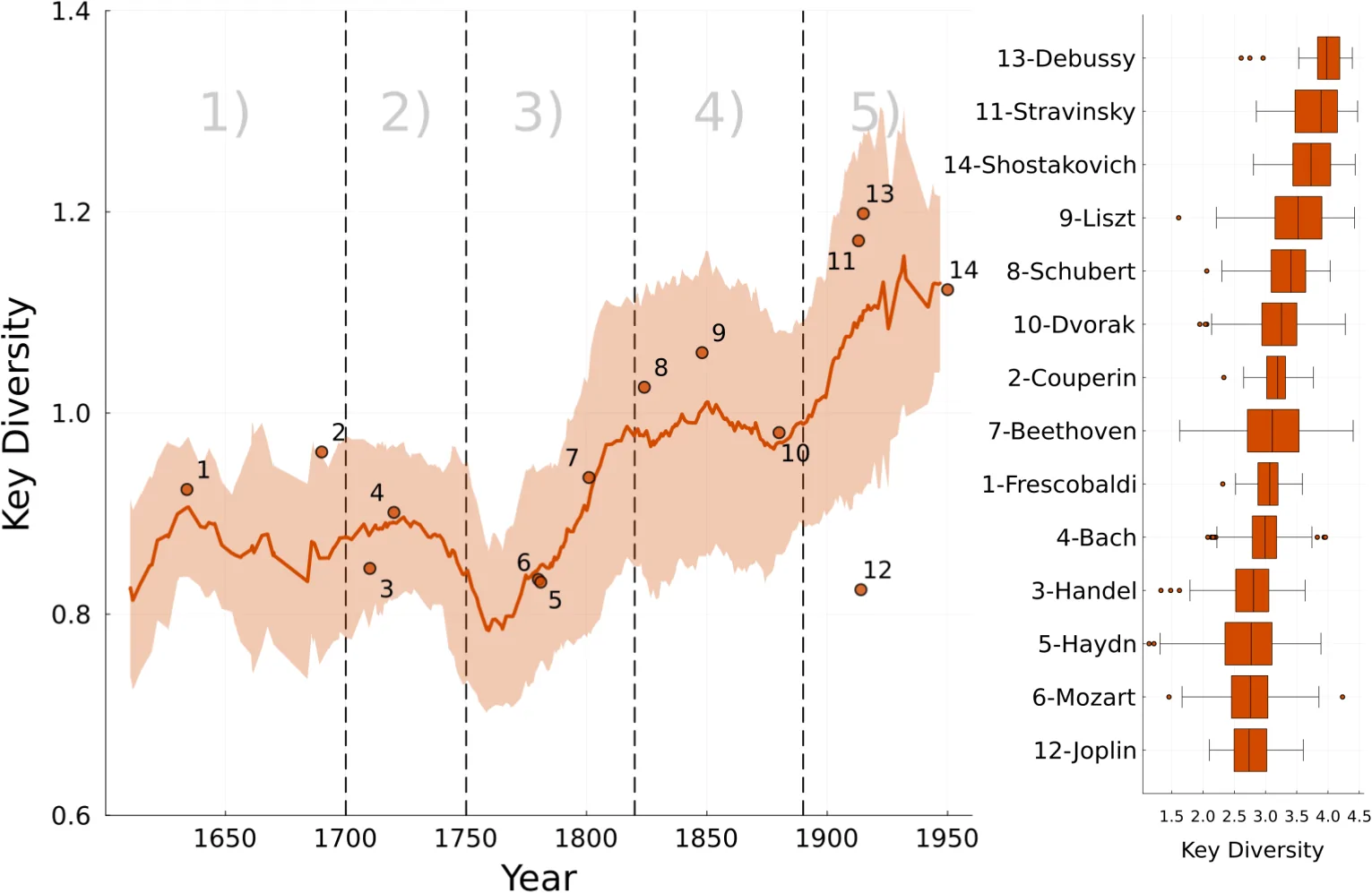
Key diversity. Time series for key diversity across 4,638 musical scores. The solid line represents the median of the distribution of values within a time bin, while the first and third quartiles are represented by the shaded area. All distributions are computed for values that lie within a sliding window of 20 years. Historical periods are denoted by dashed lines, where the labels stand for: **1)** Early/Mid Baroque, **2)** Late Baroque, **3)** Classical, **4)** Romantic, and **5)** Modern.

A time series of key diversity is shown in Fig 2. Throughout, the time series of yearly values are smoothed with a 20-year sliding window solely for visualization of the long-term trend; all statistics are computed on the per-piece values. We observe a trend toward increasing diversity of keys within a piece over time, consistent with results from previous studies showing that many elements in music tend to be more diverse over time [35]. However, in our study, key diversity does not follow a monotonically increasing trend. Rather, the change in diversity varies across specific periods associated roughly with the different eras of classical music defined by musicologists (Early/Mid Baroque, Late Baroque, Classical, Romantic, and Modern).

The increase in key diversity is most evident during the Classical (1750–1820) and Modern (1890–1960) periods. This result for the Classical period agrees with the historical analysis of musical style [61]. Indeed, the Early Classical period saw the development of new instruments that increased the size of orchestras, allowing composers to explore greater tonal modulation and a wider range of musical forms. A similar trend is seen in the Modern period, which is characterized by greater experimentation with modulation and more complex conceptualizations of tonality, or even the rejection of tonality altogether [62].

**Fig 3:**
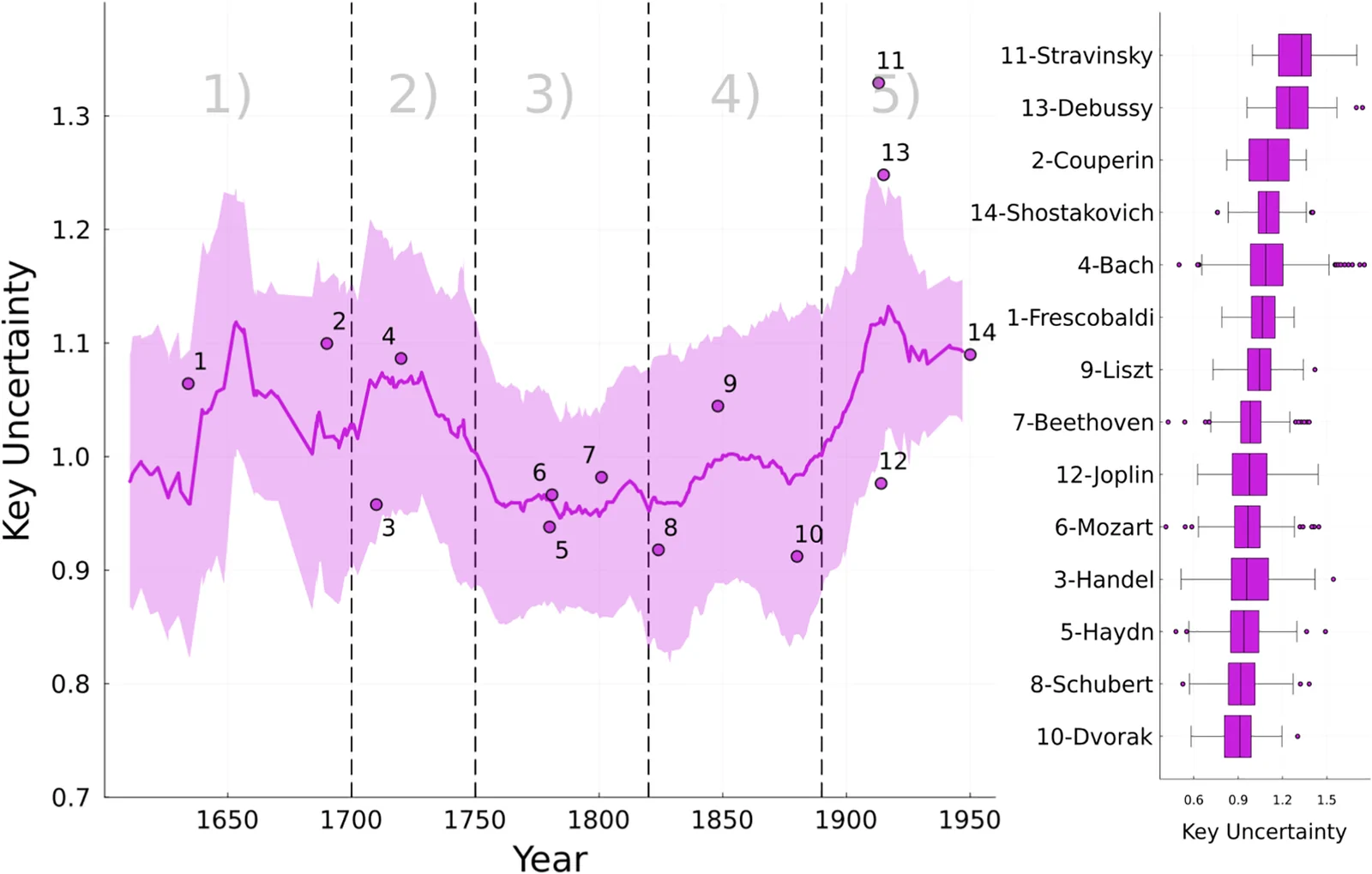
Key uncertainty. Time series for the key uncertainty value, the solid line represents the median of the distribution of values while the first and third quartiles are represented by the shaded area. Regions between dashed lines are the historical periods presented in Fig 2.

Fig 3 shows the time series of key uncertainty for the 4,638 scores in our dataset. The overall trend shows an increase in the uncertainty associated with assigning local keys to bars using the CoE algorithm, over the course of 400 years. This increasing trend in key uncertainty may reflect evolving concepts of tonality. Key uncertainty can be interpreted as how ambiguous a tonal center (key) is, and this ambiguity may be related to tonal ambiguity, proximal tonal tension [63] or, in a more colloquial fashion and perhaps easier to think about: key uncertainty is related to tonal *spiciness*, a food analogy adopted by the musician and YouTuber Adam Neely to describe the property of tonal ambiguity in polytonal music using the circle of fifths as a reference for harmony structure [64]. In our case, the reference is not the circle of fifths, but the spiral array. Long-term changes in key uncertainty are largely consistent with historical features of the Baroque and Classical periods [65]. For instance, in the Baroque period, music was not only polyphonic but also contrapuntal, promoting a higher density of different notes and greater propensity for dissonances. In the Early Classical period the texture of the sound is clearer than in the Baroque period [65], with more emphasis on order and hierarchy, resulting in a homophonic texture with a clear melody above chordal accompaniment – producing a less *crowded* or ambiguous representation of tonality.

**Fig 4:**
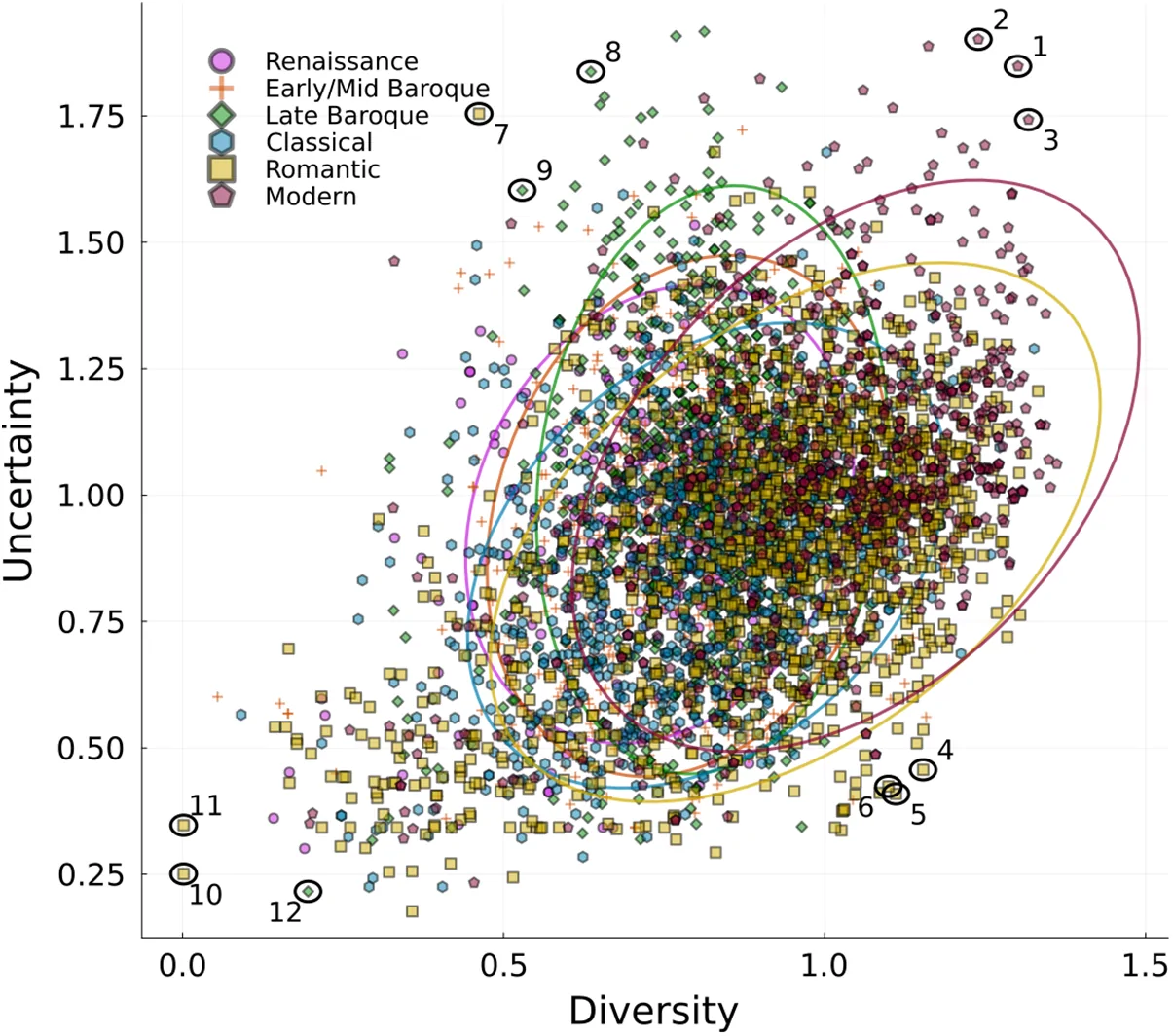
Key diversity vs key uncertainty. Diversity and uncertainty values for individual pieces classified by their historical period. The ellipses are constructed by computing the covariance error with 95% confidence.

While key diversity and key uncertainty represent distinct aspects of a musical piece, the 400-year trend of overall increase holds for both, with the Modern period showing the highest uncertainty and diversity. The relationship between diversity and uncertainty is shown in Fig 4, with a Pearson correlation of ρ=0.38. In the same figure, some of the extreme scores are identified and highlighted with circles; these particular pieces are listed in Table 1. This positive but weak correlation likely reflects a partial overlap between the two measures, both related to tonal ambiguity at different granularities, although part of it may instead arise from key-assignment noise, and disentangling these contributions is beyond the present study.

**Table 1:** Compositions list. List of the pieces highlighted in Fig 4, the number in the first column corresponds to the label in Fig 4. All pieces can be listened to through the YouTube links provided in the Supporting Information (S10 Appendix).

	Year	Composer	Name	Div	Unc
1	1911	Scriabin	Étude Op. 65, No. 1 (Étude in Ninths)	↑	↑
2	1915	Debussy	Étude No. 6: Pour les huit doigts (For Eight Fingers)	↑	↑
3	1915	Debussy	Étude No. 7: Pour les degrés chromatiques (For Chromatic Degrees)	↑	↑
4	1848	Alkan	12 Études in All the Major Keys, Op. 35, No. 11: Posément in B major	↑	↓
5	1860	Liszt	Pater Noster (S.29)	↑	↓
6	1880	Dvořák	Waltz No. 6 in F major, Op. 54	↑	↓
7	1868	Raff	30 Progressive Etudes, WoO. 36, No. 1	↓	↑
8	1741	J.S. Bach	Goldberg Variations, BWV 988: Variation 23	↓	↑
9	1741	J.S. Bach	Goldberg Variations, BWV 988: Variation 30 (Quodlibet)	↓	↑
10	1869	Hymns	The Great Physician Now Is Near	↓	↓
11	1825	Hymns	Brethren, We Have Met to Worship (tune: Holy Manna)	↓	↓
12	1735	Hymns	Jesus Makes My Heart Rejoice (tune: Herrnhut)	↓	↓

Simply listening to the pieces highlighted in Fig 4 – which are selected for having extreme values of key diversity and/or key uncertainty – helps provide an intuitive understanding of what these two measures quantify; accordingly, we include audio references (YouTube links) for each piece in Table 1, representing low uncertainty/low diversity, low uncertainty/high diversity, high uncertainty/low diversity, and high uncertainty/high diversity extremes across the corpus. For example, the pieces that are most uncertain and most diverse in tonality are typically by Modern composers (Scriabin and Stravinsky), while the other extreme (least uncertain and least diverse) corresponds to a simple religious hymn (The Great Physician) that was written to be easy to sing and to remember.

### Innovation in key transitions

We compute a novelty value for each piece using the Kullback-Leibler divergence rate from Eq (9), described in greater detail in the Materials and Methods section. The novelty value is computed using two alternative key representations: the original key and the transposed key. The original key representation refers to the absolute key in a given bar (e.g., C, E, G for major keys and a, e, d for minor keys) while the transposed key corresponds to the key relative to a given reference – in our case, the tonic (i.e., the main key) of the piece. We use these two representations to control for the fact that tonic keys underwent substantial change over the course of the dataset. The transposed novelty measure controls for this gradual variation in tonic keys when computing novelty.

For the representation of novelty after transposition, we use Roman numerals, as in harmonic analysis, mapping every key sequence to a sequence of Roman numerals in which I denotes the global tonic key of the piece (e.g., if C major is the reference: C, E, G → I, III, V; and if A minor is the reference: a, C, E → i, III, V, see S4 Appendix for details). This mapping allows us not only to study sequences of Roman numerals (functional harmony), but also to transpose all pieces and analyze them within the same frame of reference, preventing transitions with the same harmonic relationship from being counted as distinct.

**Fig 5:**
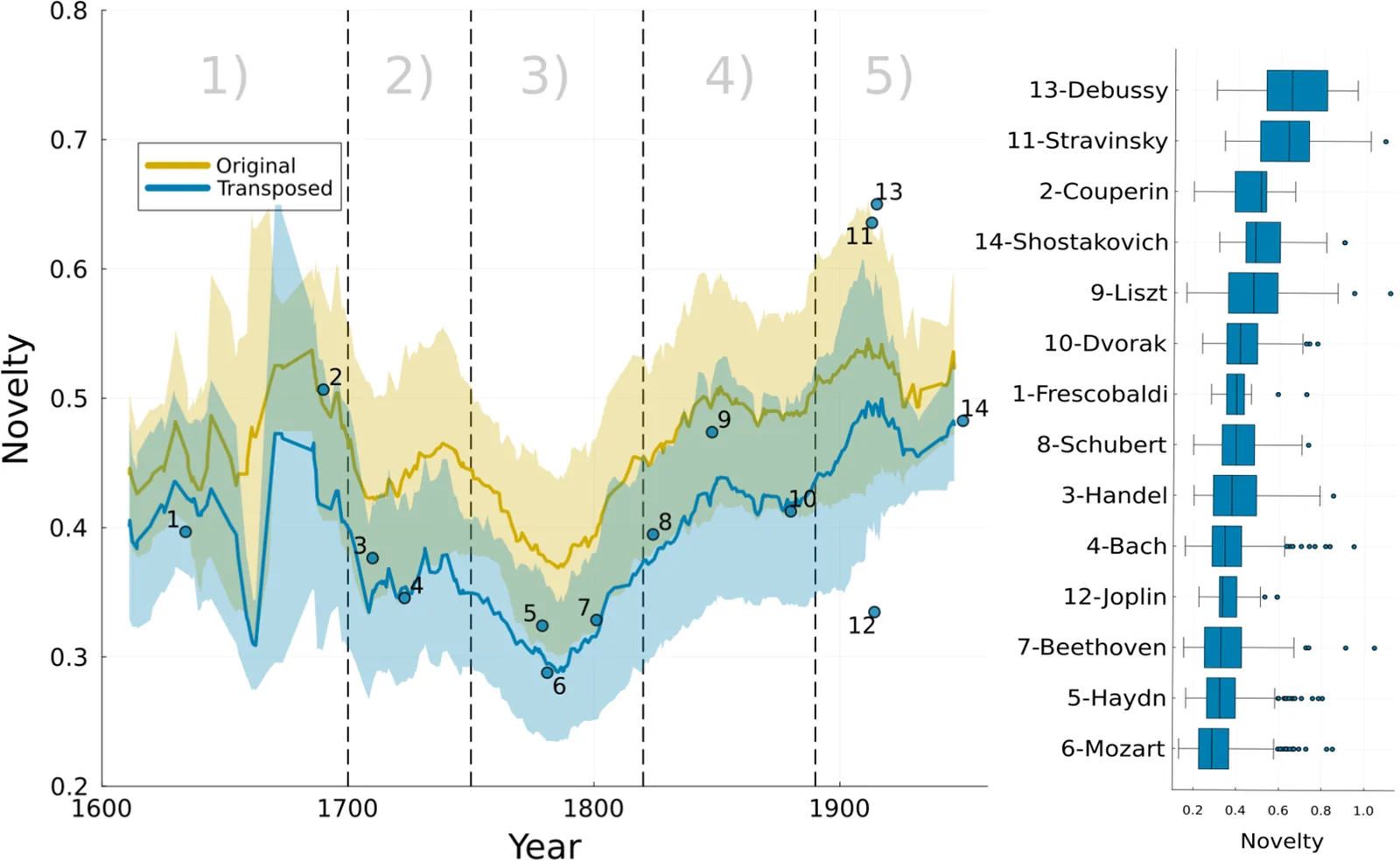
Innovation values. Novelty or innovation values for original key representation (yellow) and transposed or functional harmony representation (blue) for the 3,483 pieces. Values are computed in the same fashion as in Fig 2 with an overlapping window of 20 years.

Results for innovation values in both the original and transposed representations are shown in Fig 5. Lower innovation values for transposed pieces are expected: for example, the transition E → A in a piece with tonic key E becomes I → IV, which is the same as in any other major piece where the tonic transitions to the subdominant, making the piece in E less novel than it would score in the original key representation. The overall trend for original and transposed keys appears to be the same, with lower novelty values for the transposed pieces as expected. We confirmed the authenticity of this global trend with a statistical test (see S5 Appendix).

Fig 5 shows a notable decline in the novelty of harmonic transitions during the Classical period. Although perhaps surprising, this feature has been previously reported, based on a smaller set of pieces and with a representation that considers chords with the same harmonic function as distinct [7].

Another surprising observation is that one of the least innovative composers according to this measure is W. A. Mozart. However, it is well known that Mozart’s style, like Haydn’s, is an archetype of the Classical style, in which clarity, balance, and transparency are hallmarks of his work [66,67]. This conventionality has a compositional basis: the galant idiom was assembled from a shared repertory of stock harmonic schemata [68], so a measure of transition novelty relative to the accumulated historical pool scores its most representative practitioners lowest. It was not until his late active years that he explored chromatic harmony; one example is his well-known String Quartet in C major, also known as the “Dissonance” quartet. Further observations can be made from the results in Fig 5, such as the shift in novelty between the Classical and Romantic periods, where Beethoven (number 7, as indicated in the figure) played an important role in a movement that was consolidated by composers including Schubert and Liszt (numbers 8 and 9, respectively). The increasing trend in harmonic innovation after the second half of the Classical period represents a tipping point that led to continued increases in innovation during the Romantic and Modern periods, which are known for their developments in musical form and harmonic language.

We also computed novelty values separately for pieces in major and minor keys (see Supporting Information, S5 Appendix). We found that although the trends in both major and minor tonalities are similar, they differ considerably in the functional harmony (transposed) representation, with a clear separation during the Classical period. This could indicate that exploration of modulation occurred more frequently in minor keys. This result may reflect the fact that minor tonalities have three different scale patterns compared with major tonalities, which have only one; these three patterns prove a richer space for harmonic modulation in minor keys compared major keys.

A list of the five compositions with the lowest novelty values for each period of time is presented in Table 2. The key sequences in these pieces are highly repetitive, consisting of the most common keys in music theory, such as the tonic (I), the dominant (V) and the subdominant (IV).

**Table 2:** Compositions with lowest novelty values. List of compositions with the lowest novelty values on each period of time, and their first 10 elements. Full sequences are included in Supporting Information (S5 Appendix).

Year	Name	Composer	Novelty value	First 10 elements
1570	O Sacrum Convivium	F. Guerrero	0.2137	I-IV-I-ii-I-IV-I-I-V-IV
1696	String Sonata II Mov 4	D. Buxtehude	0.1969	I-I-IV-I-I-I-I-I-IV-I
1749	Royal Fireworks Suite Mov 4	G.F. Handel	0.1550	I-I-I-I-V-V-V-V-I-I
1798	7 Lander in D	Beethoven	0.1670	I-I-I-V-I-I-I-V-I-I
1867	Les Mois Op. 74 Mov 4	Alkan	0.1888	I-I-I-I-I-I-i-I-I-I

The pieces with the highest novelty values for each period are listed in Table 3. Inspecting the local keys of the first 10 bars of these pieces, we find many keys that do not appear among the common ones in Table 2, with the exception of the piece by Schubert, which starts with common keys before transitioning to less common ones (see S5 Appendix).

**Table 3:** Compositions with highest novelty values. List of compositions with the highest novelty values on each period of time and their first 10 elements. Full sequences are included in Supporting Information (S5 Appendix).

Year	Name	Composer	Novelty value	First 10 elements
1570	Madrigals Book 4 #19	C. Gesualdo	1.72	I-IV-I-vi-V-IV-I-IV-ii-I
1696	Mass for the parishes Mov 14	F. Couperin	1.8597	I-i-i-i-v-IV-I-i-I-IV
1718	WTC I Prelude 14 in F# minor	J.S. Bach	2.1341	i-i-#VI/bVII-i-#VI/bVII-#II/bIII-#II/bIII-v-v-v
1817	Sonata Op. 147 Mov 1	F. Schubert	1.6387	I-I-I-V-I-I-I-I-I-V
1861	Esquisses Op. 63 No. 7	Alkan	1.58	I-I-I-I-vi-#i/bii-v-#i/bii-III

### Comparing composers’ harmonic vocabularies

**Fig 6:**
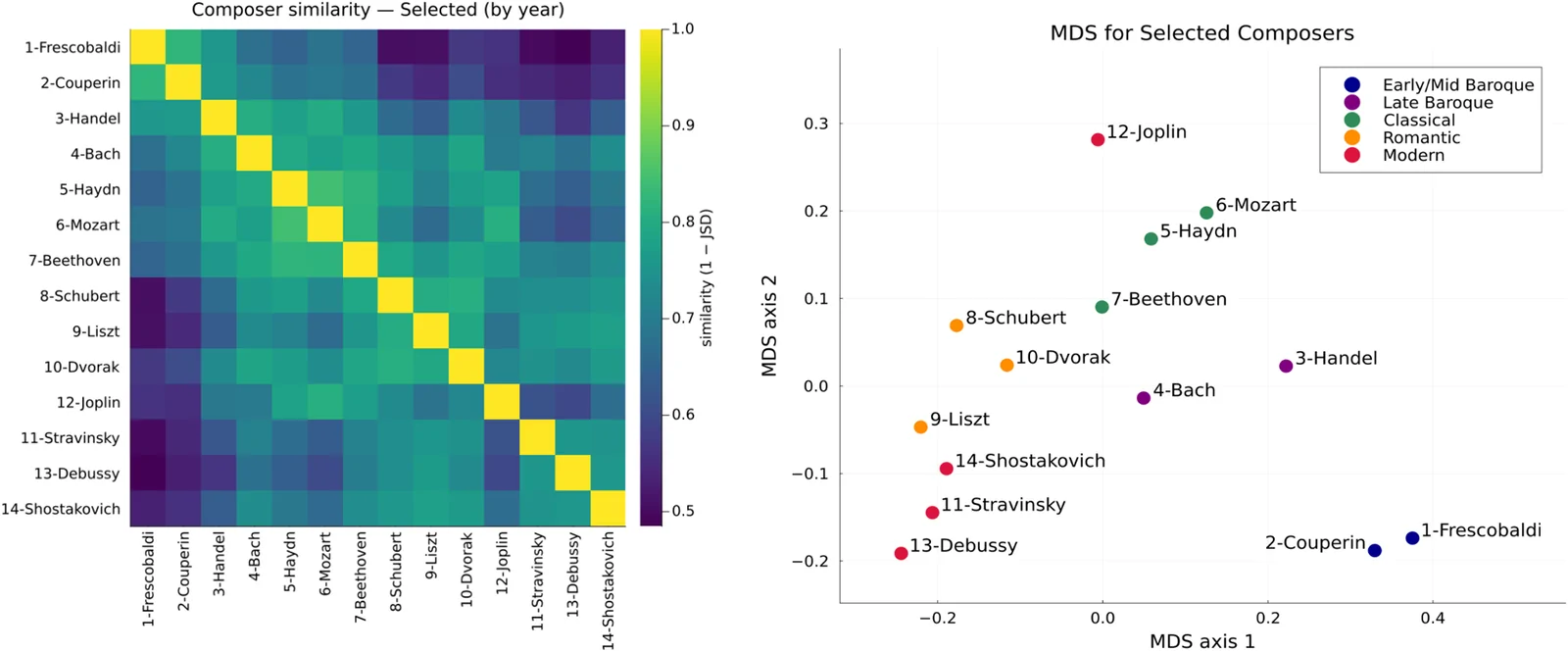
Composer harmonic similarity (selected composers). **Left:** support-normalized similarity $S_{ab}=1-D_{JS}$ between composers’ key-transition distributions, ordered chronologically. **Right:** classical multidimensional-scaling map of $\sqrt{D_{JS}}$; closer points are more similar in harmonic vocabulary, and composers are grouped by historical period. The MSD plot preserves 43% of the pairwise variance.

Applying the symmetric similarity of Eq (12) to the composers of Figs 2, 3 and 5 produces the similarity matrix in Fig 6 (left), and embedding the resulting distances by MDS produces the map in Fig 6 (right). The two plotted axes account for 43% of the variance, a value that reflects the intrinsically high-dimensional nature of harmonic-vocabulary similarity rather than a poor embedding, and is in the range typical for such data [58,69]; we therefore read the map together with the full set of distances (S8 Appendix). In our results, composers group by historical period, with one systematic exception: Scott Joplin lies nearest the Classical composers rather than his early-twentieth-century contemporaries, both on the map and among nearest neighbors in the full $\sqrt{D_{JS}}$ space.

This harmonic conservatism may also account for Joplin’s low novelty value relative to his contemporaries: he is the fourth least innovative composer in our corpus (Fig 5), despite working in the early twentieth century. Because the directed measure of Eq (9) scores each piece against the accumulated historical pool, which by that time already contained the Classical and Romantic repertoire his key transitions resemble, Joplin’s harmonic vocabulary registers as largely familiar rather than innovative, in contrast to contemporaries such as Scriabin and Stravinsky.

**Fig 7:**
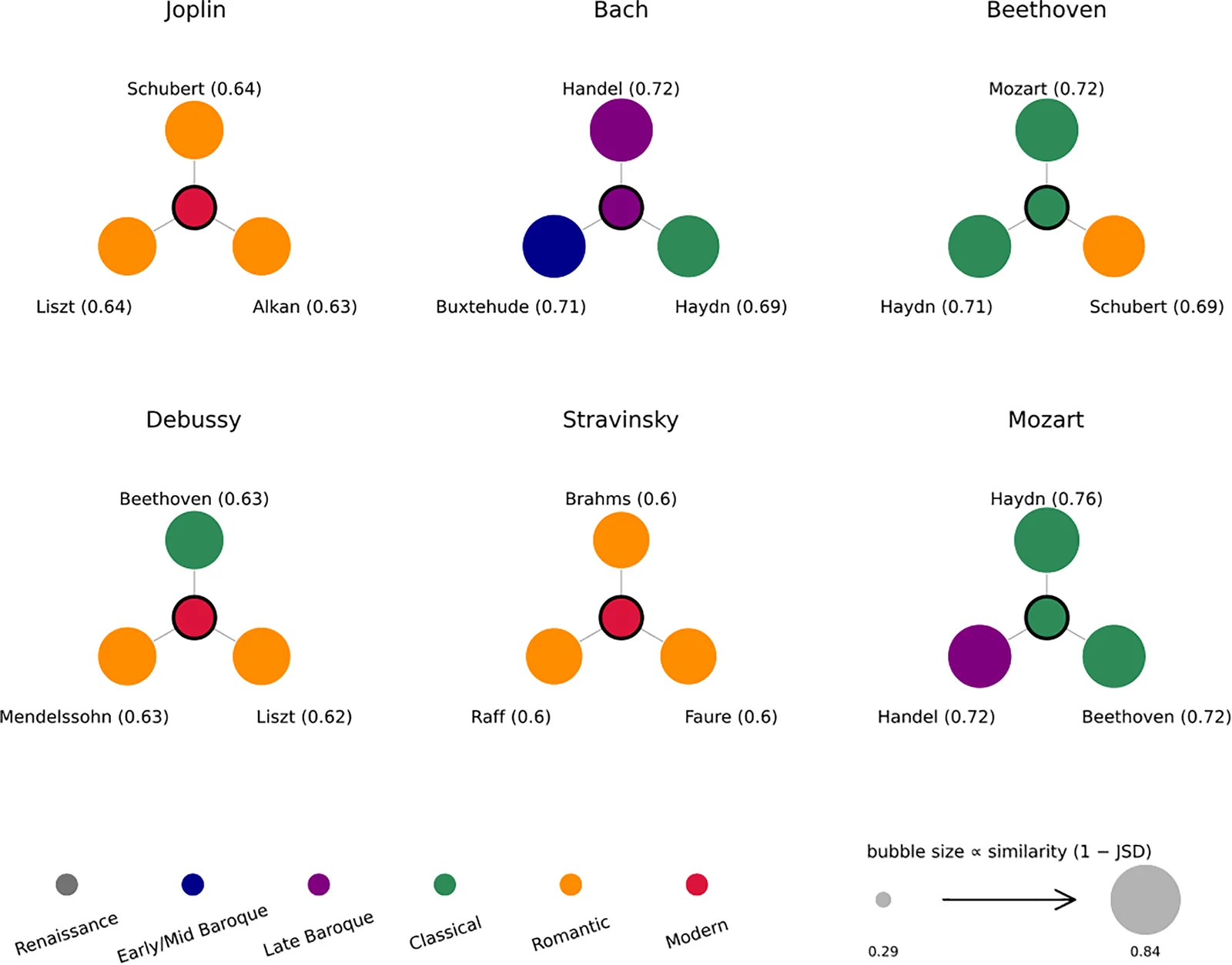
Top-three most similar composers for six representative composers. Each hub (outlined) is surrounded by its three nearest composers by $1-D_{JS}$ on the full rarefied matrix, with bubble area ∝ similarity (scaled to the global range, so bubbles are comparable across panels) and color denoting musical period. Joplin’s three nearest neighbors are Romantic, resolving a relation not legible from the 43% of variance captured by the MDS map in Fig 6.

The two-dimensional MDS map preserves only 43% of the pairwise variance, enough to recover the coarse grouping by period but not the exact ordering of each composer’s closest neighbors. To expose that ordering we read it directly from the full rarefied $\sqrt{D_{JS}}$ matrix and plot, for six representative composers, the three most similar composers to each (Fig 7). For Joplin the three nearest are Romantic composers, not Classical ones, even though the map seats him beside the Classical cluster. This reinforces the result we get from the map, that states that Joplin sits away from his early-twentieth-century contemporaries and toward earlier harmonic practice, while the nearest-neighbor glyph identifies that practice, at the level of individual neighbors, as Romantic. Both agree with his documented adoption of European classical and Romantic harmonic idioms in classic ragtime [70,71]. This conservatism is specifically harmonic. Ragtime’s defining innovation was rhythmic, a syncopated style set over conventional European harmonic and formal schemes [72], so a key-transition measure scores Joplin as familiar on the axis where ragtime was least novel and does not register the rhythmic dimension that carries its originality.

A second relation legible in the nearest-neighbor glyphs is the proximity of J. S. Bach to Buxtehude, which aligns with a documented line of influence: in 1705 the young Bach travelled on foot from Arnstadt to Lübeck to study Buxtehude’s art [73,74], and Buxtehude’s contrapuntal and ostinato idioms are widely held to inform Bach’s own [74]. The agreement is suggestive rather than direct, since our similarity is computed on bar-level key transitions rather than the organ and fugal textures through which that influence is usually traced.

## Discussion

The spiral array is a perceptually grounded model of tonality, a helical embedding of notes, chords, and keys in Euclidean ${\mathbb{R}}^{3}$ derived from the Tonnetz, in which Euclidean proximity tracks harmonic proximity [45]. Although, other richer geometric and cognitive models of tonal space exist [8,10,11], in this study, we show that a modified version of the spiral array with features like octave-reduced and enharmonic equivalence, combined with the bar-level coarse-graining of the score can be used as framework along with information-theoretical measures to quantify harmonic features and providing statistical insights in a systematic way.

The center of effect algorithm has been shown to identify a meaningful notion of local key in both tonal and even non-tonal music [43]. Our implementation performs well in matching a gold standard corpus of manual annotations by musicians. The broader application of the CoE to local key identification may be useful not only to musicians who want to explore artistic development, but also to the scientific community that is interested in the quantitative description and development of variation in tonality and tonal tension, within a piece or across different pieces and time periods.

One disadvantage of this approach is the need to select a window size over which the center of effect is computed. We have chosen a window size of one bar, but this is a somewhat arbitrary decision; indeed, bar length varies over time and within pieces. The local key may change in a meaningful way even within a single bar, and variation across two or three bars may also be meaningful. The issue is visible in the example shown in Fig 1, where some bars may be assigned two plausible different keys (e.g., in bar #2 there are two possible keys: C and D); we choose the most likely key according to its distance to the center of effect. Addressing this complexity would require a different approach involving automatic segmentation, where the point of key change is determined by a parameter expressed as a function of the distance between centers of effect calculated over different time windows [41]. Such an implementation would ideally reduce the dispersion in uncertainty and diversity values shown in Figs 2 and 3, but this remains a topic for future work.

A related limitation concerns temporal scale. Our first-order Markov description of bar-to-bar key transitions is a choice about local transition structure, not a claim that harmony is memoryless: a first-order model and genuine long-range dependence are not mutually exclusive, and the bar-level coarse-graining already folds some of the longer-range structure of the note-level sequence into single-step transitions between local keys. Even so, the framework is deliberately scoped to harmonic motion at the bar level and does not represent large-scale musical form. Two pieces with similar local key-transition statistics but very different global architecture would be treated as similar under our measures, and our ΔBIC and permutation tests (S7 Appendix) validate the order-1 description of these local transitions rather than the higher-order organization of the whole work. Extending the representation to capture form explicitly is left for future work.

The structure and evolution of rhythmic, melodic and harmonic properties have been explored previously in several studies of large corpora [13,14,19,22,23,75,76], mostly using time series analysis methods to describe concepts such as scaling, predictability, nonlinearity and reversibility within a given musical piece.

In contrast to most of the time-series methods used in these studies, we have focused our analysis on coarse-graining a musical score, but in a way that retains specifically musical information. That is, we represent a piece as a series of transitions between local keys, from bar to bar. Using our definition of local key, we can assign information-theoretic concepts to musical pieces, such as diversity and average uncertainty, based on an intrinsically musical representation of the piece. In particular, key uncertainty quantifies the degree of ambiguity when a set of notes is assigned a key; this uncertainty can also be related to how unpredictable a given set of notes is – that is, how difficult it is to anticipate what note will follow. Similar types of unpredictability have been quantified in [23], where an overall increasing trend toward greater unpredictability of notes over time was observed, with Shostakovich being one of the most unpredictable composers at short time frames. Those findings agree with our results for long-term trends in key uncertainty, which places Shostakovich among the composers with the highest key uncertainty. These results are also consistent with the historical development of tonality, which was not formally defined until 1722 in Jean-Philippe Rameau’s *Treatise on harmony* [28]. The decline in uncertainty during the Classical period reflects a time when tonal conventions became adopted by many composers, shifting the texture from the polyphonic and contrapuntal forms of the Baroque period toward clearer, homophonic forms defined by a melody and subordinate chordal accompaniment. The subsequent increase in uncertainty in the Romantic and Modern periods reflects the evolution of the concept of tonality in the Late Romantic and Early Modern periods, with composers such as Bruckner, Tchaikovsky, Mahler, Scriabin, Wagner, and Strauss. The last two are known for having “furthered the musical language of Opera taking tonality itself to breaking point” [77]. Schoenberg described this phase of tonality as “fluctuating” or “suspended”, implying that it was undecided or ambiguous [29]. Finally, in the Modern period, harmonic progressions became more unpredictable, making tonality even more ambiguous. As Meyer described “the increased use of the ambiguous chords, the less probable harmonic progressions, and the more unusual melodic and rhythmic inflections ... the felt probabilities of the style system had become obscure; at worst, they were approaching a uniformity which provided few guides for either composition or listening” [50].

Key diversity within a piece also has a close relationship with tonality, as it is closely related to harmonic function (functional harmony). Trends from the Classical, Romantic, and Modern periods largely coincide with those in uncertainty. However, one important difference between uncertainty and diversity is that the increase in diversity is more evident in the Late Classical period, indicating increasing modulations within a piece. This result agrees with the historical observation that in the Late Classical period composers began to explore different harmonic transitions and innovations, as the Industrial Revolution contributed to the expansion and diversification of orchestras, giving composers more opportunities to explore different styles and sounds [78–80]. In the scientific literature, few studies are directly related to key uncertainty and diversity, whereas more studies explore the distribution of notes, chords, or other tokens in analogy to language.

There is a substantial body of work establishing empirical laws such as Zipf’s and Heap’s laws for such musical features. One such study considers the concept of vocabulary richness, defining an element (or token) as the set of different notes played during a beat; and the authors find an increasing linear trend in vocabulary richness over time [35]. A broadening of harmonic vocabulary over historical time has also been reported with other instruments: [36] find that composers explore progressively wider regions of the line of fifths, and [27] observe rising chord-vocabulary entropy in some twentieth-century popular genres. These trends point in the same direction as our increasing key diversity, but both quantify population- or period-level vocabulary, through static pitch-class co-occurrence and aggregate chord substitution rates respectively, rather than the directed, per-piece novelty relative to accumulated history that our measure captures. Similar historical organization appears when composers are compared directly by their key-transition vocabularies: composers group by period in harmonic similarity space, so the period structure visible in the temporal trends is also recoverable from the transitions themselves. Scott Joplin is the informative exception, with his nearest neighbors lying among earlier Classical and Romantic composers rather than his contemporaries, a harmonic conservatism consistent with his low novelty value.

Our study identifies the Classical period as a tipping point for novelty in harmonic transitions. While this does not mean that there was no innovation prior to this period, it provides a quantitative account of the evolution of harmony discussed in qualitative terms by many music historians [50,78–80]. The increase in key diversity and modulation plays an important role in our computation of novelty, but it is not the only factor: recurrent transitions reduce a piece’s novelty under our metric, so a piece with novel transitions can still score low if it is highly repetitive. Because the novelty value compounds several contributions at once, the diversity of transitions, their resemblance to the accumulated historical pool, and the degree of repetition within the piece, a single score should not be read as evidence of one mechanism: a low value may reflect harmonic conservatism, internal repetition, or both, and these are not separable from the scalar alone. The contribution of repetition is visible in the entropy rate of each piece, which shows no particular trend over time (see Supporting Information, S5 Appendix), suggesting that some degree of repetition serves a functional role in music. Indeed, Schoenberg describes music as a balance between repetition and surprise [29].

Ours is not the first study to quantify novelty in musical scores. The authors of [7] address a similar question, although they represent a piece as a sequence of chords without attempting to determine the local key of each bar. That study was also constrained to a considerably smaller corpus (<1000 pieces) and fewer composers (approximately 20). Notably, in some cases all of the pieces by a given composer were assigned the same year in that study. Although our results share some similarities with those of [7], there are also notable differences. For instance, Clementi is one of the least novel composers according to [7], while Mozart is the least novel in our study. Both approaches show a decline of novelty during the Classical period, although it is not clear whether the explanation is the same, because the lack of transposition and octave reduction in [7] produces a fundamentally different novelty measure that is influenced by variation in the tonic across pieces. In our framework, transposition is important for capturing features of harmonic relevance: a chord played in a higher octave is not more novel than in a lower one in our analysis (unlike in [7]); and the same piece played in a different key is not considered novel under our measure, unlike in [7].

Although we have been able to quantify novelty in harmonic transitions and identify historical trends, much remains to be understood about the underlying processes of innovation in music. Innovation in harmonic transitions may arise from changes at the level of individual elements: a new local key can emerge when the linear combination of notes in the center of effect is perturbed – for instance, by adding a note or altering the duration weights – potentially shifting the result to a neighboring key in the spiral array. This mechanism has a natural connection to the theory of the “adjacent possible” [81,82], in which innovation occurs by exploring states that are close to previously visited ones in a defined space. Future work along these lines would benefit from expanding the representation beyond local keys. A natural next step would be to model joint transitions of key and rhythmic structure, which would preserve tractability while capturing interactions between harmonic and rhythmic innovation. More broadly, incorporating melodic motifs, dynamics, and orchestration into the representation would bring the analysis closer to the full complexity of musical composition, although at the cost of increased dimensionality. Most importantly, such extensions would also require a substantially larger and more reliable corpus than currently available. MIDI files, while accessible, are often inconsistent in quality and lack standardized metadata; and the subset of pieces with verifiable dates of composition remains limited.

Extending a quantitative study of harmony beyond Western music with this approach will require beginning at a layer below the one we work at. The spiral array assumes a tonal system, the diatonic keys and the circle of fifths, and so it can quantify harmony only once that system is in place, it cannot itself explain why a culture discretizes pitch as it does. That question is perceptual and cognitive, and cross-cultural work on how listeners hear and organize sound is what would be required for this missing layer [83]. Statistical-mechanics models have shown somewhat effective in recovering these discretization of pitch classes, Berezovsky derives the emergence of discrete pitch sets, including the 12-fold octave division, as an ordered phase of sound by the same formalism that describes phase transitions in physical systems [84]. Such models are perceptually grounded in that their energy is built from sensory dissonance, an auditory mechanism that is plausibly shared across cultures, but they also assume that dissonance is minimized and that octaves are equivalent, and cross-cultural studies show neither assumption to be universal: listeners can perceive roughness without preferring consonance, and octave equivalence appears to depend on enculturation [85,86]. A plausible approach is therefore hierarchical, with perceptually grounded models accounting for how cultural constructs such as scales and tonality arise, but with the perceptual primitives themselves measured per culture rather than assumed, and geometric models such as the spiral array quantifying harmony once those constructs are fixed. Our framework lives only in the upper level of this hierarchy, and only for the Western tonal system it was built on. A better approach will require not only richer and better-curated corpora but a more general representation of musical structure, a shared, tradition-neutral language in which different types of music can be written down and compared, which remains a central obstacle to a cross-cultural account of musical evolution.

The approach we have presented is deliberately simple, yet a quantitative perspective becomes powerful once its measures are given a fixed frame of reference. By grounding every quantity in perceived tonal relationships, the framework stays tied to human experience and offers a shared language in which the regularities of music can be examined, abstractions that serve the anthropological study of music as a window onto culture, cognition, and the relationships through which it is made and shared.

## Supporting information

S1 Appendix Spiral representation: Definitions and parameters. Definitions of pitches, major and minor chords, and major and minor keys in the spiral array, with the parameter values used in the model.
(PDF)

S2 Appendix Center of effect definitions and CoE key-finding algorithm. Alternative weighting schemes for the center of effect and the full CoE key-finding algorithm with enharmonic equivalence.
(PDF)

S3 Appendix Validation of the CoE algorithm. Comparison of CoE against hand-annotated chord and key data for Mozart’s piano sonatas and Beethoven’s string quartets, including the Krumhansl–Schmuckler algorithm.
(PDF)

S4 Appendix Local keys to modulation in functional harmony. Mapping from local-key sequences to Roman-numeral (functional-harmony) sequences used for the transposed novelty representation.
(PDF)

S5 Appendix Kullback–Leibler divergence rate. Derivation of the KL divergence rate for stochastic matrices used as the novelty measure, with the complementary length-independence and significance tests.
(PDF)

S6 AppendixParameter β selection and sensitivity analysis.Held-out predictive selection of the smoothing parameter and robustness checks of the reported structures.(PDF)

S7 Appendix Markov-order tests. Information-criterion order selection and the penalty-free permutation test for order-1 structure, with the retention criterion.
(PDF)

S8 Appendix Composer harmonic similarity. Pooling of per-composer key transitions, the symmetric Jensen–Shannon similarity, support normalization by rarefaction, and the classical-MDS embedding.
(PDF)

S9 AppendixKey-uncertainty parameter λ.Calibration of the key uncertainty parameter λ from the spiral-array geometry, the 12-key local neighborhood and cutoff invariance, bounds on the uncertainty, and robustness of the reported trends to λ.(PDF)

S10 Appendix Dataset and data processing. MIDI corpus curation, processing pipeline, and additional dataset statistics.
(PDF)
