# Supplementary material for: Quantifying the evolution of harmony and novelty in western classical music
Source: PLoS One. 2026 Aug 20;21(8):e0356548. doi: 10.1371/journal.pone.0356548 (PMC13492798; doi:10.1371/journal.pone.0356548)
Supplement: Supplementary file 1 [file pone.0356548.s001.pdf]

S1 Spiral representation: definitions and parameters

We use the Spiral representation introduced by E. Chew [1] on which each pitch, chord and key is represented by a point in $\mathbb{R}^3$. This representation is given by fifths: a pitch k and a pitch $k+n$ are separated by n fifths. Pitches are defined as:

$$\vec{p}(k) = \begin{bmatrix} x_k \\ y_k \\ z_k \end{bmatrix} = \begin{bmatrix} r \sin \frac{k\pi}{2} \\ r \cos \frac{k\pi}{2} \\ kh \end{bmatrix}, \tag{S1}$$

where r and h are fixed: $r = 1$ and $h = (2/15)^{1/2}$. The major and minor chords are constructed as linear combinations of pitches:

$$\vec{C}_M(k) = w_1\vec{p}(k) + w_2\vec{p}(k+1) + w_3\vec{p}(k+4), \tag{S2}$$

and

$$\vec{C}_m(k) = u_1\vec{p}(k) + u_2\vec{p}(k+1) + u_3\vec{p}(k-3), \tag{S3}$$

where $k+1, k+4$ and $k-3$ are the tonic, major third and minor third respectively in the spiral representation. In the same fashion, the major key representations are defined from the major chords:

$$\vec{T}_M(k) = \omega_1\vec{C}_M(k) + \omega_2\vec{C}_M(k+1) + \omega_3\vec{C}_M(k-1), \tag{S4}$$

where $k+1$ and $k-1$ are the dominant and the subdominant chords, the minor key representations are given by:

$$\begin{aligned} \vec{T}_m(k) = \nu_1&\vec{C}_m(k) \\ &+ \nu_2[\theta_M\vec{C}_M(k+1) + (1-\theta_M)\vec{C}_m(k+1)] \\ &+ \nu_3[\theta_m\vec{C}_m(k-1) + (1-\theta_m)\vec{C}_M(k-1)], \end{aligned} \tag{S5}$$

this definition requires two extra parameters to weight the different scale patterns that are presented in minor keys (natural, harmonic minor and melodic minor) given by the major and minor dominant and subdominant chords, the parameters θ_M and θ_m are set equally: $\theta_m = \theta_M = 0.75$.

Each weighting vector in the pitch, chord and keys definitions follow $w_1 \geq w_2 \geq w_3 > 0$ and $\sum_i w_i = 1$, to consider some notes (or chords) more important than the others (e.g. the tonic in a chord would be more important than the fifth and both more important than the third). For convenience, all these vectors are given equal values:

$$\vec{\nu} = \vec{\omega} = \vec{u} = \vec{w} = \{0.536, 0.274, 0.19\}$$

All parameters are discussed in detail in the Appendix A (model calibration) of Chew's book about computational and mathematical modeling of tonality [1].

References

1. Chew E. Mathematical and Computational Modeling of Tonality. vol. 204 of International Series in Operations Research and Management Science. Boston, MA: Springer US; 2014. Available from: `http://link.springer.com/10.1007/s11071-019-04865-2http://link.springer.com/10.1007/978-1-4614-9475-1`.

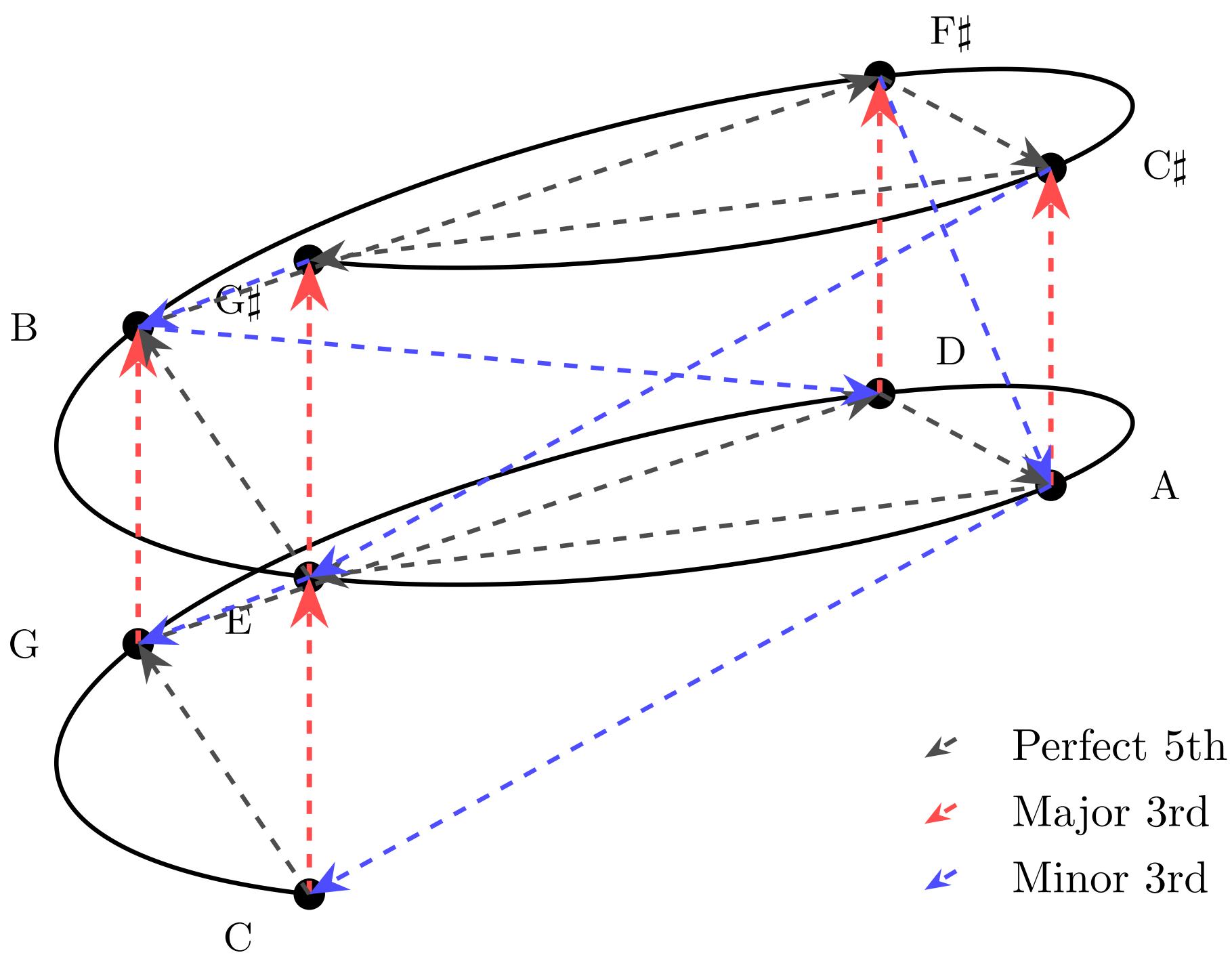

Fig S1. Illustration of musical notes ordered by fifths in the spiral array representation. The color of the dashed lines indicates the harmonic relationship between the notes.

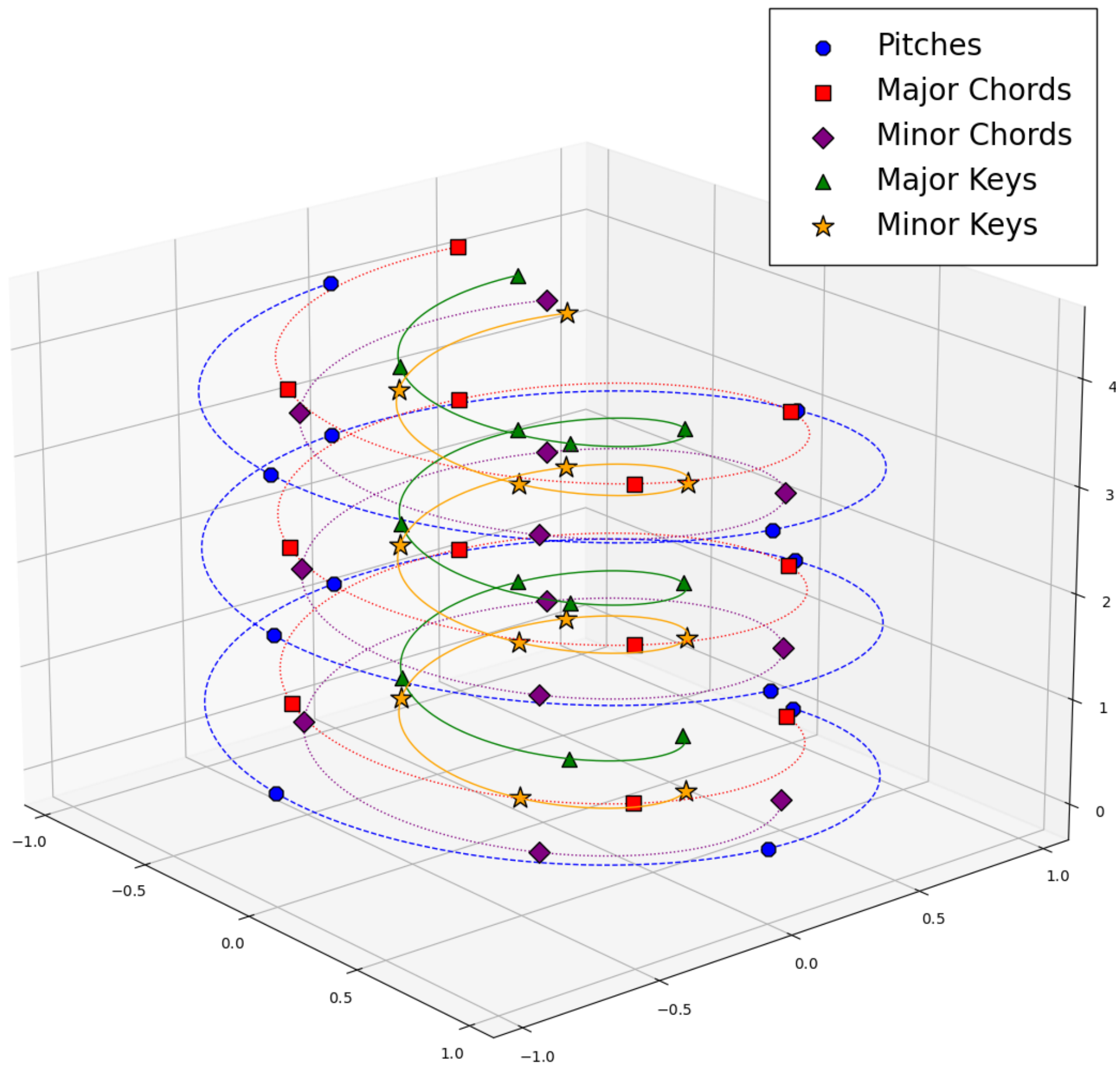

Fig S2. Spiral representation. Pitches, major and minor chords and keys in the geometric representation of the Spiral Array.
