# Supplementary material for: Quantifying the evolution of harmony and novelty in western classical music
Source: PLoS One. 2026 Aug 20;21(8):e0356548. doi: 10.1371/journal.pone.0356548 (PMC13492798; doi:10.1371/journal.pone.0356548)
Supplement: Supplementary file 2 [file pone.0356548.s002.pdf]

S2 Center of effect definitions and CoE key-finding algorithm

Center of effect

The center of effect $\vec{c}_e$ is the location in $\mathbb{R}^3$ for the *center of mass* of a set of notes $\mathcal{N} = \{p_1, p_2, ..., p_N\}$ defined as a linear combination for the position of the pitches:

$$\vec{c}_e = \sum_{i=1}^{N} \omega_i \vec{p_i}, \tag{S6}$$

where ω_i are the normalized ($\sum \omega_i = 1$) coefficients (weights) of each note. These coefficients can be defined in different ways since there are some notes that could be more important than others to define the key of the note sequence.

Duration weighting

The first approach for the *importance* of a note is its duration, following the reasoning that a more lasting note will be more relevant for the harmony in the set of pitches. Given a set of durations $\{d_1, d_2, ..., d_N\}$, the center of effect can be written as:

$$\vec{c}_e = \frac{1}{\sum_i d_i} \sum_{i=1}^{N} d_i \vec{p_i}, \tag{S7}$$

where the term $\sum_i d_i$ is to guarantee that the coefficients are normalized.

Pitch height weighting

A different way of weighting the contribution of each note to the center of effect is to consider the height of the pitch: lower pitches are commonly used as the basis of the key (or chord) being normally the fundamental (tonic) note the most frequent in the low pitches. Given a set of pitch height-weights $\{h_1, ..., h_n\}$, the center of effect can be re-written as:

$$\vec{c}_e = \frac{1}{\sum_i h_i} \sum_{i=1}^{N} h_i \vec{p_i}, \tag{S8}$$

where the weights h_i can be assigned with a function of the height of the pitch, given a sequence of pitches $\mathcal{N} = \{p_1, p_2, ..., p_N\}$ each weight is computed by:

$$h(p) = 1 + (\gamma - 1) * (p - \max(\mathcal{N})/(\min(\mathcal{N}) - \max(\mathcal{N})), \tag{S9}$$

where the functions $\max(\mathcal{N})$ and $\min(\mathcal{N})$ correspond to the maximum and minimum value in the set $\mathcal{N}$.

Beat weighting

Other property that can be used for the coefficients of the center of effect is the number of beat on which the note is in the time signature of the bar. This feature considers the rhythmic feature since not all notes are equal in the bar; some notes are usually more emphasized than others depending on their location. Therefore depending on the time signature and the location of each note the center of effect can be written as:

$$\vec{c}_e = \frac{1}{\sum_i b_i} \sum_{i=1}^{N} b_i \vec{p_i}. \tag{S10}$$

Multiple weighting

Finally, the coefficients ω_i can be constructed as a combination of any of the weightings mentioned above; by combining all three weightings for the center of effect we obtain $\omega_i = d_i h_i b_i$.

CoE key-finding algorithm

The CoE key finding algorithm uses the *center of effect* as a reference to compute the *most likely key* for a sequence of notes. Given a sequence of pitches $\mathcal{N} = \{\vec{p}_1, \vec{p}_2, ..., \vec{p}_N\}$ its center of effect ($\vec{c}_e$) is computed, and the most likely key is given by:

$$\arg\min_{T \in \boldsymbol{T}} ||\vec{c}_e - \vec{T}||, \tag{S11}$$

which corresponds to the key T for which the Euclidean distance is minimum, where $\boldsymbol{T}$ is the set of major and minor keys: $\boldsymbol{T} = \{\boldsymbol{T}_M(k) \forall k\} \cup \{\boldsymbol{T}_m(k) \forall k\}$ defined in equations S4 and S5. The associated likelihood of the most likely key, as well as all alternative keys, is given by Eq (**??**).

Computing the center of effect with enharmonic equivalence

To compute the center of effect, our approach considers notes that span multiple octaves as if they were in the same octave and treats enharmonically equivalent notes as the same pitch class. We achieve this through a circular-centered reordering algorithm that maps all notes to a single octave representation while preserving their relationships to a weighted circular mean.

The core idea is to take a collection of notes (which may be in different octaves), project them onto a single octave using modulo 12, and then *unwrap* them into linear positions that minimize their distance to the circular mean. This ensures that notes are grouped appropriately around the tonal center.

Given a set of notes $S = \{n_1, n_2, \ldots, n_k\}$ where $n_i \in \mathbb{N}$, we transform them into linear positions $L = [p_1, p_2, \ldots, p_k]$ where $p_i \in \mathbb{Z}$ through the following steps:

First, we project all notes onto a single octave using modulo 12:

$$\pi(n) = n \bmod 12$$

This gives us pitch classes in $\mathbb{Z}_{12} = \{0, 1, 2, ..., 11\}$, where each value represents a pitch class regardless of octave.

Next, we compute the circular weighted mean of these pitch classes. Since pitch classes form a circle (where 11 is adjacent to 0), we use circular statistics:

$$\bar{X} = \frac{\sum_{v \in V} f(v) \cdot \cos(2\pi v/12)}{\sum_{v \in V} f(v)} \tag{S12}$$

$$\bar{Y} = \frac{\sum_{v \in V} f(v) \cdot \sin(2\pi v/12)}{\sum_{v \in V} f(v)} \tag{S13}$$

$$\mu_c = \frac{12 \cdot \text{atan2}(\bar{Y}, \bar{X})}{2\pi} \bmod 12 \tag{S14}$$

where V is the set of unique pitch classes and $f(v)$ is the frequency (number of occurrences) of pitch class v.

Finally, we *unwrap* the circular positions into linear space. For each pitch class v, we determine its linear position based on its circular distance to μ_c:

$$\phi(v, \mu_c) = \begin{cases} v - 12 & \text{if } v - \mu_c > 6 \\ v + 12 & \text{if } v - \mu_c < -6 \\ v & \text{otherwise} \end{cases}$$

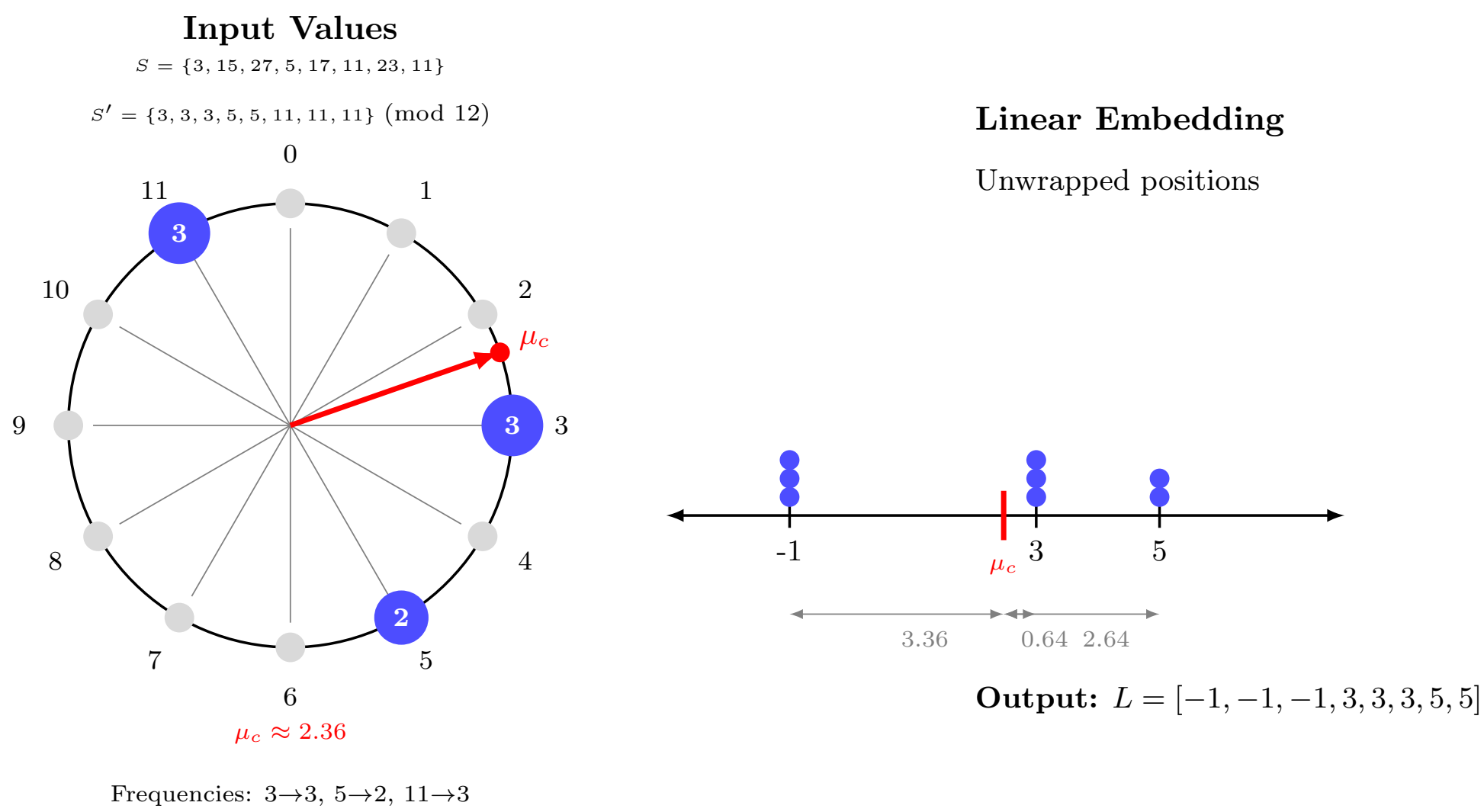

Fig S3. Illustrative example for the sequence $S = \{3, 15, 27, 5, 17, 11, 23, 11\}$. First, the sequence is mapped to modulo 12 ($S' = \{3, 3, 3, 5, 5, 11, 11, 11\}$), the algorithm follows a circular mapping to reorder the sequence centered on its circular mean μ_c.

References
