# Supplementary material for: Quantifying the evolution of harmony and novelty in western classical music
Source: PLoS One. 2026 Aug 20;21(8):e0356548. doi: 10.1371/journal.pone.0356548 (PMC13492798; doi:10.1371/journal.pone.0356548)
Supplement: Supplementary file 3 [file pone.0356548.s003.pdf]

S3 Validation of the CoE algorithm

In order to evaluate the performance of our implementation for the CoE key finding algorithm, we compared our results with hand-annotated information by experts from two sets of musical pieces: Mozart's piano sonatas and Beethoven's string quartets. These collections were created and published by the Digital and Cognitive Musicology Lab at the EPFL [1]. Two different tests were implemented on two different scales: very local and local. This is meant to evaluate how good the CoE model is at guessing harmony information at different time scales, with the bar as the basic unit of analysis in our study.

Chord accuracy by bar

To compare chord accuracy by bar, we divided the piece by bar: non-overlapping windows of 1-bar size. Then we computed the center of effect and calculated its distance to each of the chords in the spiral representation. The process is equivalent to key finding, but using the location for the chords instead:

$$\underset{C \in \mathbf{C}}{\arg\min} \, ||\vec{c}_e - \vec{C}||, \tag{S15}$$

we compared the hand annotated notes for chords on each of the bars for each piece. If the *guessed* chord C_g for bar m was found in the set of annotated chords $\mathbf{C_m}$ for the same bar, we consider it a match or an accurate guess. We did this evaluation considering the first, second, and third guesses from our CoE implementation and considered a match or an accurate guess if the intersection of the set of CoE guesses $\mathbf{C_g}$ and the annotated chords $\mathbf{C_m}$ is non-empty.

Key accuracy by region

We implemented the Krumhansl-Schmuckler (KS) key finding algorithm in order to have a reference model for performance comparison. The simplistic nature of the KS key finding algorithm allows for a quick comparison and evaluation on the benefits of having a more detailed representation (such as the spiral representation) for harmony analysis.

The Krumhansl-Schmuckler algorithm uses the frequency of the notes inside a time window in one scale (12-note) representation. These counts are normalized in the same way the CoE algorithm does, leading to a 12-note distribution P_w that is compared to major and minor key profiles ($\mathbf{P_k}$) built from experimental cognitive data [2]. The selected key profile for the set of notes is the one that maximizes the correlation value with respect to the empirical 12-note distribution.

$$\underset{k \in K_{KS}}{\arg\max}(\text{Cor}(P_w, P_k)), \tag{S16}$$

where K_{KS} is the set of major and minor keys profiles of the KS algorithm.

We compared the results obtained with our implementation of the center of effect and the Krumhansl-Schmuckler algorithms with a fixed window size equivalent to a tenth of the piece length in quarterbeats. Figs S6 and S7 show the accuracy results for both CoE and KS key finding algorithms compared to professional hand annotated notes. Both key finding algorithms perform relatively well with the Mozart pieces, with CoE noticeably outperforming KS. This difference is larger with the selected pieces from Beethoven, evidence for the benefits of using the modified CoE key finding algorithm compared to Krumhansl-Schmuckler.

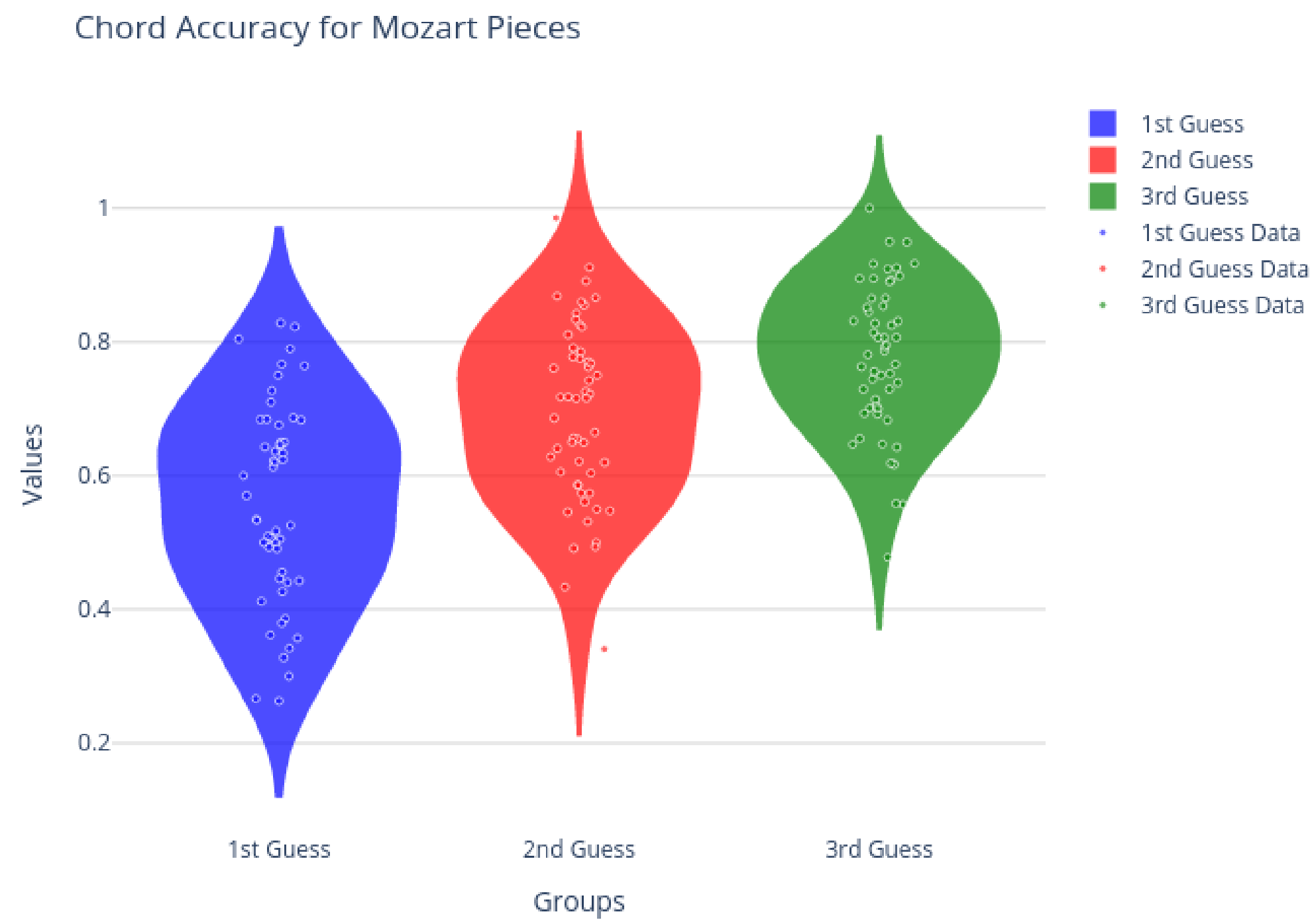

Fig S4. Chord accuracy for Mozart's piano sonatas. A correct guess is considered when the chord given by the CoE is the one in the annotated tables. Second and third guesses mean the second or third chord in the CoE corresponds to the one in the annotated pieces.

References

1. DCMLab/dcml_corpora; 2026. https://github.com/DCMLab/dcml_corpora. Available from: `https://github.com/DCMLab/dcml_corpora`.

2. Temperley D. What's Key for Key? The Krumhansl-Schmuckler Key-Finding Algorithm Reconsidered. Music Perception. 1999;17(1):65–100. doi:10.2307/40285812.

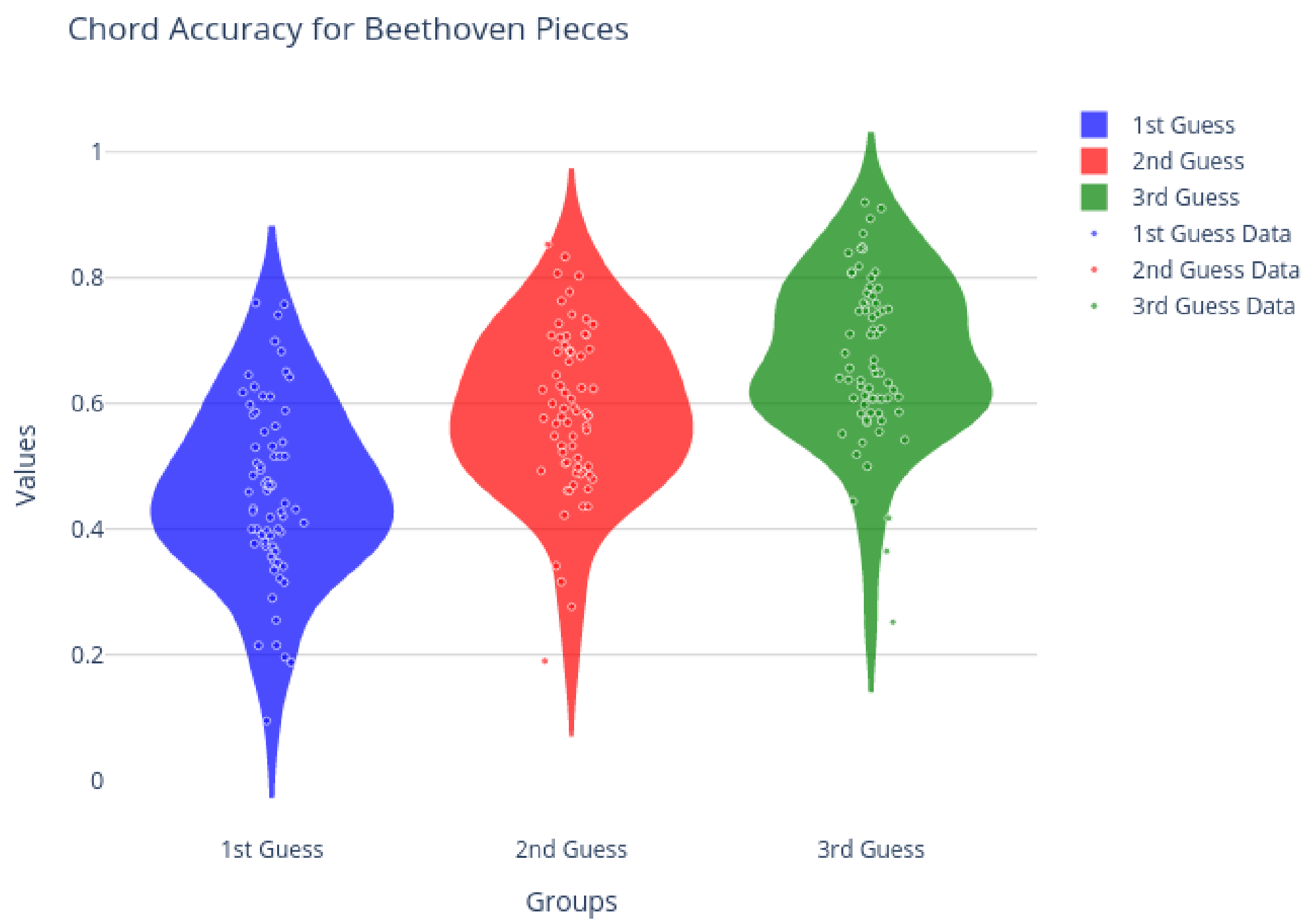

Fig S5. Chord accuracy for Beethoven's string quartets. A correct guess is considered when the chord given by the CoE is the one in the annotated tables. Second and third guesses mean the second or third chord in the CoE corresponds to the one in the annotated pieces.

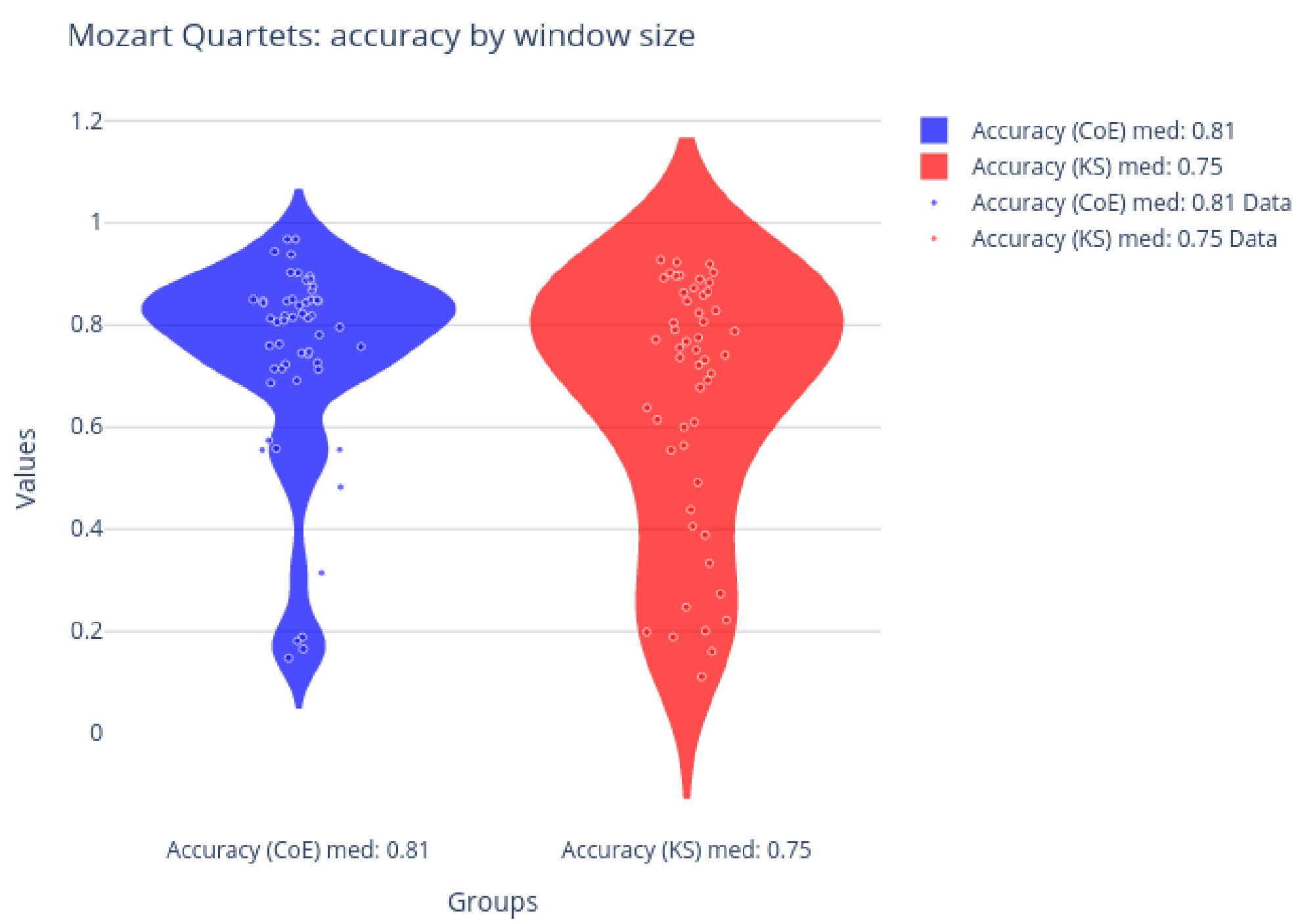

Fig S6. Key accuracy for Mozart's piano sonatas computed with (left, blue) the CoE algorithm and (right, red) the Krumhansl-Schmuckler (KS) key finding algorithm.

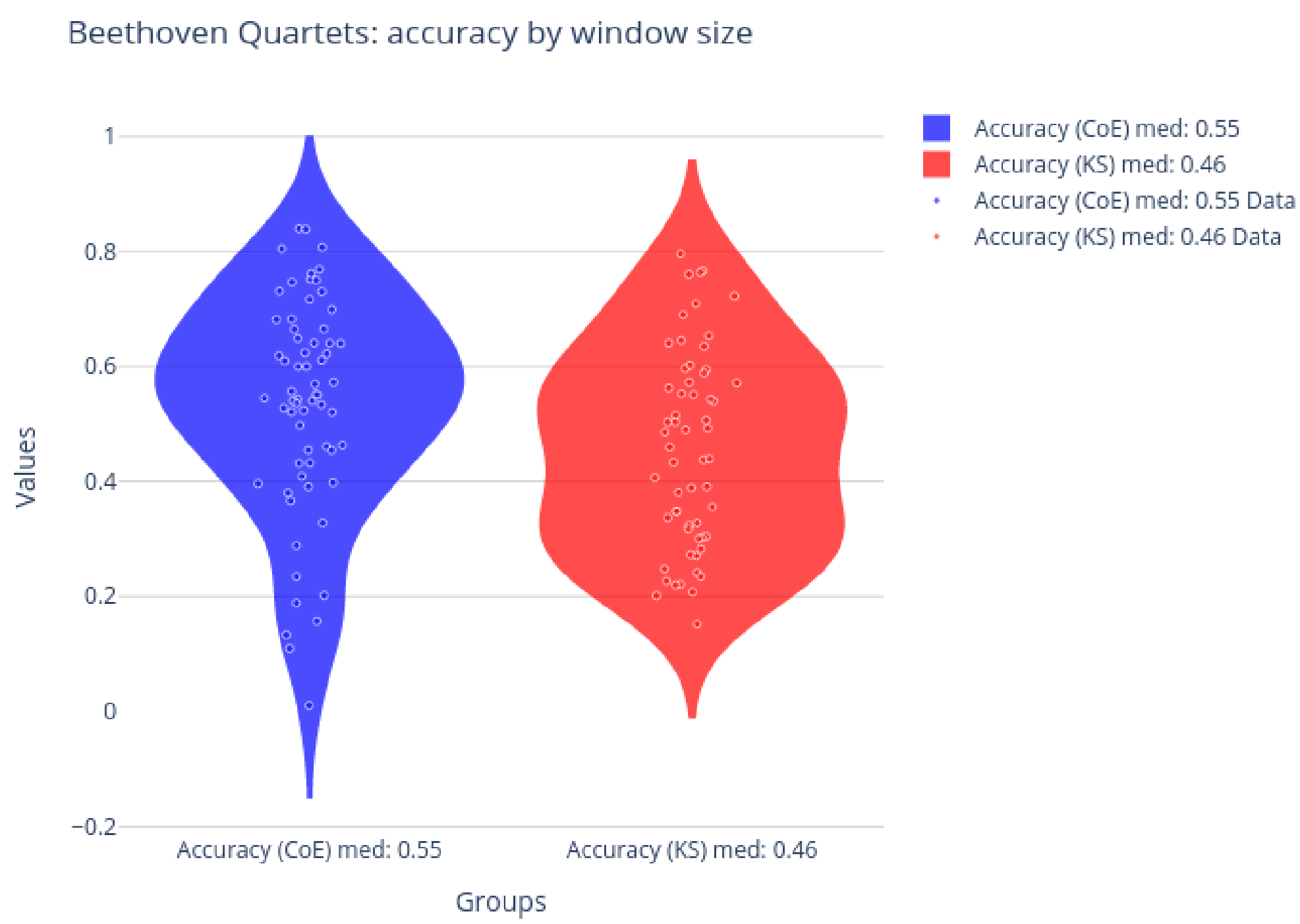

Fig S7. Key accuracy for Beethoven's string quartets computed with (left, blue) the CoE algorithm and (right, red) the Krumhansl-Schmuckler (KS) key finding algorithm.
