# Supplementary material for: Quantifying the evolution of harmony and novelty in western classical music
Source: PLoS One. 2026 Aug 20;21(8):e0356548. doi: 10.1371/journal.pone.0356548 (PMC13492798; doi:10.1371/journal.pone.0356548)
Supplement: Supplementary file 4 [file pone.0356548.s004.pdf]

## S4 Local keys to modulation in functional harmony

We construct a sequence of roman numerals from a sequence of keys with the mapping $f : K \rightarrow H$ between the set of keys $K = \{$C, G, D, A, E, B/Cb, Gb/F#, Db/C#, Ab, Eb, Bb, F, c, g, d, a, e, b, f#, c#, g#/ab, eb/d#, bb, f$\}$ and roman numerals $H = \{$I, I#/IIb, II, II#/IIIb, III, IV, IV#/Vb, V, V#/VIb, VI, VI#/VIIb, VII, i, i#/iib, ii, ii#/iiib, iii, iv, iv#/vb, v, v#/vib, vi, vi#/viib, vii$\}$, using the same musical notation as in [1], where the key of reference (I) is the mode value for the distribution of keys in the piece or key sequence $\xi$: $K_I = \text{mode}(\xi)$.

This mapping does not take into account the move to a different *center of harmony*, as a hand annotated functional harmony analysis would do.

## References

1. Neuwirth M, Harasim D, Moss FC, Rohrmeier M. The Annotated Beethoven Corpus (ABC): A Dataset of Harmonic Analyses of All Beethoven String Quartets. Frontiers in Digital Humanities. 2018;5(July):1–5. doi:10.3389/fdigh.2018.00016.
