# Supplementary material for: Quantifying the evolution of harmony and novelty in western classical music
Source: PLoS One. 2026 Aug 20;21(8):e0356548. doi: 10.1371/journal.pone.0356548 (PMC13492798; doi:10.1371/journal.pone.0356548)
Supplement: Supplementary file 5 [file pone.0356548.s005.pdf]

S5 Kullback-Leibler divergence rate

To compute the Kullback-Leibler divergence between the two models (P for the piece and Q for the previous pieces) we can use the definition for the Entropy rate of a Markov Process in terms of its transition matrix P given by:

$$H_R(P) = -\sum_i \mu_i \sum_j p(j \mid i) \log p(j \mid i) \tag{S17}$$

where $\boldsymbol{\mu}$ is the asymptotic distribution of states in P and $p(j \mid i)$ are the probabilities for the transitions $i \to j$. From the relation of Entropy with Kullback-Leibler divergence, we know that:

$$D_{KL}(P||Q) = H(P,Q) - H(P) \tag{S18}$$

where $H(P,Q)$ is the cross-entropy of distributions P and Q, defined as $H(P,Q) = -\sum_{x\in\mathcal{X}} P(x) \log Q(x)$; for notational consistency with the β-selection analysis we also write $\mathrm{CE}(P,Q) \equiv H(P,Q)$. Substituting the probability distributions with the transition matrices in terms of the Entropy Rate we obtain:

$$\begin{aligned} D_{KL_R}(P\|Q) &= H_R(P,Q) - H_R(P) \\ &= -\sum_i \mu_i \sum_j p(j \mid i) \log q(j \mid i) - \sum_i \mu_i \sum_j p(j \mid i) \log p(j \mid i) \\ &= \sum_i \mu_i \sum_j p(j \mid i) \log\left(\frac{p(j \mid i)}{q(j \mid i)}\right), \end{aligned} \tag{S19}$$

substituting the values for the entries of the matrices P and Q, we obtain:

$$D_{KL_R}(P\|Q_\beta) = \sum_i \mu_i \sum_j p(j \mid i) \cdot \log\left(\frac{p(j \mid i)}{q_\beta(j \mid i)}\right), \tag{S20}$$

where the entries $p(j \mid i)$ are defined in Eq (**??**) and the smoothed historical entries $q_\beta(j \mid i)$ are computed as:

$$q_\beta(j \mid i) = \frac{m_{ij} + \beta}{m_i + |\mathcal{A}|\,\beta}, \tag{S21}$$

where m_{ij} is the number of transitions from key i to key j in the historical pool, $m_i = \sum_j m_{ij}$ is the total number of transitions out of key i, and $|\mathcal{A}| = 24$ is the number of possible successor keys. Eq (S21) adds the same small pseudocount β to every possible transition before normalizing, which is the posterior predictive mean of a multinomial under a symmetric Dirichlet(β) prior. The pseudocount keeps transitions absent from the pool from receiving zero probability. A single β is selected for the whole corpus, as described in the smoothing-parameter sensitivity analysis (Appendix S6). The quantity $D_{KL_R}(P\|Q_\beta)$ is then the information per time step needed to reproduce the process distribution P using the model Q.

Although the KL divergence rate (**??**) is weighted by each piece's stationary distribution μ_i and therefore, in principle, should not depend on the sequence length N (the number of transition pairs), a finite-sample estimate can still carry a small positive floor. Empirically the raw and corrected values track each other closely and show no systematic dependence on N (Fig. S9). We therefore report the following length correction not as an adjustment to the reported trend but as a complementary statistical test that confirms the length independence of the measure, followed by a test for the significance of the temporal trend.

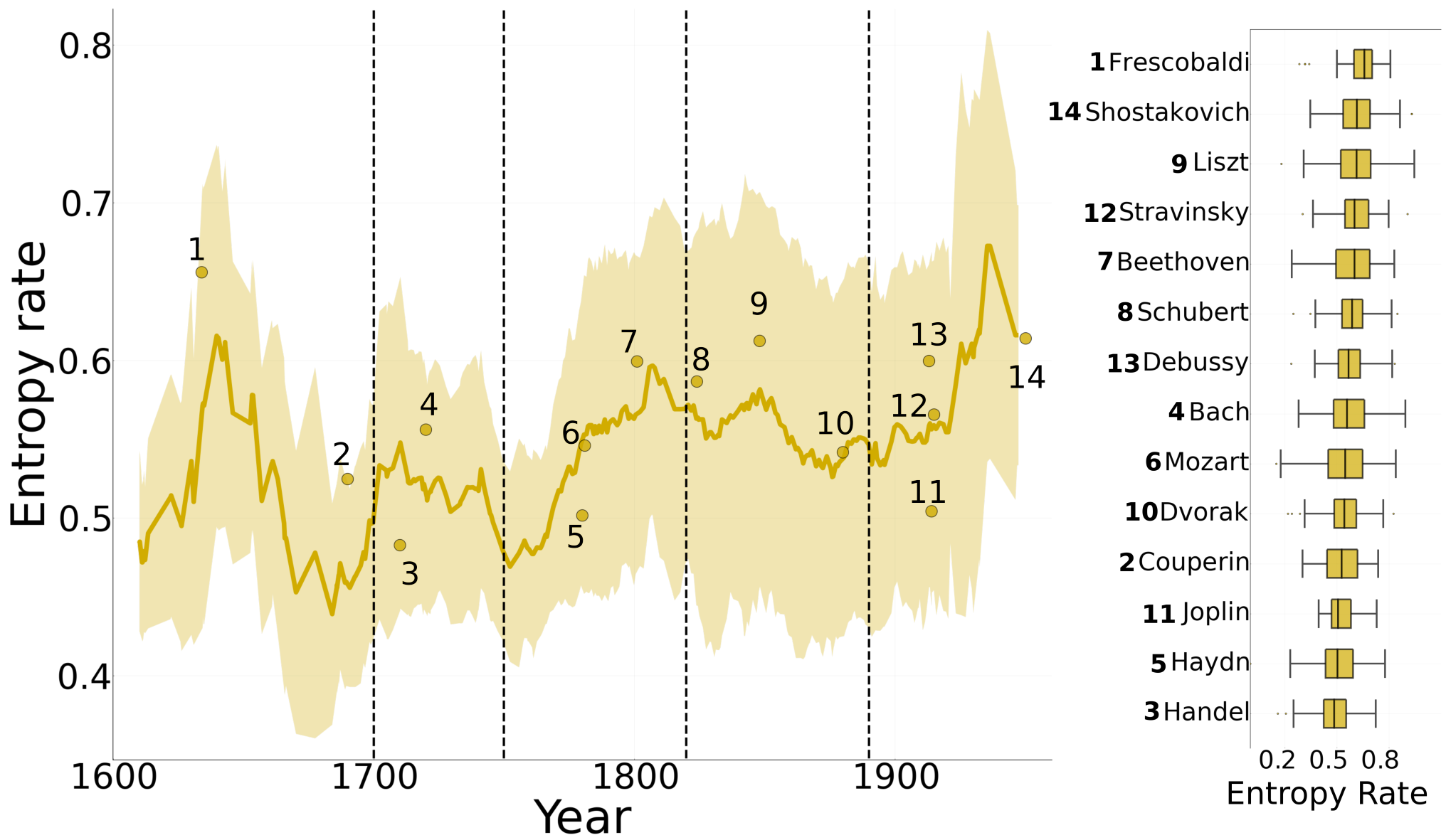

Fig S8. Entropy rate. Entropy rate of key-transition Markov processes over time.

S5.1 Excess novelty (floor correction)

The novelty-from-preceding-pool construction of [1] and [2] inherits a subtle finite-sample bias: even a piece that is an innovation-free draw from its pool has a strictly positive *estimated* divergence. By the Wilks / likelihood-ratio relationship between divergence and the χ^2 distribution [3, 4],

$$2N\, D_{KL}(\hat{P} \,\|\, Q) \xrightarrow{d} \chi^2_{\mathrm{df}}, \qquad \Longrightarrow \qquad \mathbb{E}\big[D_{KL_R} \mid \mathrm{null}\big] \approx \frac{\mathrm{df}}{2N}, \tag{S22}$$

where N is the number of transitions and $\mathrm{df} = \sum_i (\#\text{successors of } i - 1)$ summed over observed contexts. Because the metric (**??**) uses base-10 logarithms, the floor in those units carries a factor $1/\ln 10$. We define the **excess novelty** as the observed value minus this analytic floor,

$$D^{\mathrm{exc}}_{KL_R} \;=\; D_{KL_R} - \frac{\mathrm{df}}{2N \ln 10}. \tag{S23}$$

In principle, subtracting the floor will remove any artifact introduced by the piece length in the novelty value.

The length correction also leaves the long-term trend intact: the excess-novelty time series tracks the raw-novelty curve in both encodings (Fig S10), so the historical rise and fall in innovation is not an artifact of the per-piece length floor.

S5.2 Significance of the temporal trend

Each piece is scored against the *accumulated past*: a piece from year t has novelty $D_{KL_R}(P \,\|\, Q^{<t}_\beta)$ (Eq (**??**)), where $Q^{<t}_\beta$ pools the key transitions of all earlier pieces. This reference pool grows and changes over time by construction, and the number of pieces available in each year is uneven. Either feature could bend the median-novelty curve even if harmonic innovation were constant, so the global trend must be tested against a null that keeps this accumulation effect intact but removes any real link to the temporal order.

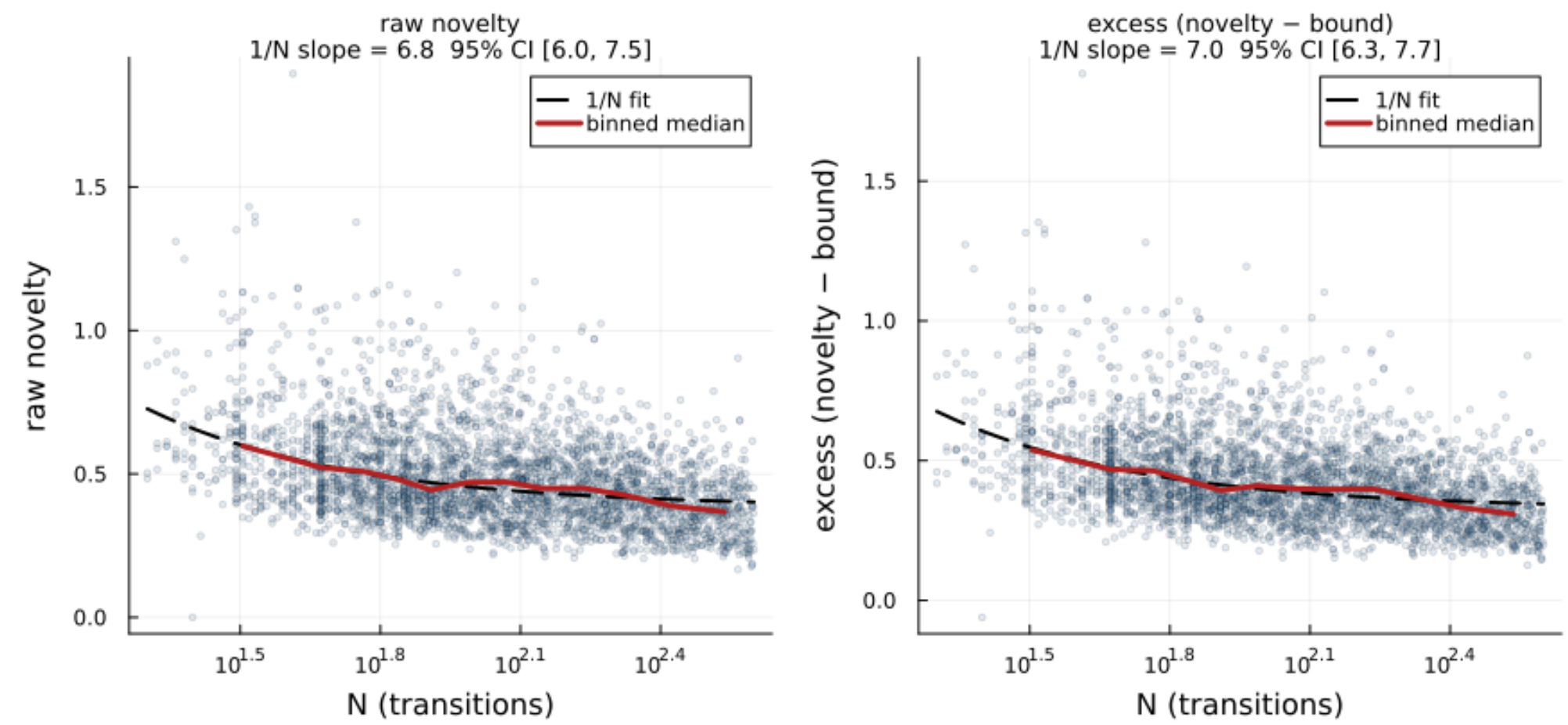

Fig S9. Novelty vs. number of pairs. Raw KL divergence rate and its analytic length correction (excess novelty) against the number of transition pairs N. Both are flat, confirming that the measure is effectively independent of piece length.

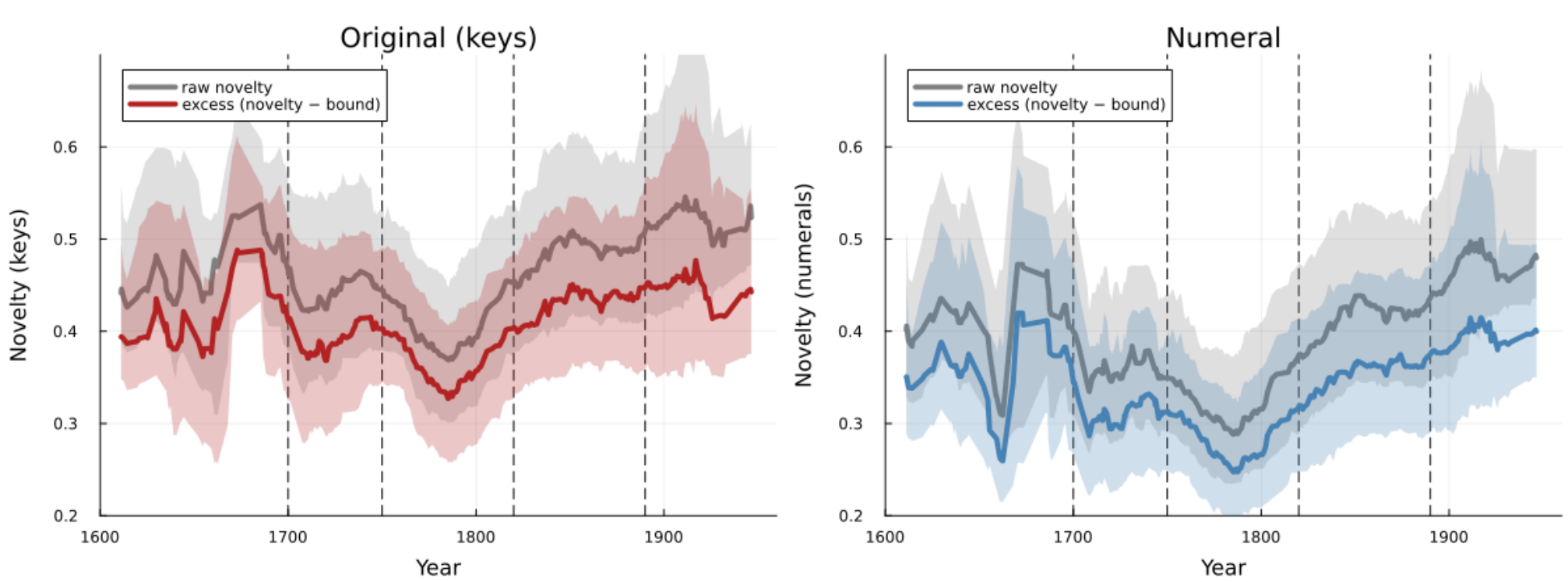

Fig S10. Novelty trend before and after the length-floor correction. Median raw novelty and excess novelty (observed minus the analytic floor, Eq (S23)) on a 20-year moving window, for the original local-key encoding and the transposed (numeral) encoding. The trend persists in the excess curve, showing that the historical signal reflects genuine innovation rather than the structural df$/2N$ floor.

We build that null by permuting the composition's years across pieces. A permutation leaves the set of pieces and their transition distributions P unchanged, and preserves the number of pieces per year—and with it the pool-growth schedule, the pool sizes, the smoothing β, and the window width—exactly; the only thing it destroys is the association between a piece's harmonic content and *when* it was written. For each permutation π we rebuild the cumulative prior-year pools under the shuffled chronology, rescore every piece, and re-smooth the result as the median novelty in a 20-year moving window (the same fashion as in the smoothing plots presented in this study). Repeating for $M = 500$ permutations gives, at each c, a pointwise 95% null band (the 2.5th–97.5th percentiles of the $\overline{D}^{\pi}(c)$). The band is the trend the accumulation-and-sampling machinery produces on its own: where the observed curve $\overline{D}(c)$ leaves it, the trend cannot be explained by pool growth, by the per-year sample sizes, or by the smoothing, and reflects genuine time dependence of innovation.

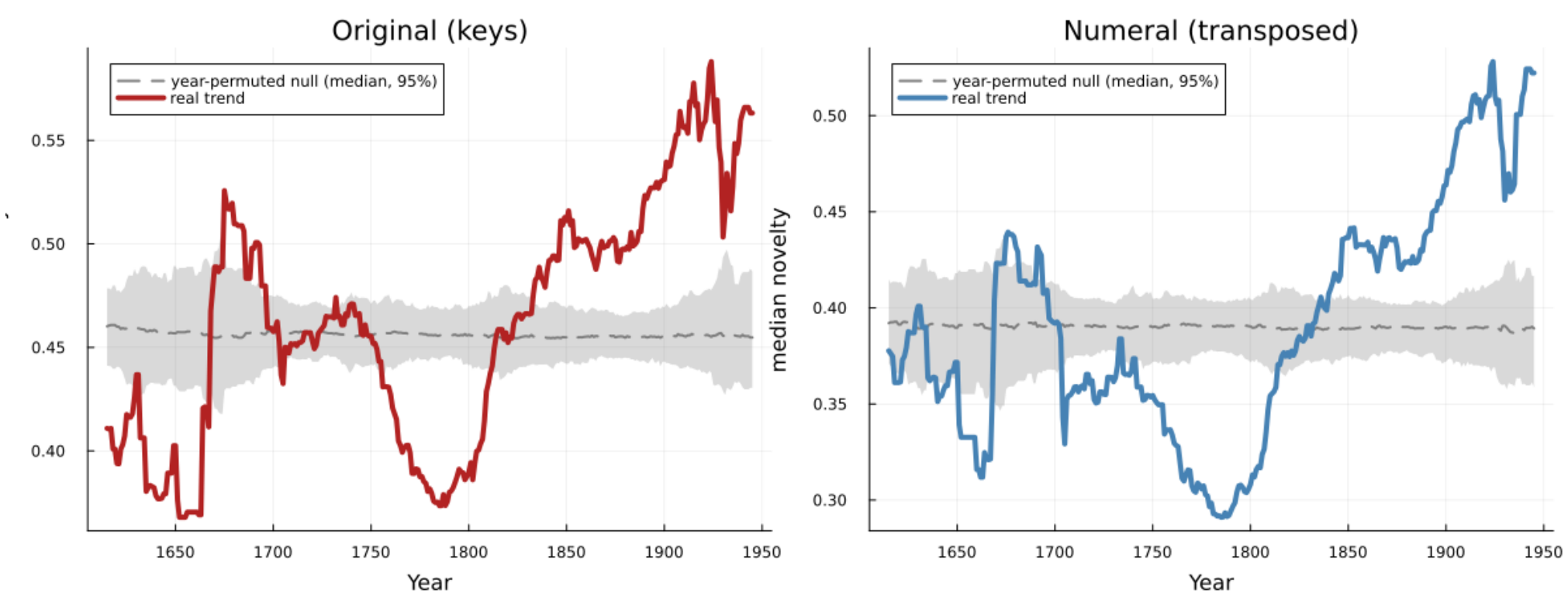

Fig S11. The novelty trend is significant against a year-permuted null. Median novelty on a 20-year moving window (solid) against the year-permutation null (dashed median with the 95% envelope), for the original local-key encoding and the transposed functional-harmony encoding. The null is built by permuting composition years across pieces, rebuilding the cumulative prior-year pools, and recomputing novelty ($M = 500$ permutations). The flat, narrow band confirms that the accumulation mechanism alone produces no trend; the observed curve leaves the band across the record.

The permuted curves form a flat band (Fig S11): under chronological exchangeability there is no global trend, only mild sampling noise that widens at the extremes where fewer pieces are available. The observed trend leaves the band over most of the record in both the original and transposed encodings, marking the Early-Classical decline and the Late-Classical-to-Modern rise as genuine temporal effects rather than artifacts of the measure. This time-resolved test complements the floor correction above: the floor removes the per-piece length bias, while the permutation null validates the shape and timing of the aggregate curve.

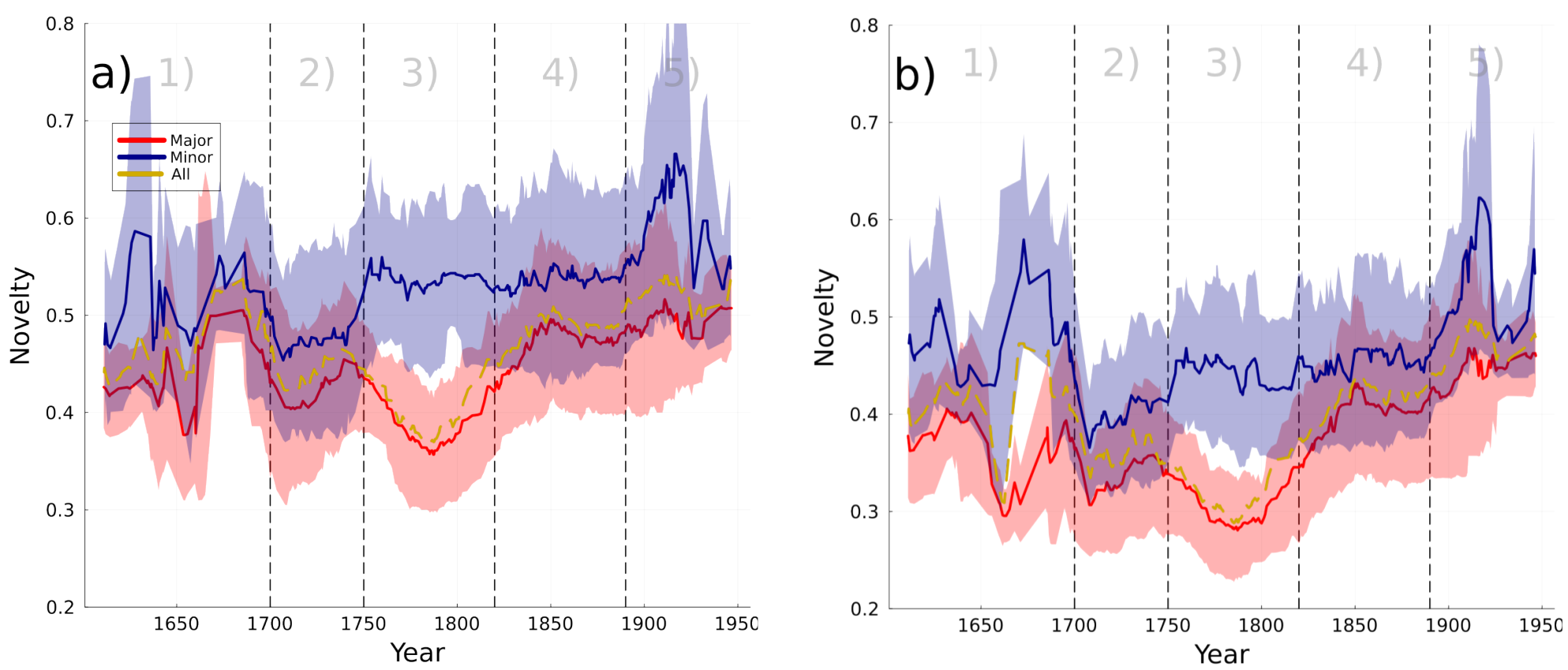

Fig S12. Novelty in major and minor keys. a) Original keys. Novelty values for scores represented in their original keys (without transposition), separating minor and major pieces. **b) Novelty scores after transposition to a common key.**

Year	Name	Composer	Novelty value	Sequence
1570	O Sacrum Convivium	F. Guerrero	0.2137	I-IV-I-ii-I-IV-I-I-V-IV-I-I-V-I-V-V-I-IV-V-I-V-IV-I-V-V-ii-I-IV-#VI/bVII-I-I-I-#VI/bVII-IV-I-I-I-I-V-V-I-I-vi-I-vi-vi-vi-I-vi-I-v-vi-ii-I-I-I-vi-V-ii-ii-ii-vi-I-v-IV-I-ii-I-I-IV-v-I-V-I
1696	String Sonata II Mov 4	D. Buxtehude	0.1969	I-I-IV-I-I-I-I-I-IV-I-I-vi-V-I-I-I-V-I-V-I-V-vi-V-I-vi-ii-I-vi-V-II-vi-V-V-V-I-V-V-V-V-V-I-V-V-iii-II-V-V-V-I-V-I-V-I-ii-I-IV-ii-v-IV-ii-I-V-ii-I-I-I-IV-I-I-I-I-I-IV-I-I-vi-V-I-I-I-I-I-I-iii-I-vi-vi-ii-vi-V-vi-V-V-V-V-I-I-I-ii-V-V-I-I-IV-V-vi-vi-I-vi-I-ii-V-V-I-I-ii-I-V-I-I-IV-I-ii-V-I-ii-iii-I-ii-I-ii-I-I-vi-III-vi-iii-vi-iii-V-I-V-ii-vi-VII-I-III-vi-V-I-I-IV-i-v-ii-III-IV-iii-vi-IV-IV-VII-III-vi-vi-iii-vi-vi-iii-vi-I-iii-I-I-V-V-vi-ii-vi-V-I-vi-vi-I-I-IV-IV-vi-II-iii-I-vi-VII-iii-vi-IV-ii-III-vi-ii-v-IV-IV-I-ii-vi-vi-vi-IV-vi-v-vi-IV-vi-vi-II-I-V-I-I-vi-I-V-V-I-IV-V-IV-I-IV-vi-v-v-II-V-I-V-vi-I-I-IV-I-I-I
1749	Royal Fireworks Suite Mov 4	G.F. Handel	0.1550	I-I-I-I-V-V-V-V-I-I-I-I-V-V-V-V-V-V-I-I-I-I-V-I-I-I-V-V-I-I-I-I-V-I-I-I
1798	7 Lander in D	Beethoven	0.1670	I-I-I-v-I-I-I-v-I-I-I-v-I-I-I-v-I-ii-ii-ii-V-I-I-v-I-ii-ii-ii-V-I-I-v-I-V-I-I-v-v-V-v-I-I-I-I-v-v-V-v-I-I-ii-ii-ii-V-ii-ii-ii-V-I-I-v-v-V-v-I-I-I-I-I-I-I-I-I-I-I-I-I-I-I-I-I-I-I-V-V-V-v-I-I-I-I-V-V-V-v-I-I-I-I-I-I-I-ii-I-i-I-v-I-I-I-ii-I-i-I-v-I-IV-IV-V-I-IV-V-I-IV-IV-V-I-IV-V-I-I-v-I-v-I-v-I-v-I-v-I-v-I-v-I-v-I-v-I-i-IV-i-IV-v-I-v-I-i-IV-i-IV-v-I-v-I-I-I-V-I-I-I-V-I-I-I-V-I-I-IV-I-v-I-IV-I-V-I-IV-I-v-I-IV-I-V-I-I-I-I-I-V-V-V-V-I-I-I-I-V-V-V-V-I-IV-IV-IV-V-I-I-V-I-IV-IV-IV-V-I-I-V-I-I-I-i-I-I-I-I-I-I-I-I-I-I-I-I-I-I-I-I-I-I-I-i-I-i-I-I-I-I-I-I-I-I-I-I-I-i

Year	Name	Composer	Novelty value	Sequence
1867	Les Mois Op. 74 Mov 4	Alkan	0.1888	V-I-I-I-I-I-i-I-I-I-I-I-I-I-i-I-I-I-I-I-I-I-I-I-I-I-I-I-I-I-I-I-I-I-I-I-I-I-I-I-ii-V-ii-V-ii-V-V-V-I-I-I-I-I-i-I-I-I-I-I-I-I-I-I-I-I-V-V-I

Table S1. Full key sequences for the lowest-novelty compositions (cf. 2).

Year	Name	Composer	Novelty value	Sequence
1570	Madrigals Book 4 #19	C. Gesualdo	1.72	I-IV-I-vi-V-IV-I-IV-ii-I-V-I-IV-V-V-I-vii-V-ii-ii-vi-II-vi-III-vi-I-V-vi-I-I-vi-V-ii-V-II-II-vi-III-vi-I-V-vi-I-I-vi-V-ii-I-V-I-V-I-I
1696	Mass for the parishes Mov 14	F. Couperin	1.8597	V-vi-I-I-IV-I-I-IV-i-i-i-v-IV-I-i-I-IV-#II/bIII-i-i-#VI/bVII-iv-#II/bIII-#V/bVI-#II/bIII-#VI/bVII-#V/bVI-#VI/bVII-iv-i-#VI/bVII-iv-i-v-v-I-i-i-V-i-v-IV-v-II-iv-iv-IV-I-v-iv-#VI/bVII-vi-#II/bIII-#VI/bVII-V-ii-VI-V-V-#i/bii-V-vi-ii-#V/bVI-i-VII-#IV/bV
1718	WTC I Prelude 14 in F# minor	J.S. Bach	2.1341	i-i-#VI/bVII-i-#VI/bVII-#II/bIII-#II/bIII-v-v-v-v-v-#VI/bVII-#IV/bV-iv-i-i-i-i-#II/bIII-iv-i-I-#II/bIII-ii-vii-#VI/bVII-V-II-i-i-I-i-i-v-i-iv-i-I-VII-i-i-v-i-i-iv-i-i-I-v-i-i-I-i-iv-i-VII-IV-#II/bIII-i-I-i-I
1817	Sonata Op. 147 Mov 1	F. Schubert	1.6387	I-I-I-V-I-I-I-I-IV-I-iv-#II/bIII-#V/bVI-V-V-I-I-I-V-I-I-V-iv-#vi/bvii-V-I-i-v-ii-#V/bVI-#II/bIII-#V/bVI-III-#I/bII-#i/bii-iv-VI-#I/bII-#VI/bVII-i-#V/bVI-#II/bIII-#V/bVI-#vi/bvii-#V/bVI-#vi/bvii-#V/bVI-#ii/biii-#V/bVI-#V/bVI-#II/bIII-I-I-I-V-I-I-I-I-IV-I-iv-#ii/biii-#V/bVI-V-V-I-IV-#VI/bVII-#II/bIII-#II/bIII-V-V-#II/bIII-#II/bIII-I-IV-i-i-iv-I-I
1861	Esquisses Op. 63 No. 7	Alkan	1.58	I-I-I-I-vi-#i/bii-v-#i/bii-III-III-III-#i/bii-III-#i/bii-#iv/bv-VI-#i/bii-#IV/bV-vii-#I/bII-#V/bVI-i-#II/bIII-#II/bIII-i-#V/bVI-V-i-i-I-i-i-i-i-I-I

Table S2. Full key sequences for the highest-novelty compositions (cf. Table 3).

References

1. Klingenstein S, Hitchcock T, DeDeo S. The civilizing process in London's Old Bailey. Proceedings of the National Academy of Sciences. 2014;111(26):9419–9424. doi:10.1073/pnas.1405984111.

2. Barron ATJ, Huang J, Spang RL, DeDeo S. Individuals, institutions, and innovation in the debates of the French Revolution. Proceedings of the National Academy of Sciences. 2018;115(18):4607–4612. doi:10.1073/pnas.1717729115.

3. Wilks SS. The large-sample distribution of the likelihood ratio for testing composite hypotheses. Annals of Mathematical Statistics. 1938;9(1):60–62. doi:10.1214/aoms/1177732360.

4. Kullback S. Information Theory and Statistics. Wiley; 1959.
