# Supplementary material for: Quantifying the evolution of harmony and novelty in western classical music
Source: PLoS One. 2026 Aug 20;21(8):e0356548. doi: 10.1371/journal.pone.0356548 (PMC13492798; doi:10.1371/journal.pone.0356548)
Supplement: Supplementary file 6 [file pone.0356548.s006.pdf]

S6 Parameter β selection and sensitivity analysis

The parameter β in (**??**) regularizes the *historical model* (the measuring instrument) not the piece being measured. We therefore fix a single corpus-wide β and score every piece against the same-calibrated history.

S6.1 Selecting $\beta^\star$ by held-out predictive cross-entropy

We select β by minimizing a held-out predictive loss, a standard cross-validatory / empirical-Bayes criterion [1, 2, 3]. The natural loss is the cross-entropy of each piece's transition stream under the smoothed pool,

$$\mathrm{CE}(P, Q_\beta) \;=\; -\sum_i \mu_i \sum_j p(j \mid i)\, \log q_\beta(j \mid i). \tag{S24}$$

We choose this objective because: 1. The year-cumulative pool is *already* an out-of-sample split at corpus scale: a piece is predicted only from previous years, so no train/test partition is needed (according to the prequential principle [4]). 2. Cross-entropy is a proper scoring rule [5], so minimizing it gives the *best-calibrated* Q. Moreover, by the identity

$$\mathrm{CE}(P, Q_\beta) \;=\; D_{KL}(P \,\|\, Q_\beta) + H(P), \tag{S25}$$

and because the piece's own entropy rate $H(P)$ does not depend on β, minimizing held-out cross-entropy over β is similar to minimizing the novelty (**??**) over β. The selected value is

$$\beta^\star = \arg\min_\beta \; \mathrm{median}_{\mathrm{pieces}} \; \mathrm{CE}(P, Q_\beta), \tag{S26}$$

the argmin of the cross-piece median curve over a logarithmically spaced grid of β (Fig. S13).

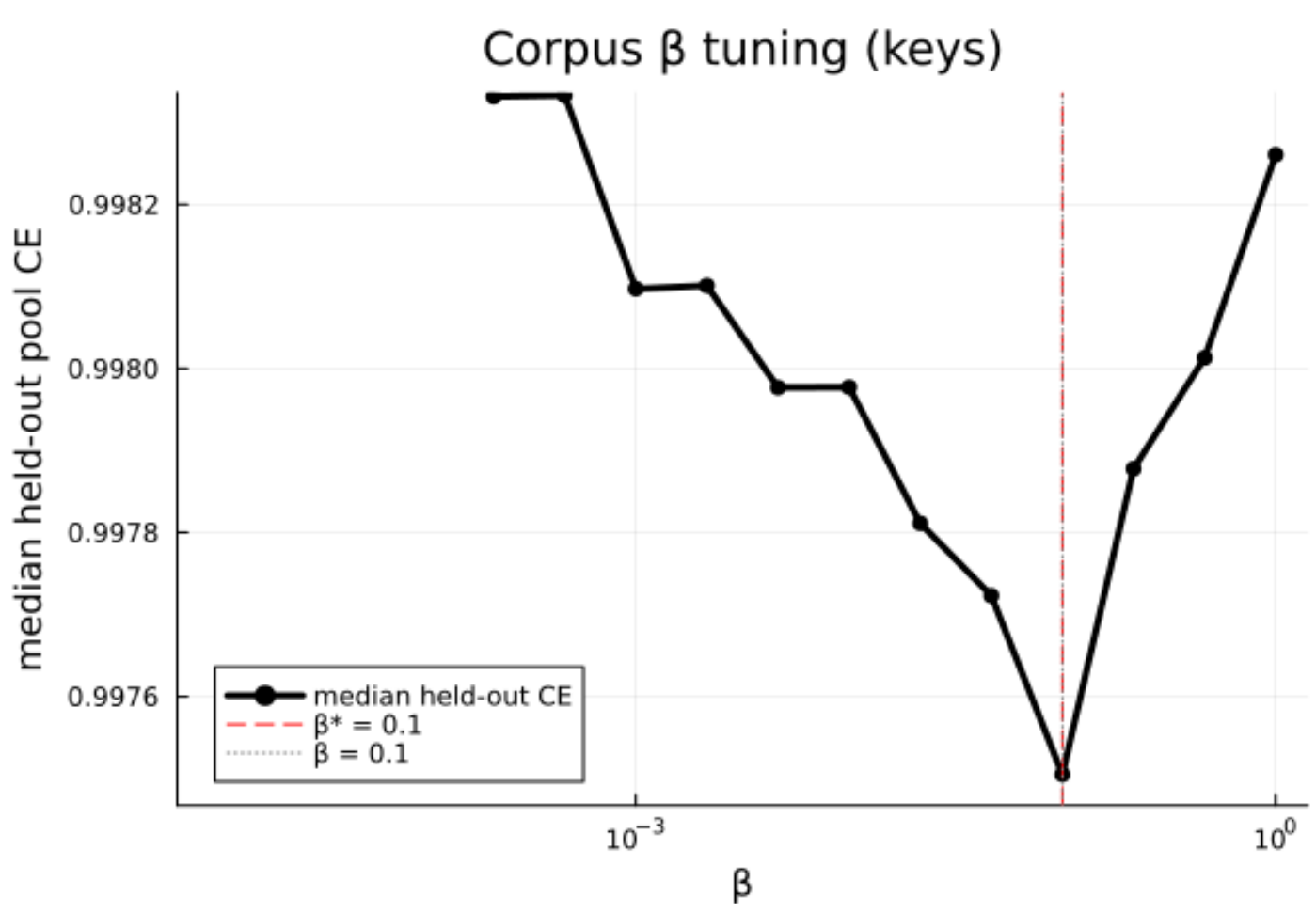

Fig S13. Corpus-wide selection of the smoothing parameter $\beta^\star$ by held-out predictive cross-entropy, shown for the absolute (key) encoding.

S6.2 Robustness of the β parameter

To test the robustness or stability of the smoothing parameter β we computed 3 different correlations: per piece metrics, per composer (median) and novelty value over time. We do a grid search over β, at each value, recompute the three quantities the study reports and compare them across β with the correlation matched to $\beta = 0.1$(Fig. S14):

- **Per-piece rankings**: Spearman rank correlation, which is invariant to the monotone rescaling that β induces.
- **Composer medians**: Spearman correlation across the medians, since the relevant content is the ordering.
- **Temporal trend curve**: The median of the whole innovation curve computed with different β values.

Figure S14 shows the results for the three metrics for different values of β

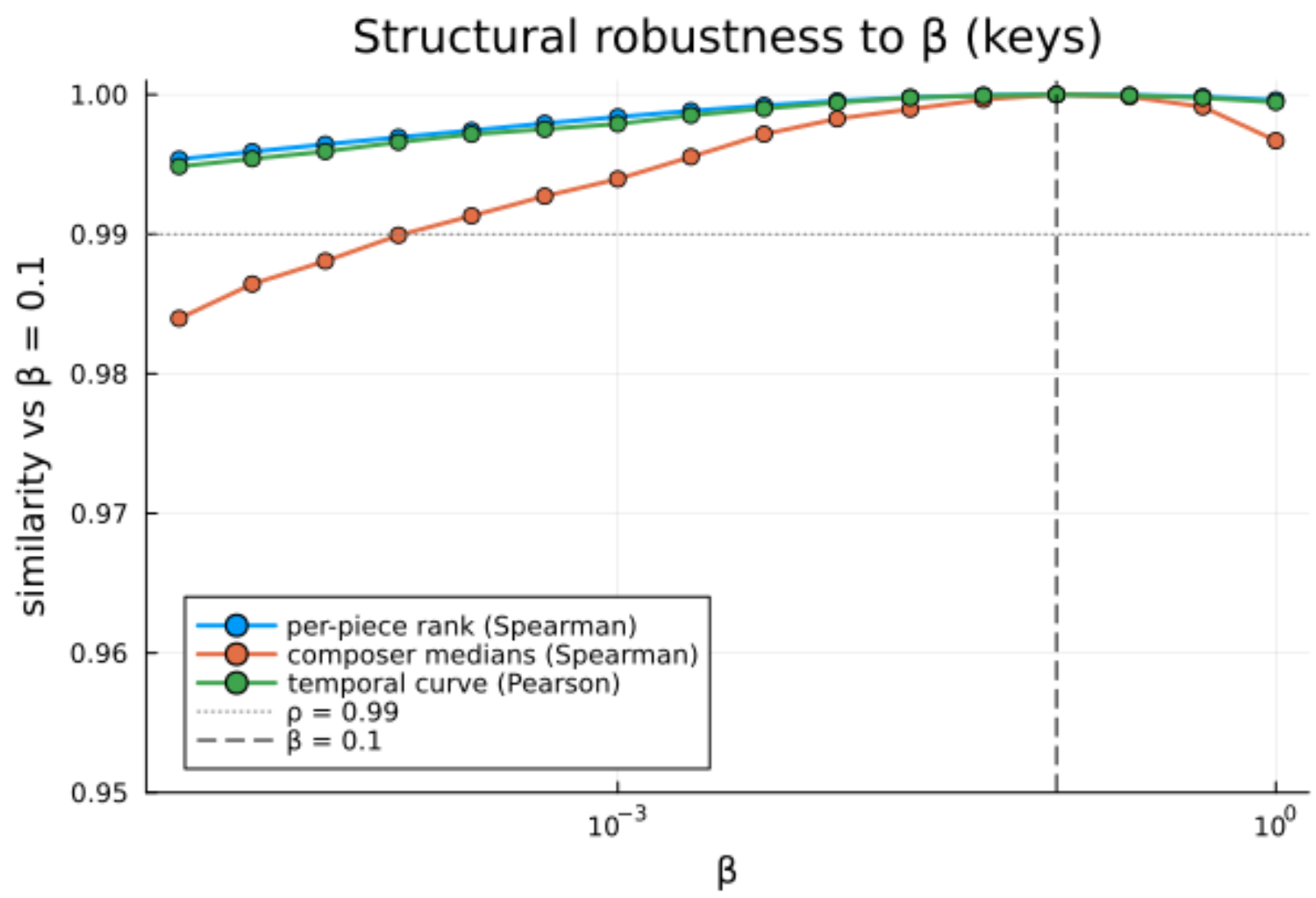

Fig S14. Robustness of the reported structures (rankings, composer medians, temporal trend) to the choice of β.

A complementary view (Fig. S15) shows which structure is most sensitive as β leaves the reference value, and confirms that the *shape* of the temporal trend survives the β extremes in both the original-key and the transposed (numeral) encodings.

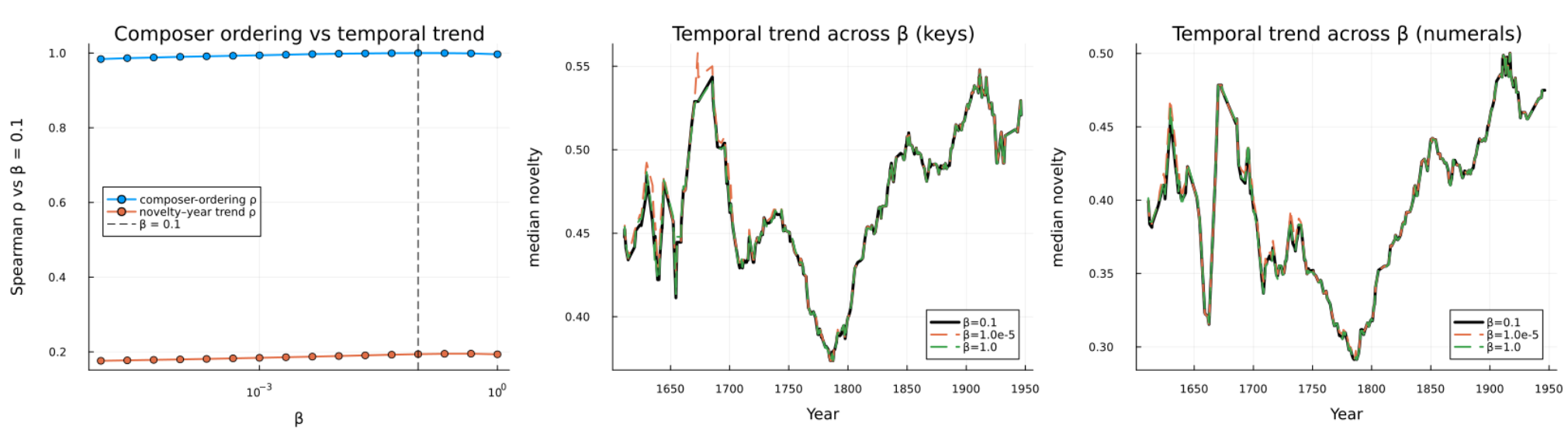

Fig S15. Robustness of the temporal trend to β. (a) Composer-ordering Spearman ρ and the novelty–year trend ρ as β varies, indicating which structure is most sensitive to the smoothing. (b) Median novelty over time for small, reference, and large β in the original-key encoding, and (c) the same for the transposed (numeral) encoding; the shape of the long-term trend is preserved across the β extremes in both representations.

References

1. Stone M. Cross-validatory choice and assessment of statistical predictions. Journal of the Royal Statistical Society B. 1974;36(2):111–133. doi:10.1111/j.2517-6161.1974.tb00994.x.

2. MacKay DJC, Peto LCB. A hierarchical Dirichlet language model. Natural Language Engineering. 1995;1(3):289–307.

3. Chen SF, Goodman J. An empirical study of smoothing techniques for language modeling. Computer Speech & Language. 1999;13(4):359–394. doi:10.1006/csla.1999.0128.

4. Dawid AP. Present position and potential developments: Some personal views. Statistical theory. The prequential approach. Journal of the Royal Statistical Society A. 1984;147(2):278–292. doi:10.2307/2981683.

5. Gneiting T, Raftery AE. Strictly proper scoring rules, prediction, and estimation. Journal of the American Statistical Association. 2007;102(477):359–378. doi:10.1198/016214506000001437.
