# Supplementary material for: Quantifying the evolution of harmony and novelty in western classical music
Source: PLoS One. 2026 Aug 20;21(8):e0356548. doi: 10.1371/journal.pone.0356548 (PMC13492798; doi:10.1371/journal.pone.0356548)
Supplement: Supplementary file 7 [file pone.0356548.s007.pdf]

S7 Markov-order tests

We implemented two different Markov tests to our constructed sequences: Bayesian Information Criterion, and a permutation test.

S7.1 Order selection by information criteria

For each piece we fit maximum-likelihood Markov models of order $k = 0, 1, 2, 3$ and score them with the Bayesian Information Criterion [1],

$$\mathrm{IC}(k) \;=\; -2\,\log \mathcal{L}(k) + p_{\mathrm{eff}}(k)\cdot \mathrm{penalty}, \qquad p_{\mathrm{eff}}(k) = |\mathcal{A}|^{k}(|\mathcal{A}| - 1), \tag{S27}$$

with the BIC penalty $\log N_{\mathrm{eff}}$. The order with the lowest criterion is selected. Because the parameter count of a k-th order model over 24 symbols grows as 24^k, the higher orders tend to be more penalized (number of combinations of symbols grows exponentially); this creates a preference for lower orders. To present this corpus-wide we plot, per order k, the median of the per-piece $\Delta\mathrm{BIC}(k) = \mathrm{BIC}(k) - \min_{k'} \mathrm{BIC}(k')$ (normalized per element so curves are comparable across lengths; Fig. S16): the curve rises steeply from $k = 1$, indicating that lower orders are preferred over higher ones. This result seems to suggest that by the BIC criteria, we don't gain much information with orders higher than zero, and could be due to the finitite size of our sequences.

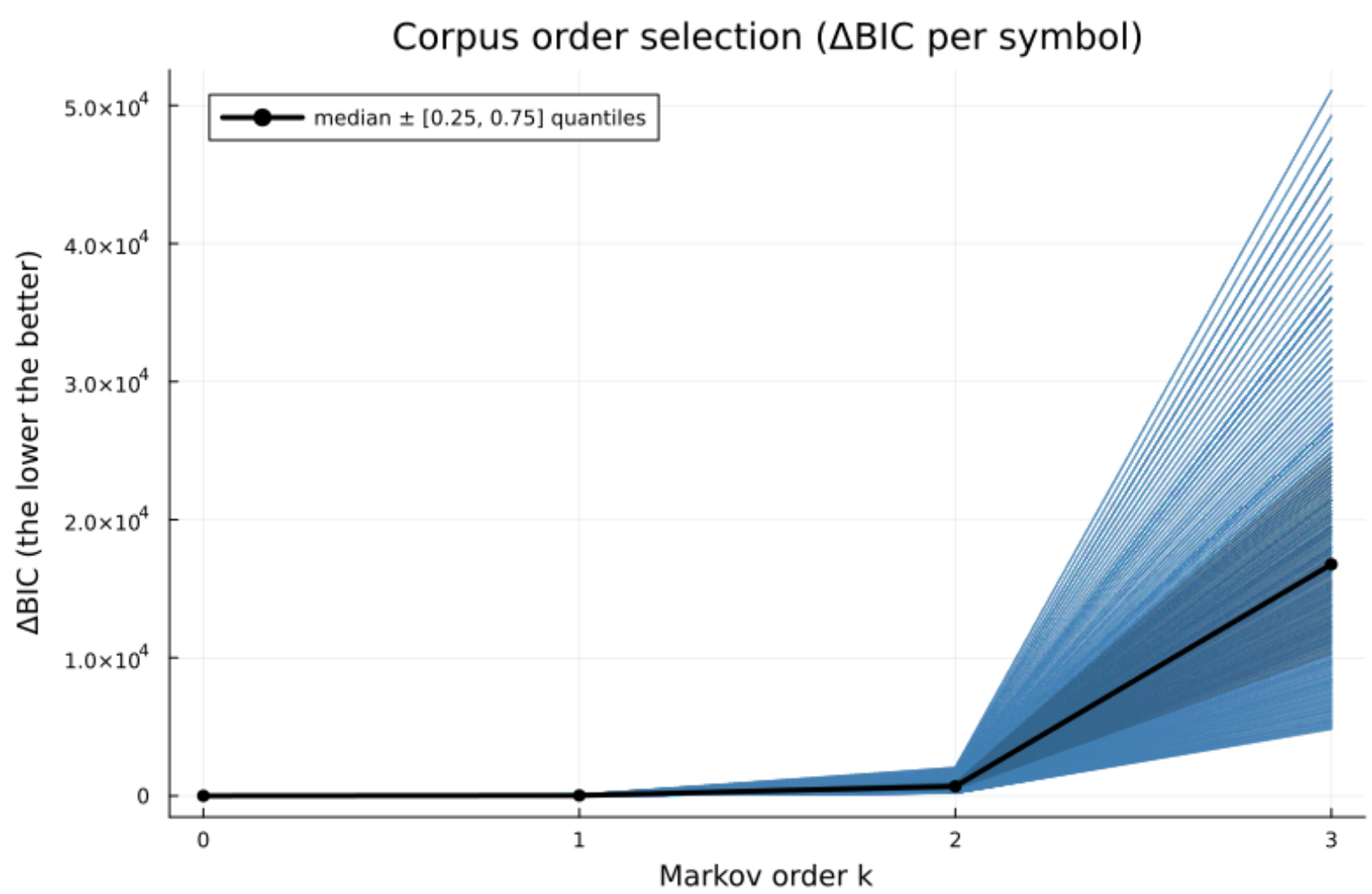

Fig S16. Corpus-wide order selection (ΔBIC per element).

S7.2 Permutation test for order-1 structure

The information criteria test above conflates "no structure" with "structure too weak for the model's parameters." We therefore test directly, whether the transition matrix built for our markov models carries information beyond the marginal distribution of keys. The statistic is the context-frequency-weighted Jensen–Shannon divergence [2] of each row of the transition matrix from the successor marginal—the Jensen–Shannon analogue of the mutual information $I(e_t; e_{t+1})$, which is zero in expectation when the sequence is order-0. Its null distribution is obtained by randomly shuffling the sequence (which preserves the marginal key distribution but destroys temporal order); we report an empirical rank p-value and a standardized effect size z (Fig. S17). In principle, this test isolates genuine sequential dependence from the static vocabulary distribution of keys.

Concretely, let $\hat{\pi}_i = n_i / \sum_k n_k$ be the empirical frequency of context i (the finite-sample estimate of the stationary weight μ_i of Eq. (**??**); cf. Eq. (**??**)), $\hat{p}(\cdot \mid i)$ the empirical transition rows, and $\bar{p}(j) = \sum_i \hat{\pi}_i \, \hat{p}(j \mid i)$ the marginal distribution of the following key. The statistic is the context-weighted (generalized) Jensen–Shannon divergence [2]

$$T(\hat{P}) \;=\; \sum_i \hat{\pi}_i \, D_{KL}\big(\hat{p}(\cdot \mid i) \,\|\, \bar{p}\big) \;=\; H(\bar{p}) - \sum_i \hat{\pi}_i \, H\big(\hat{p}(\cdot \mid i)\big), \tag{S28}$$

which coincides with the plug-in mutual information $\hat{I}(e_t; e_{t+1})$ and vanishes iff every row equals the marginal (order-0). We draw B surrogates $\tilde{\xi}^{(b)}$ by uniformly shuffling ξ (preserving key counts, destroying order) and recompute $T^{(b)} = T(\hat{P}^{(b)})$. The observed value is compared against the null sample $\{T^{(b)}\}$ by a null mean and standard deviation, a standardized score, and an empirical (one-sided) rank p-value,

$$\begin{aligned}
\bar{T} &= \frac{1}{B} \sum_{b=1}^{B} T^{(b)}, \qquad & s_T &= \left[\frac{1}{B-1} \sum_{b=1}^{B} \left(T^{(b)} - \bar{T} \right)^2 \right]^{1/2}, \\
z &= \frac{T - \bar{T}}{s_T}, \qquad & p &= \frac{1 + \#\{b : T^{(b)} \geq T\}}{B+1}.
\end{aligned} \tag{S29}$$

S7.3 Retention criterion

Pieces that pass *both* the significance and effect-size thresholds (empirical $p < 0.05$ and $z > 2$) are deemed to carry order-1 structure beyond the marginal and are retained for the innovation analysis; the remainder are treated as effectively order-0 for these purposes (Fig. S17). This mirrors the logic of complementary evidence used in studies of cultural-evolution of sequential data [3].

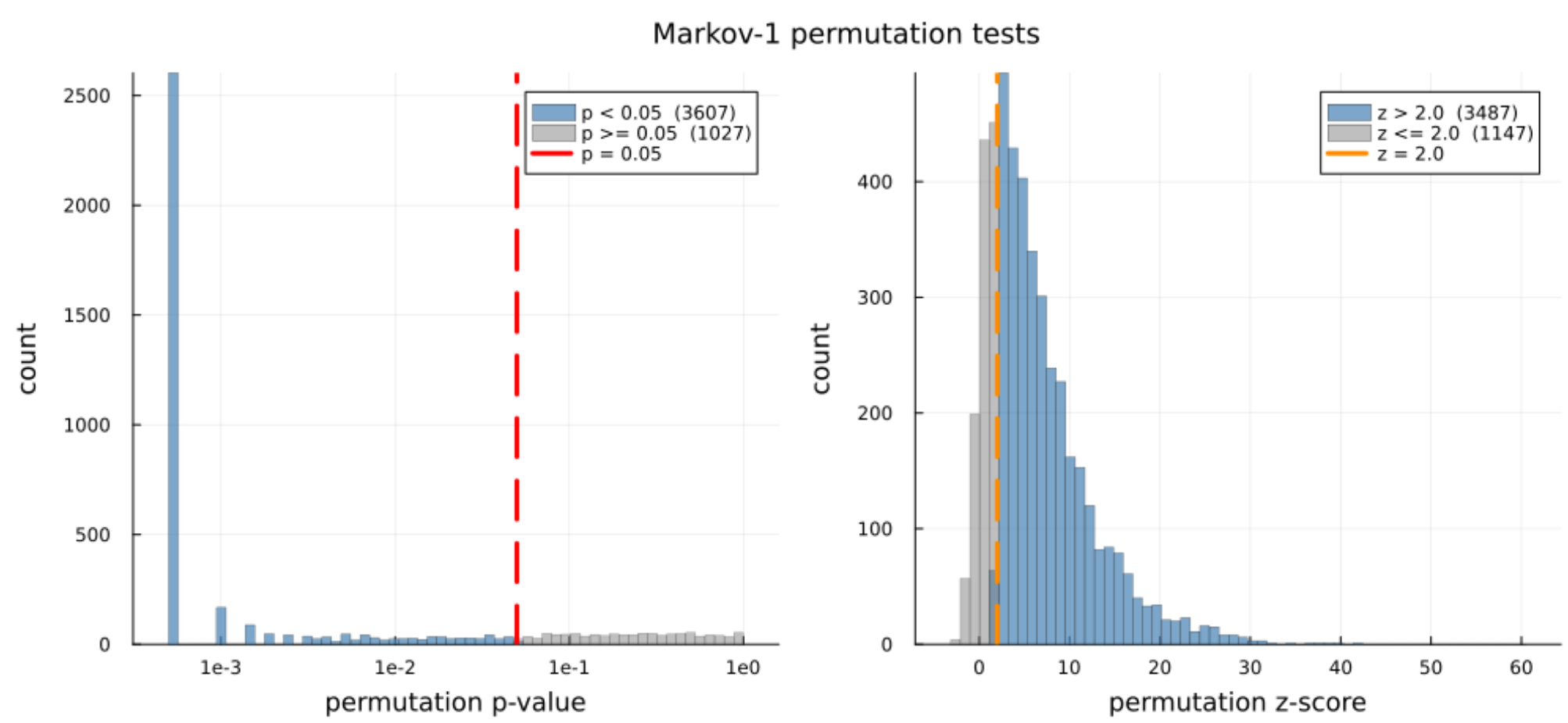

Fig S17. Distributions of the order-1 permutation-test statistics across the corpus, with the retention thresholds.

References

1. Schwarz G. Estimating the dimension of a model. Annals of Statistics. 1978;6(2):461–464. doi:10.1214/aos/1176344136.

2. Lin J. Divergence measures based on the Shannon entropy. IEEE Transactions on Information Theory. 1991;37(1):145–151. doi:10.1109/18.61115.

3. Mauch M, MacCallum RM, Levy M, Leroi AM. The evolution of popular music: USA 1960–2010. Royal Society Open Science. 2015;2(5):150081. doi:10.1098/rsos.150081.
