# Supplementary material for: Quantifying the evolution of harmony and novelty in western classical music
Source: PLoS One. 2026 Aug 20;21(8):e0356548. doi: 10.1371/journal.pone.0356548 (PMC13492798; doi:10.1371/journal.pone.0356548)
Supplement: Supplementary file 8 [file pone.0356548.s008.pdf]

S8 Composer harmonic similarity: pooling, support normalization, and embedding

Composer transition pools

For composer c we aggregate the first-order key transitions of all of that composer's retained pieces,

$$m_{ij}^{(c)} = \sum_{\xi \in c} n_{ij}^{\xi}, \qquad M^{(c)} = \sum_{i,j} m_{ij}^{(c)}, \tag{S30}$$

excluding sequence-boundary transitions, where n_{ij}^{ξ} are the per-piece counts of Eq (**??**). We summarize a composer by the joint pair distribution (**??**) rather than the row kernel $p^{(c)}(j \mid i)$, so that contexts contribute in proportion to how often they occur—the empirical analogue of the stationary weighting μ_i in Eq (**??**).

Symmetric similarity

We compare composers with the Jensen–Shannon divergence (**??**) [1, 2]. It is symmetric, bounded in $[0, 1]$ bit, and requires no smoothing parameter (the mixture $\bar{P}$ is supported wherever either composer is). Beyond satisfying the triangle inequality [2], $\sqrt{D_{\mathrm{JS}}}$ is of negative type: it embeds isometrically into a Hilbert space [3], properties required for the Euclidean embedding in the MDS method. The reported quantity is the similarity $S_{ab} = 1 - D_{\mathrm{JS}}$.

Support normalization by rarefaction

A composer pool (S30) is a finite sample, and D_{JS} estimated from finite samples is positively biased: two samples from the *same* distribution give $\widehat{D_{\mathrm{JS}}} > 0$, a noise floor that grows as the sample shrinks,

$$\mathbb{E}\big[\widehat{D_{\mathrm{JS}}} \mid \text{equal sources}\big] \approx \frac{c(|\mathrm{supp}|)}{M}, \tag{S31}$$

the same finite-sample effect behind the positive novelty floor (cf. the excess-novelty correction, Appendix S5). Since $M^{(c)}$ spans more than an order of magnitude across composers, a raw comparison would label small-corpus composers as spuriously distinctive; dividing by a count does not fix this, because the bias depends jointly on *both* pool sizes and on the number of populated transition types. We instead rarefy [4, 5]: with $M_{\min} = \min_c M^{(c)}$, we draw $M_{\min}$ transitions without replacement from each composer's pooled multiset, obtaining $\tilde{P}^{(c,r)}$, and average over R draws,

$$\widehat{D_{\mathrm{JS}}}^{\mathrm{rar}}_{ab} = \frac{1}{R} \sum_{r=1}^{R} D_{\mathrm{JS}}\big(\tilde{P}^{(a,r)}, \tilde{P}^{(b,r)}\big). \tag{S32}$$

Every pair is then estimated at equal support on both sides, so the values are comparable across corpus sizes; the residual floor common to all pairs does not change their ordering. The most- and least-similar composer pairs are read off the ranking of $\widehat{D_{\mathrm{JS}}}^{\mathrm{rar}}$ (equivalently of S_{ab}).

Visualization

The values $\{S_{ab}\}$ form a dense, continuous, symmetric matrix, which we display two ways. The seriated heatmap (Fig S18) plots every pairwise value with rows and columns reordered by the first embedding coordinate so that alike composers are adjacent and

block structure is visible [6, 7]. The metric MDS map (Fig S19) embeds composers in the plane by classical (Torgerson–Gower) MDS [8, 9] on the distances $d_{ab} = \sqrt{\widehat{D_{\mathrm{JS}}}^{\mathrm{rar}}_{ab}}$: with $D^{(2)} = [d^2_{ab}]$ and $J = I_n - \frac{1}{n}\mathbf{1}\mathbf{1}^\top$,

$$B = -\tfrac{1}{2}\, J\, D^{(2)}\, J = V \Lambda V^\top, \qquad X = V_k \Lambda_k^{1/2}, \tag{S33}$$

taking the $k = 2$ leading positive eigenpairs; the variance captured, $\sum_{\ell \le k} \lambda^+_\ell / \sum_\ell \lambda^+_\ell$, is reported on the figure so the 2-D distortion is explicit [10, 11]. We deliberately avoid a similarity network: it would require an arbitrary edge threshold and a force-directed layout that encodes topology rather than distance, asserting discrete relationships the data do not contain; the principled graph reduction (a relative-neighborhood graph or minimum spanning tree [12]) would still discard most pairwise information.

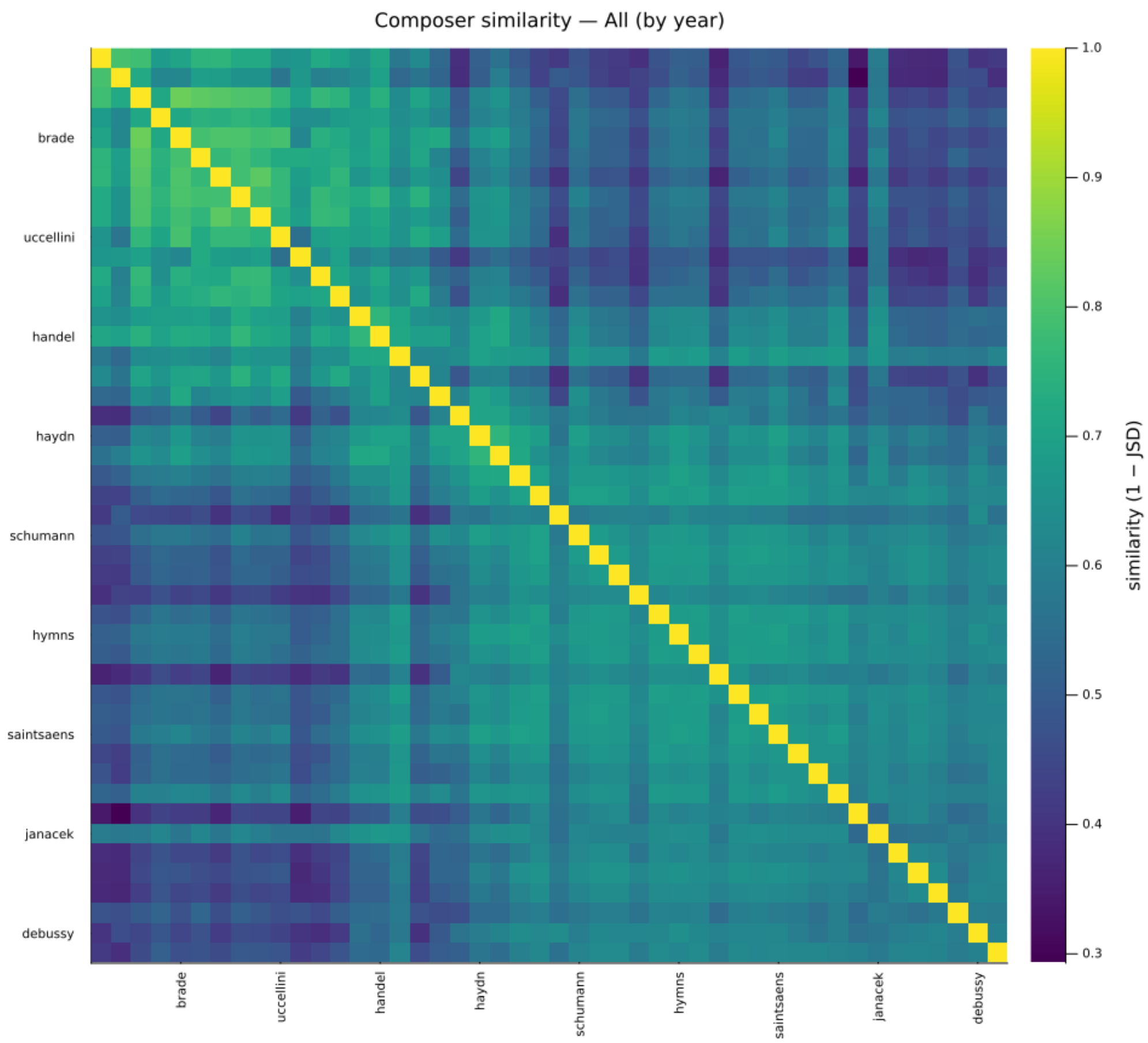

Fig S18. Composer harmonic similarity (all composers with ≥ 10 retained pieces): similarity matrix. Support-normalized similarity matrix $S_{ab} = 1 - \widehat{D_{\mathrm{JS}}}^{\mathrm{rar}}_{ab}$, seriated by the first MDS coordinate so that alike composers are adjacent.

Nearest-neighbor glyphs

The MDS map and seriated matrix summarize all pairwise similarities at once, but the two embedding axes capture only part of the variance, so a composer's exact closest neighbors are not always legible from position alone. As a complementary view we plot, for each central composer, its three most similar composers in the corpus, measured by

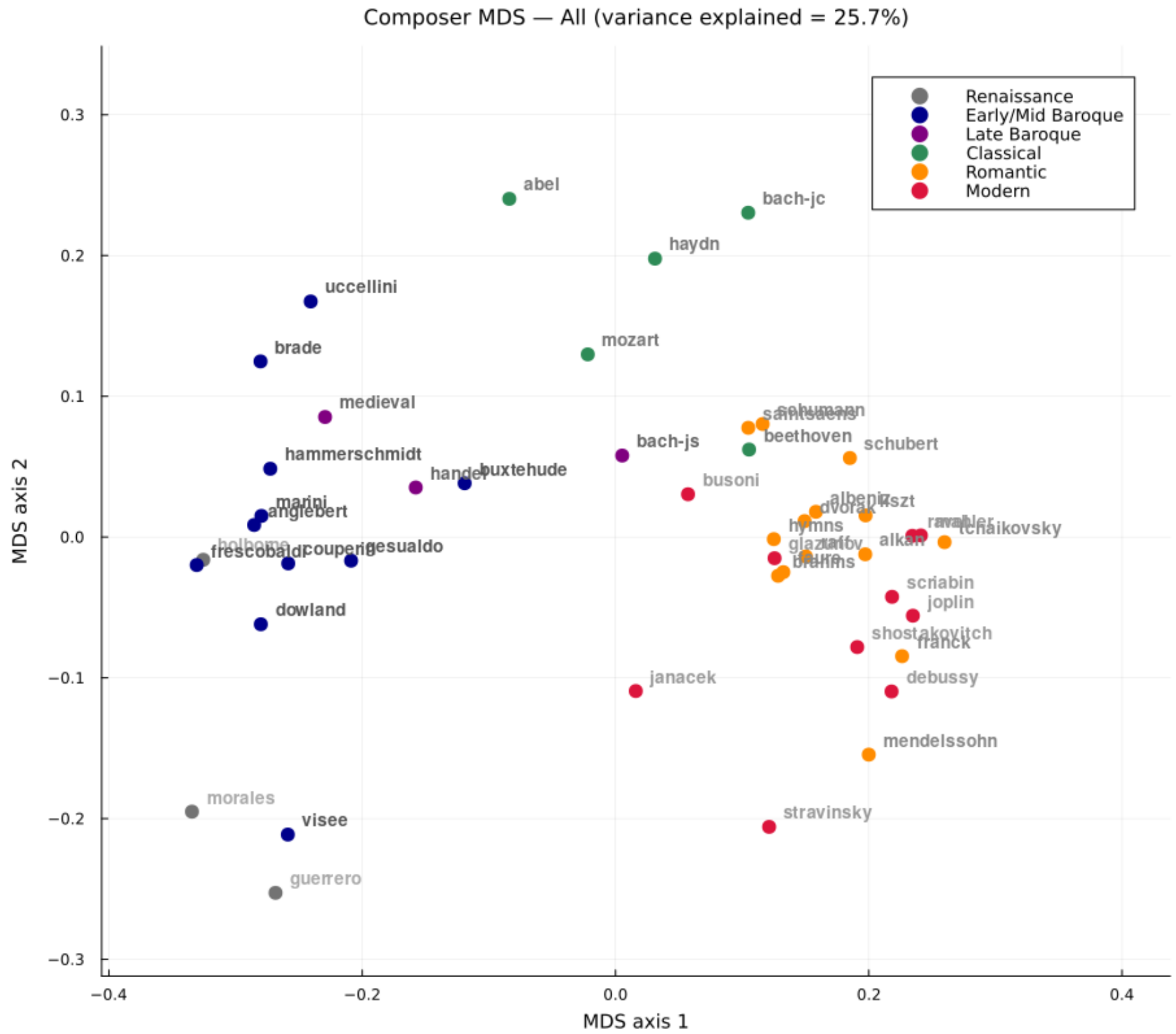

Fig S19. Composer harmonic similarity (all composers with ≥ 10 retained pieces): MDS map. Classical (Torgerson–Gower) MDS map of the distances $\sqrt{\widehat{D}_{\mathrm{JS}ab}^{\,\mathrm{rar}}}$; closer points are more similar in key-transition vocabulary.

the support-normalized similarity $1 - D_{\mathrm{JS}}$ on the full rarefied matrix (Fig S20). Bubble area encodes similarity, scaled to the global range so that bubbles are comparable across panels, and color encodes period; the central composer is the outlined hub. The label "Hymns" is excluded, being an aggregate of many anonymous settings rather than a single composer. These glyphs report exact nearest-neighbor relations in $\sqrt{D_{\mathrm{JS}}}$ space and therefore resolve orderings, such as the Romantic neighbors of Joplin noted in the main text, that the two-dimensional map cannot display.

References

1. Lin J. Divergence measures based on the Shannon entropy. IEEE Transactions on Information Theory. 1991;37(1):145–151. doi:10.1109/18.61115.

2. Endres DM, Schindelin JE. A new metric for probability distributions. IEEE Transactions on Information Theory. 2003;49(7):1858–1860. doi:10.1109/TIT.2003.813506.

3. Fuglede B, Topsøe F. Jensen–Shannon divergence and Hilbert space embedding. In: Proceedings of the International Symposium on Information Theory (ISIT 2004). IEEE; 2004. p. 31.

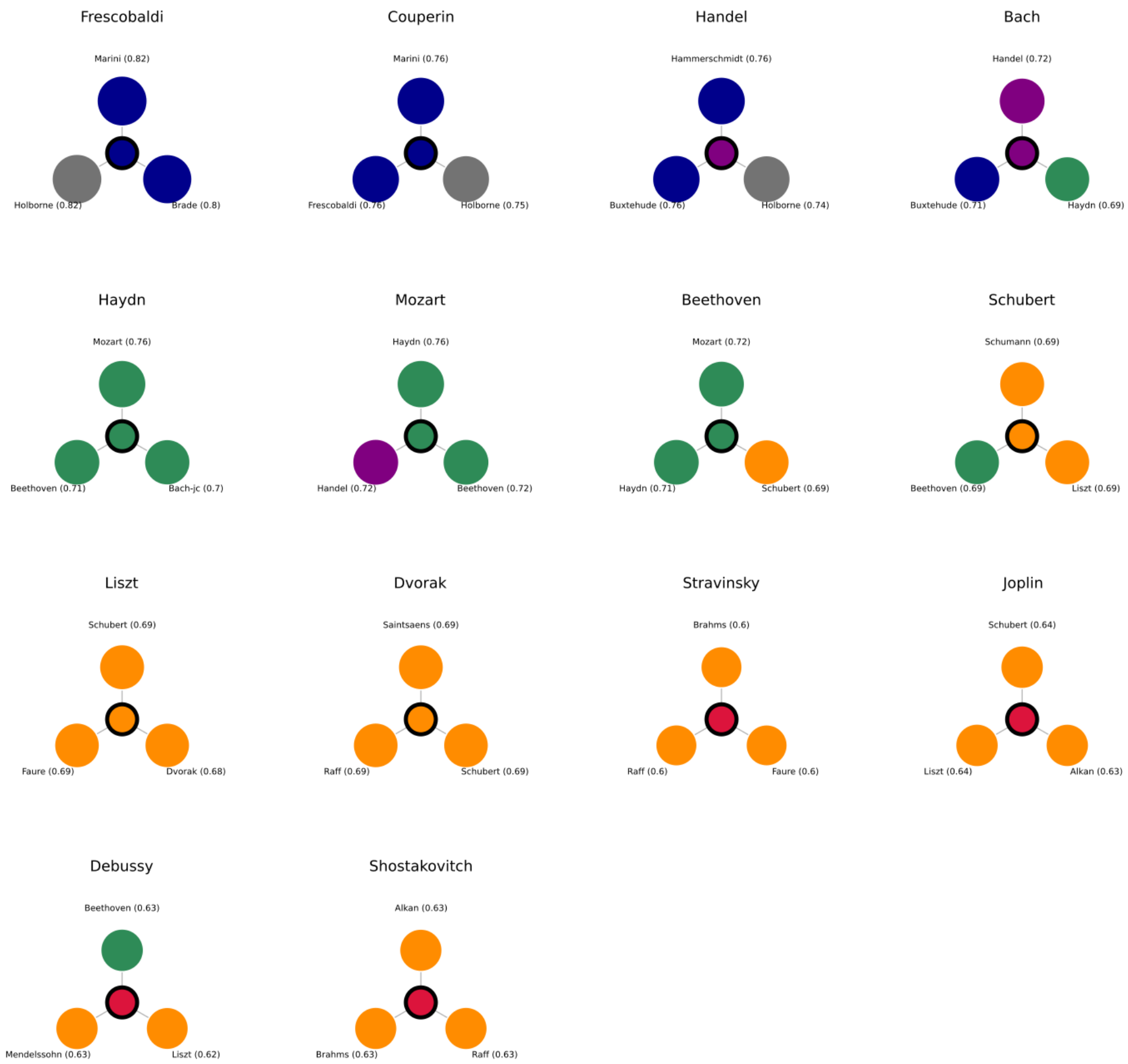

Fig S20. Top-three most similar composers per composer. Nearest-neighbor glyphs for the main-text composers: each hub (outlined) is surrounded by its three nearest composers by $1 - D_{\mathrm{JS}}$, with bubble area $\propto$ similarity and color denoting musical period.

4. Hurlbert SH. The nonconcept of species diversity: a critique and alternative parameters. Ecology. 1971;52(4):577–586. doi:10.2307/1934145.

5. Gotelli NJ, Colwell RK. Quantifying biodiversity: procedures and pitfalls in the measurement and comparison of species richness. Ecology Letters. 2001;4(4):379–391. doi:10.1046/j.1461-0248.2001.00230.x.

6. Wilkinson L, Friendly M. The history of the cluster heat map. The American Statistician. 2009;63(2):179–184. doi:10.1198/tas.2009.0033.

7. Hahsler M, Hornik K, Buchta C. Getting things in order: an introduction to the seriation package. Journal of Statistical Software. 2008;25(3):1–34. doi:10.18637/jss.v025.i03.

8. Torgerson WS. Multidimensional scaling: I. Theory and method. Psychometrika. 1952;17(4):401–419. doi:10.1007/BF02288916.

9. Gower JC. Some distance properties of latent root and vector methods used in multivariate analysis. Biometrika. 1966;53(3-4):325–338. doi:10.1093/biomet/53.3-4.325.

10. Kruskal JB. Multidimensional scaling by optimizing goodness of fit to a nonmetric hypothesis. Psychometrika. 1964;29(1):1–27. doi:10.1007/BF02289565.

11. Borg I, Groenen PJF. Modern Multidimensional Scaling: Theory and Applications. 2nd ed. New York: Springer; 2005.

12. Toussaint GT. The relative neighbourhood graph of a finite planar set. Pattern Recognition. 1980;12(4):261–268. doi:10.1016/0031-3203(80)90066-7.

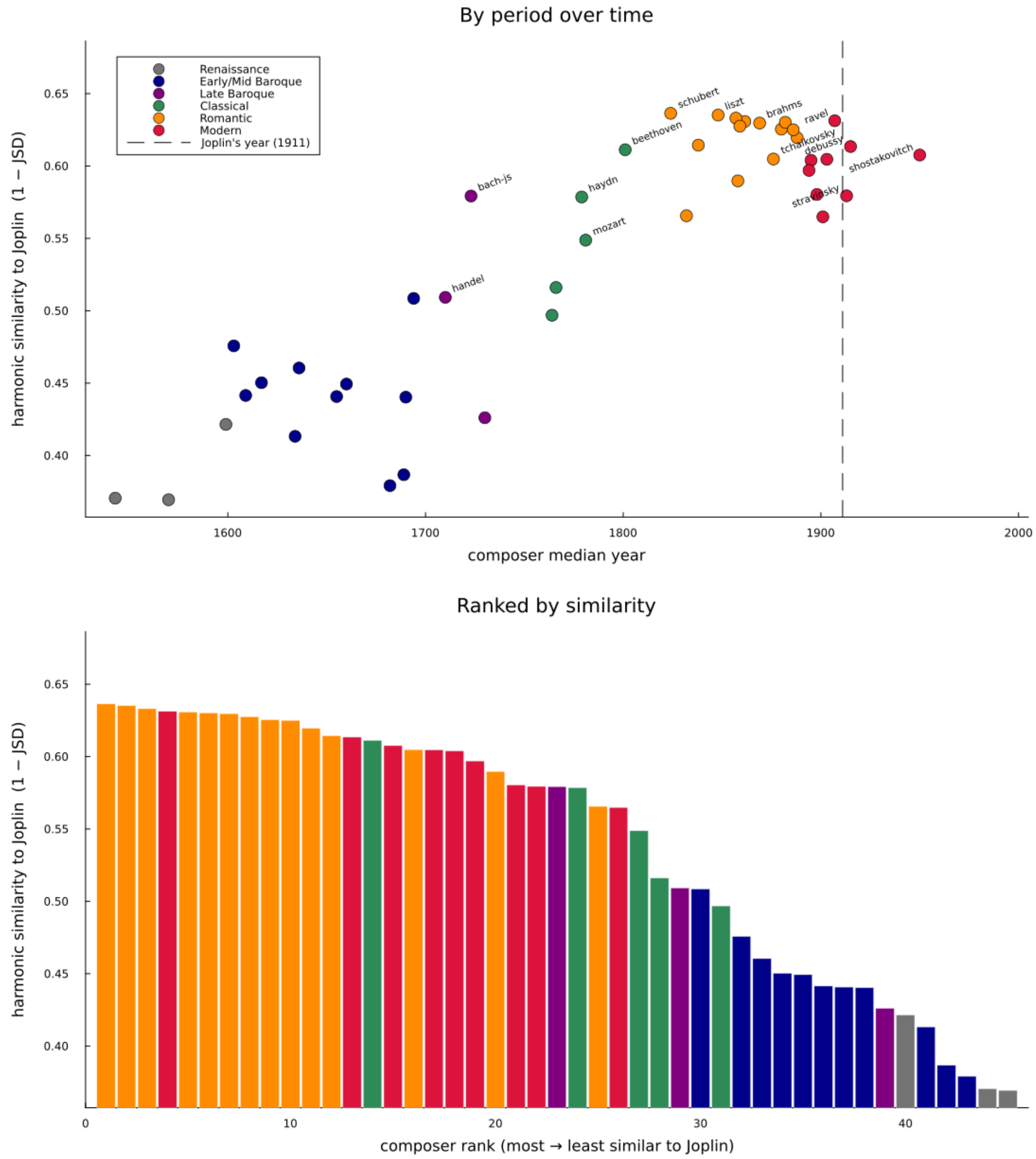

Fig S21. Harmonic similarity of every composer to Scott Joplin. Both panels use the support-normalized similarity $1 - D_{\mathrm{JS}}$ on the full rarefied matrix, with color denoting musical period. *Top:* similarity to Joplin plotted against each composer's median year; the dashed line marks Joplin's own year, and labeled points highlight canonical composers. Joplin's closest matches lie among earlier Classical and Romantic composers rather than his early-twentieth-century contemporaries. *Bottom:* the same similarities ranked from most to least similar to Joplin, with period-colored bars making the period ordering explicit.
