# Supplementary material for: Quantifying the evolution of harmony and novelty in western classical music
Source: PLoS One. 2026 Aug 20;21(8):e0356548. doi: 10.1371/journal.pone.0356548 (PMC13492798; doi:10.1371/journal.pone.0356548)
Supplement: Supplementary file 9 [file pone.0356548.s009.pdf]

S9 Key-uncertainty parameter λ: Boltzmann calibration and robustness

The key uncertainty of a bar (Eq (**??**)) is the Shannon entropy of a Boltzmann (softmax) distribution over the keys nearest the center of effect $\vec{c}_e$. Here we make the construction explicit, fix the inverse-temperature λ from the spiral-array geometry, bound the resulting entropy, and show that the reported quantities are insensitive both to the neighborhood cutoff N and to λ over a wide range.

The distance-to-probability map

For a bar with center of effect $\vec{c}_e$, the key-finder returns the Euclidean distances $d(T) = \|\vec{c}_e - T\|$ to the key positions T. Keys are weighted combinations of chords, themselves weighted combinations of the fifth-ordered pitches of Eq (S1) (Appendix S1); because the fifth index advances linearly in the z coordinate, each of the 24 pitch-class keys *recurs* every octave, and the implementation enumerates the nearby helical copies. We turn distances into probabilities with the maximum-entropy (Boltzmann/softmax) map of Eq (**??**), evaluated over the N nearest copies $\{d_{(1)} \leq \cdots \leq d_{(N)}\}$,

$$p(T_{(r)}) = \frac{e^{-\lambda d_{(r)}}}{\sum_{s=1}^{N} e^{-\lambda d_{(s)}}}, \qquad H = -\sum_{r=1}^{N} p(T_{(r)}) \log_2 p(T_{(r)}), \tag{S34}$$

with $N = 12$ and the convention $0 \log_2 0 = 0$. Fig S22 shows how a fixed distance vector is mapped to a near-uniform, graded, or spiked distribution as λ increases: the distances act as energies and λ as an inverse temperature.

The 12-key local neighborhood

The $N = 12$ nearest copies are the 12 distinct pitch-class keys closest to $\vec{c}_e$: across the corpus they are 12 distinct keys in 99.95% of bars (mean 11.999). The sorted distance profile rises smoothly past this local cluster (corpus-mean $d_{(12)} = 1.29$, $d_{(13)} = 1.40$, $d_{(22)} = 2.33$; Fig S23), so the remote and next-octave keys carry negligible mass at the calibrated λ. The entropy (S34) is therefore insensitive to the cutoff N: at $\lambda = 12$, computing H over the nearest 10 or 22 keys instead of 12 leaves the per-bar values rank-identical (Spearman $\rho = 1.00000$) with mean $|\Delta H| \leq 2 \times 10^{-3}$ bits. This is why restricting Eq (**??**) to the local neighborhood, rather than all 24 keys, does not change the reported uncertainty.

Geometric calibration of λ

We fix λ by the condition stated in the main text: when $\vec{c}_e$ sits on a key, $d_{(1)} = 0$ and that key must receive probability p_0. The remaining distances are then the fixed key-to-key spacings $\{\delta_j\}_{j \geq 2}$ of the spiral array, and (S34) gives

$$p_0 = \frac{1}{1 + \sum_{j \geq 2} e^{-\lambda \delta_j}} \quad \Longleftrightarrow \quad \sum_{j \geq 2} e^{-\lambda \delta_j} = \frac{1}{p_0} - 1. \tag{S35}$$

The left-hand side decreases monotonically in λ, so (S35) has a unique solution, obtained by bisection on the 24 central key copies (those nearest the corpus median z, to avoid helix-edge effects). The threshold pins λ to a narrow band: $p_0 = 0.95, 0.98, 0.99$ give $\lambda \approx 8.2$, 10.3, 12.0 respectively, while the adopted $\lambda = 12$ yields $p_0 = 0.9904$, comfortably satisfying $p_0 > 0.98$. Thus λ is a geometric constant of the representation rather than a tuned hyperparameter.

Bounds on the uncertainty

Entropy (S34) is bounded by $0 \leq H \leq \log_2 N = \log_2 12 \approx 3.585$ bits. The floor is the entropy of a $p_0 \approx 0.99$ distribution ($H \approx 0.08$ bits, $\vec{c}_e$ on a key). A *structured* maximum—$\vec{c}_e$ at the centroid of a key's 12 nearest neighbors—gives $H \approx 2.48$ bits, well below the uniform bound, because those 12 keys are not equidistant. Writing the neighborhood spread $s = d_{(12)} - d_{(1)}$, high uncertainty requires small s: the 50 most ambiguous bars have median $s = 0.27$ versus 0.89 across the corpus. Only 0.018% of bars exceed 2.5 bits, and the few that reach $\log_2 12$ exactly are degenerate measures with a non-finite center of effect (empty or zero-weight bars, mapped to the uniform fallback) or maximally compressed neighborhoods.

Robustness of the reported quantities

Re-evaluating (S34) on a grid of λ and aggregating per piece (mean over bars), we compare each λ against the calibrated $\lambda = 12$ on three structures: the per-piece ranking (Spearman ρ), the 20-year sliding-window median time series (Pearson r), and the uncertainty–year trend (Spearman ρ with year). Table S3 shows that the ranking and the temporal curve are highly stable across $\lambda \in [8, 24]$ and degrade only at the extremes, while the historical trend stays positive at every λ (no sign reversal). The absolute level of uncertainty rescales with λ, but the shape of the long-term trend—the Baroque maximum, the Classical decline, and the Romantic/Modern rise—is preserved (Fig S25). The historical features we report are therefore properties of the corpus and the spiral-array representation, not artefacts of λ; the bounded response and the cutoff invariance are summarized in Fig S24.

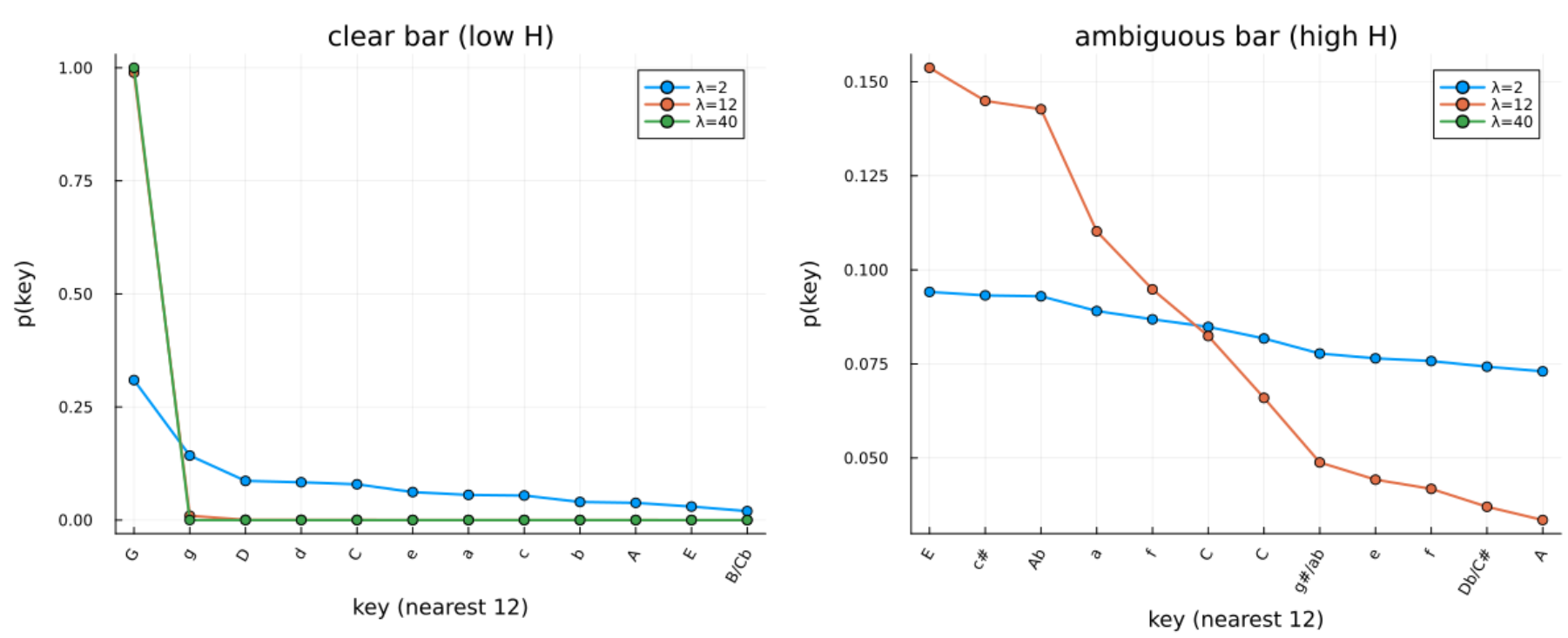

Fig S22. The Boltzmann distribution over the 12 nearest keys as a function of λ. For a tonally clear bar (left) and an ambiguous bar (right), the same distance vector is mapped to a near-uniform distribution at small λ, a graded distribution at the calibrated $\lambda = 12$, and a spike on the nearest key at large λ.

References

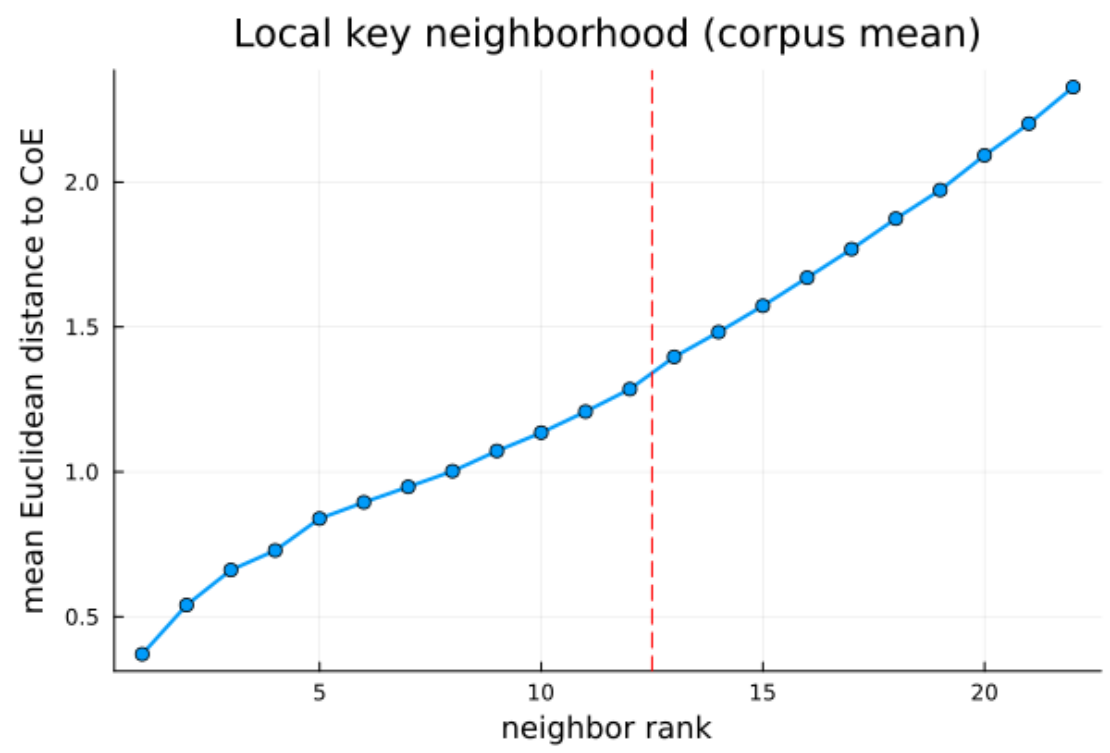

Fig S23. The local key neighborhood. Corpus-mean Euclidean distance from the center of effect to its r-th nearest key copy. The 12 nearest copies (left of the dashed line) form the local tonal neighborhood used by Eq (S34); distance rises smoothly beyond it.

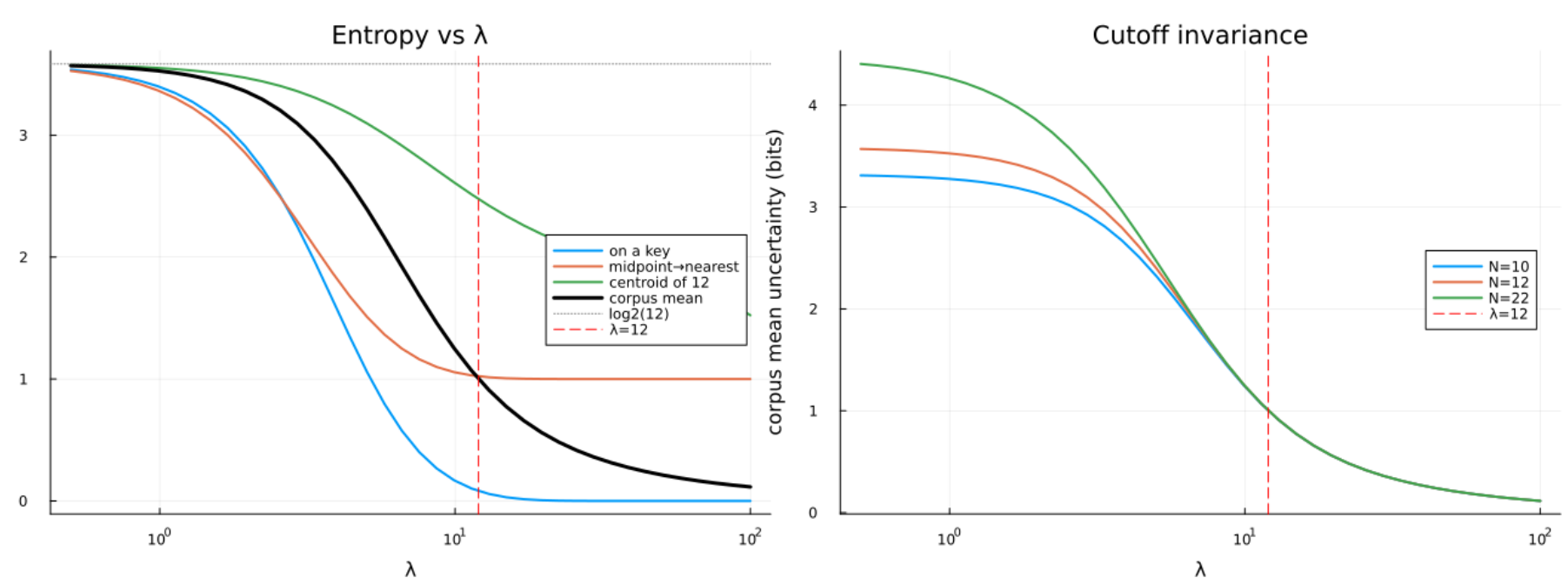

Fig S24. Entropy response to λ and cutoff invariance. Left: key uncertainty versus λ for three geometric reference points (center of effect on a key, at the midpoint to the nearest key, and at the centroid of the 12 nearest keys) and the corpus mean, with the uniform bound $\log_2 12$ (dotted) and the calibrated $\lambda = 12$ (dashed). Right: corpus-mean uncertainty versus λ computed over the nearest $N = 10, 12, 22$ keys; the curves coincide, confirming that the neighborhood cutoff is immaterial.

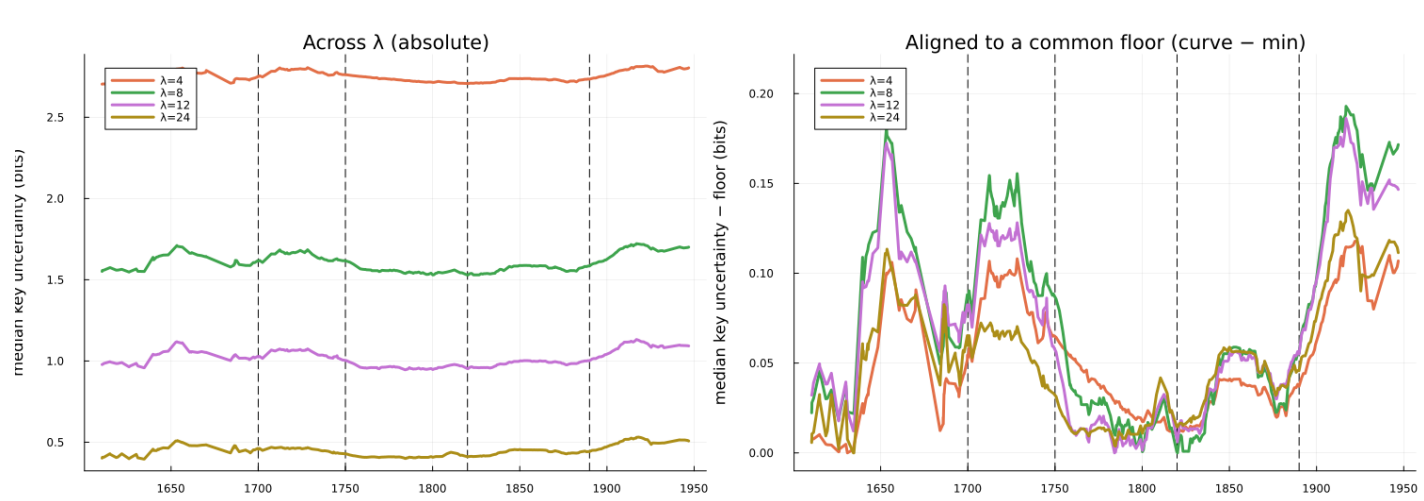

Fig S25. Key uncertainty over time is robust to λ. The 20-year sliding-window median of per-bar key uncertainty (same smoother as Fig **??**) for $\lambda \in \{4, 8, 12, 24\}$. Larger λ lowers the absolute level but preserves the shape of the long-term trend—the Baroque maximum, the Classical decline, and the Romantic/Modern rise. Dashed lines mark period boundaries.

Table S3. Robustness of key uncertainty to λ (reference $\lambda = 12$). Per-piece rank correlation, 20-year time-series correlation, and the uncertainty–year trend, over the full corpus.

λ	ρ(per-piece)	r(time series)	ρ(trend, year)
2	0.596	0.746	0.052
4	0.772	0.875	0.030
8	0.963	0.985	0.030
12	1.000	1.000	0.056
24	0.925	0.953	0.109
48	0.787	0.912	0.124
