# Supplementary material for: Quantifying the evolution of harmony and novelty in western classical music
Source: PLoS One. 2026 Aug 20;21(8):e0356548. doi: 10.1371/journal.pone.0356548 (PMC13492798; doi:10.1371/journal.pone.0356548)
Supplement: Supplementary file 10 [file pone.0356548.s010.pdf]

S10 Dataset and data processing

We curated a selection of MIDI files from the Kunst der Fugue website [1]. With the constraint of retaining only those pieces to which we could assign a year of composition, reducing the dataset to 4,638 MIDI files. We processed the pieces using a Julia script with the package `MusicSpiralRepresentation.jl` to extract the information needed to compute the center of effect: measure numbers, measure type, start quarter, end quarter, duration and pitch (in MIDI code), creating a data frame for a given piece with the columns given in Table S4.

The dataset used in this study can be found in [2], and the code to reproduce the computations and the figures of the paper is available in the repository cited above.

Other dataset statistics

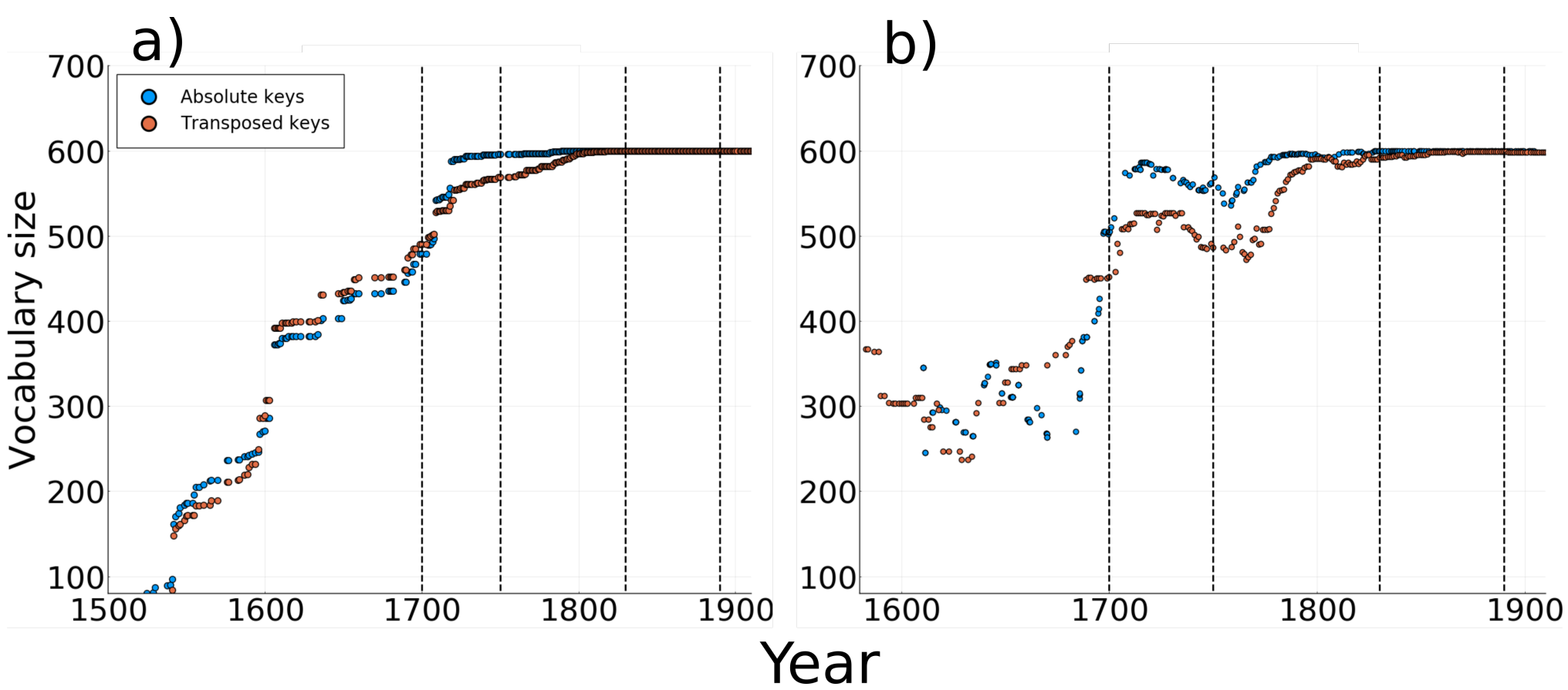

Fig S26. Vocabulary size. a) Cumulative vocabulary size. **b)** Windowed vocabulary size.

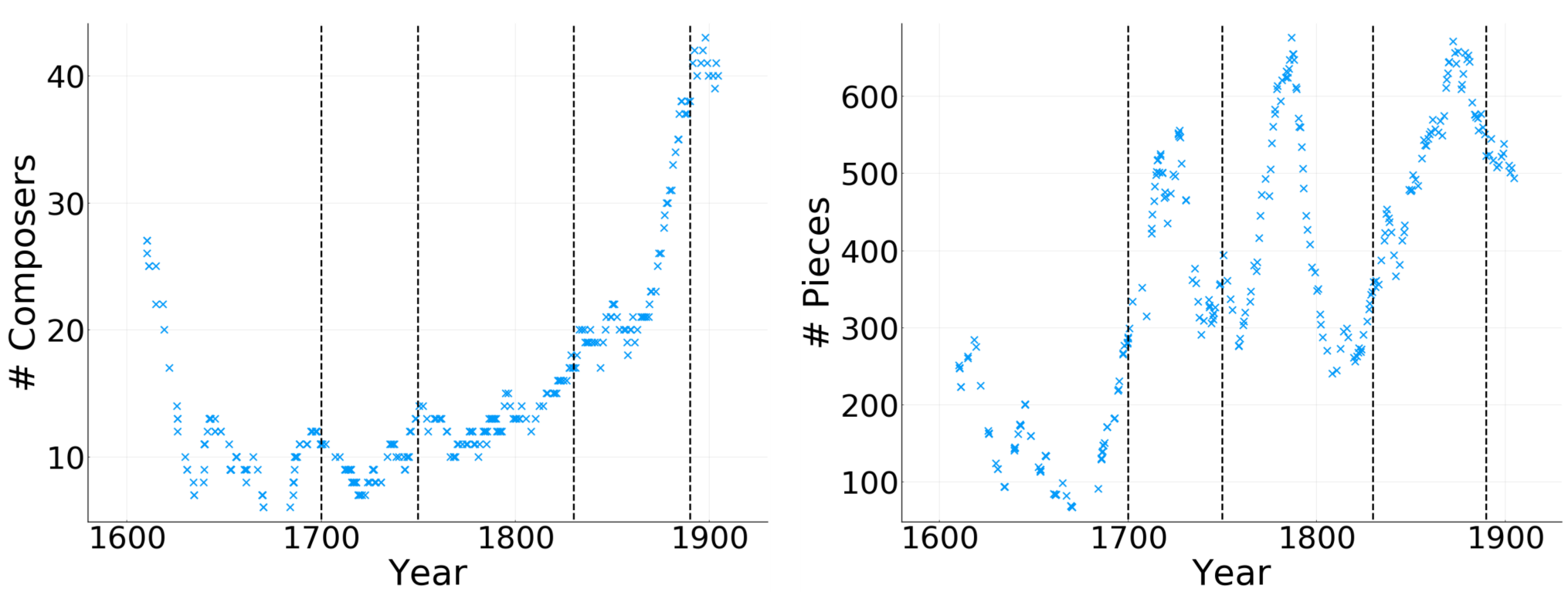

Fig S27. Left. Number of different composers over time. **Right.** Number of pieces over the years.

Table S4. Data frame structure for a musical piece.

measure_number	measure	start_quarter	end_quarter	duration	pitch

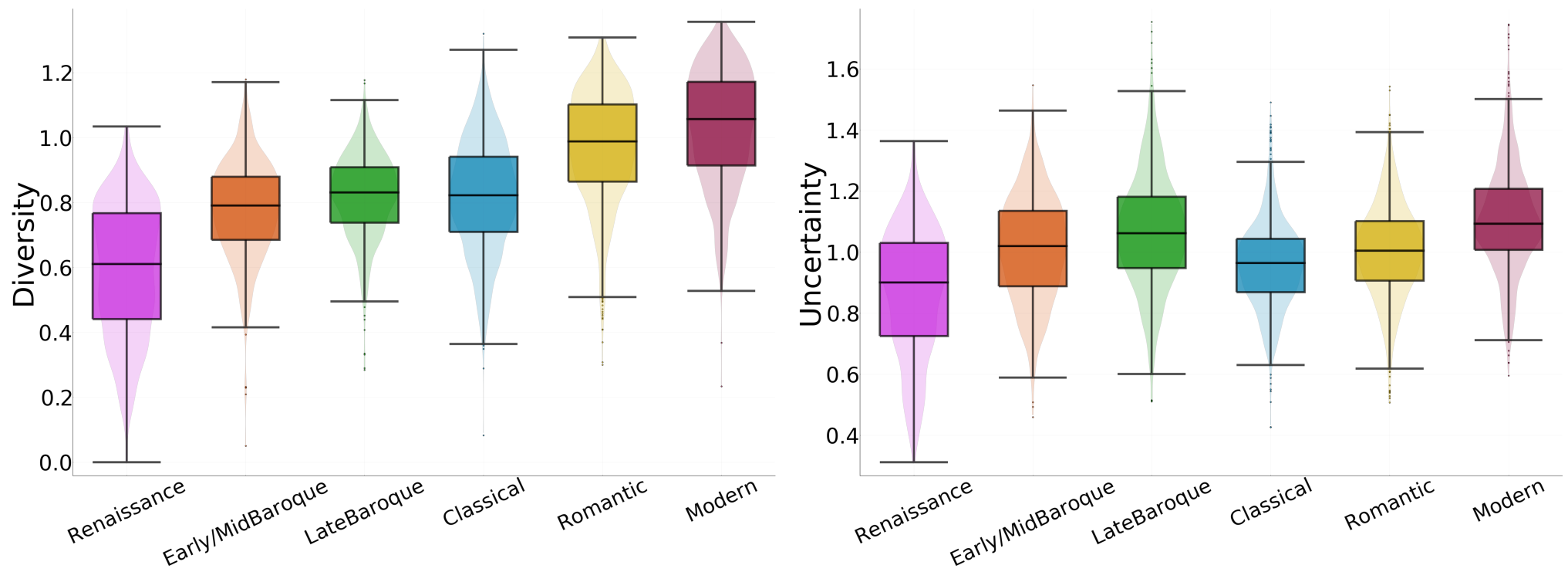

Fig S28. Diversity and uncertainty distributions on each historical period. Global trends from Figs **??** and **??** remain similar.

References

1. Authors V. Kunst Der Fugue, The largest resouce of classical music in .mid files; 2008. `https://www.kunstderfuge.com/`.

2. González-Espinoza A, Plotkin JB. Data for the paper "Quantifying the evolution of harmony and novelty in western classical music".; 2026. https://doi.org/10.1184/R1/31714561.

Table S5. Compositions list with YouTube links. List of the pieces highlighted in Fig 4, the number in the first column corresponds to the label in Fig 4. If links no longer work, readers are encouraged to search for other versions on YouTube or any streaming platform.

	**Year**	**Composer**	**Name**	**YT Link**
1	1911	Scriabin	Étude Op. 65, No. 1 (Étude in Ninths)	[1]
2	1915	Debussy	Étude No. 6: Pour les huit doigts	[2]
3	1915	Debussy	Étude No. 7: Pour les degrés chromatiques	[3]
4	1848	Alkan	12 Études in All the Major Keys, Op. 35, No. 11	[4]
5	1860	Liszt	Pater Noster (S.29)	[5]
6	1880	Dvořák	Waltz No. 6 in F major, Op. 54	[6]
7	1868	Raff	30 Progressive Etudes, WoO. 36, No. 1	[7]
8	1741	J.S. Bach	Goldberg Variations, BWV 988: Variation 23	[8]
9	1741	J.S. Bach	Goldberg Variations, BWV 988: Variation 30	[9]
10	1869	Hymns	The Great Physician Now Is Near	[10]
11	1825	Hymns	Brethren, We Have Met to Worship (tune: Holy Manna)	[11]
12	1735	Hymns	Jesus Makes My Heart Rejoice (tune: Herrnhut)	[12]

YouTube URLs:

1. https://youtu.be/qEar8zYFEJA?si=IzEWfhIosXOgIljW
2. https://youtu.be/5m1j6FOXCjs?si=PvaIsr4wmd43mRQv
3. https://youtu.be/YiKv3ASuh7U?si=KacyayZq-efdfgfL
4. https://youtu.be/W0NiP4NAYAo?si=Nj-e_upZUXqHhOJE
5. https://youtu.be/a9HDaeMx_WA?si=Qv-eR1pw3cpl4Xz9
6. https://youtu.be/bSLc8ZAndhM?si=JyH4ViYhhomRX3kS
7. https://youtu.be/pa1AUJVacFQ?si=ahZNzej6VjIHiKTG
8. https://youtu.be/4uE9fu0FaPg?si=LpjGudQCcu6bHaCV
9. https://youtu.be/bsdPChenTd0?si=or8v0AxDg139BfEW
10. https://youtu.be/T7If5BXR3Gw?si=ylzJMxNP22biKyxG
11. https://youtu.be/5T_ajnXGdpo?si=Ha0hos_EsPZL6zkt
12. https://youtu.be/vyrSHK3-pVE?si=tuPJBMFaQqMj5My6
