# Supplementary material for: Quantifying the evolution of harmony and novelty in western classical music
Source: PLoS One. 2026 Aug 20;21(8):e0356548. doi: 10.1371/journal.pone.0356548 (PMC13492798; doi:10.1371/journal.pone.0356548)
Supplement: Supplementary file 11 [file pone.0356548.s011.pdf]

Quantifying the evolution of harmony and novelty in Western classical music

General remarks

- I quite like your paper and believe that it is a great contribution to the quantitative study of harmony. I have added a number of specific comments to your sections below.
- My biggest general remark is that in the version we reviewers got the Figures had a VERY LOW resolution and were hard to read. For guessing the general trends it was fine, but make sure that you upload high-resolution figures for final publication!
- I am also glad to see that you make all your code open access! Thank you!

Specific comments

Abstract

- Maybe use "bar" for the musical unit, as it could be confused with "measures" as a metric. ("...measure by measure. We further develop measures...")

Introduction

- L 29: It is not quite adequate to describe R. Gjerdingen as a psychologist - although he has written about music perception, but from his perspective as a music theorist. There are plenty of prominent authors to cite here. The first ones that come to mind are Carol Krumhansl, Ian Cross, Tuomas Eerola, Lisa Margulis. You should have no problem in finding suitable references here.

Materials and methods

Key representation in the spiral array

- Fig. 1: Your labeling system is not quite standard. I assume you indicate the local keys of C and F major simply with "C" and "F", and chords within this local key as "G/C" for instance. As this notation looks too closely to lead-sheet conventions (where "G/C" would mean "G major chord with C in the bass), you should briefly explain it in the main text (around line 61) and caption of Figure 1.
- L 75: You should specify here whether you assume enharmonic equivalence (otherwise the spiral would be a torus) and octave equivalence. In fact, the spiral array conforms now to a 3D-embedding of the line of fifths, see [1, 3]. You mention this further below, L. 96, that you do indeed assume enharmonic equivalence (a necessity drawn from the encoding of your data, not on theoretical grounds), but it should already mentioned here.
- L 80: I suggest you use lowercase $\vec{p}$ also in Eq. 1. This way there is no confusion between sets and individual notes, although you also use p for probabilities later....
- Eq 2: The symbol for the center of effect $\vec{C}_e$ is very close to that of major and minor chords, which could be confusing. Maybe use E or another letter? Also, in lines 86 and following, you use a lowercase c. For the local keys, the T's are now boldfaced, which was not the case in line 77. Although it is perfectly clear what you are doing (especially when one knows the spiral array), you should ensure that your formalism is consistent throughout the paper and the appendix!
- L. 104: What is the reference for the Mozart sonatas? Is it [2]?

Key diversity and uncertainty

- Stating how to normalize counts in Eq 4 is not really necessary, but you could mention that you define key diversity further below (I was wondering why you didn't give a formula first).
- Eq 5: It is not clear to me how λ is defined, your explanation only talks about the factors involving T_i.
- Regarding your operationalization of key uncertainty: Since you calculate it measure-wise, how is the number of chords per measure taken into account? If I understand it correctly, a measure with a higher number of chords (such as at cadential moments that usually have accelerated harmonic rhythm) will increase key uncertainty, although all chords might perfectly well express a certain chord (and key). Should you thus maybe normalize by the number of chords present in a measure?
- I understand that, for your analysis, you need a single number expressing key uncertainty, but the median is really reductive, especially since I would assume that the distribution of chords/keys and their uncertainties in most pieces is not normal or even unimodal, but rather a rather complex thing. This deserves a little discussion at least.

Innovation in key transitions

- L. 142: Remove the final period (".") from the Meyer quote, as the sentence continues.
- L. 149: The opening parenthesis is missing.
- L. 152: "seen as the outcome of a Markov chain" is slightly imprecise. ξ can be seen *as* a Markov chain, which is, in turn, the outcome of a Markov process.
- L. 161 and following: I like this account of innovation very much. It's simple but effective. Nice work! But wouldn't Mutual Information make more sense then KL divergence?
- Eq 8: One of the sums should iterate over $e_i \in V_\Omega$, not V_Ξ, and the other one should have a lower-case ξ as index, I believe, because it is piece-wise. Does μ_i need an index? As the entire formula is defined for a specific piece ξ, it should be the same for all rows of P, no? Maybe I'm missing something. Just make sure Eq. 8 is correct. The meaning is clear.
- L. 173ff: This is similar to the procedure in [1] and [4]. You might want to have a look.

Corpus

- I suggest to cite *Kunder der Fuge* simply in a footnote, not as a proper reference, as the "Various Authors" field entry looks weird in this citation style.
- I tried to run your notebooks (I am a Python person) and managed to get it to run, but it's a bit strange that the data is not contained in the GitHub repo. Could you add a little script so that one can easily pull the data from a different source so that the notebooks run smoothely? I've had some trouble with adding the requirements and especially PyCall (for music21). Could you maybe have a colleague test whether they can reproduce your code?

Results

Key diversity and uncertainty

- Figure 2: I suggest that you use Arabic numerals (1, 2, 3, etc.) for the historical periods, because you already use Roman numerals for the chords. Unfortunately, the resolution of all of your figures is so bad so that I can see neither the median line, nor the different quantiles. I only see a broad orange band, but that is enough to imagine the rest. Ensure that your final graphics are all produced in a high resolution! I've had a similar problem with PLOS ONE before myself.
- L. 230: It's a nice service to the readers to provide some YouTube links for listening. Especially useful for a general-purpose journal.
- I am missing an interpretation of the positive but weak correlation between diversity and uncertainty. I would think that this indicates that both measures are not complementary, but partly measure the same thing, tonal complexity, on different levels of granularity (measures vs pieces).

Innovation in key transitions

- L. 244: "actual key name" --> "absolute key". Maybe also list minor keys as examples.
- L. 249: "secular variation": I have no idea what this means in this context. Do you mean "temporal"? Because "secular" is usually the antonym to "religious"
- Fig. 5: If transposition to the tonic meant simply adding or subtracting a particular interval, the two curves should be translations of one another. This is not the case. Can you say why? I suspect that there is a "confounding" factor, namely that not only the local key transitions change over time, but also the usage of certain keys. So, in the earlier periods we see less difference between the two curves because the global tonics come from the same small vocabulary of keys, which increases over time as well, so that innovation is larger simply because new keys are used. In other words: if a 19-th century piece would be an exact copy of a Mozart sonata, but in the key of Abb-minor (yes, double-flat) than relative innovation would be quite high in absolute terms but not in relative ones (there would be no innovation at all). This should at least be mentioned explicitly. Also, I believe this is the first time I read about the 20-year window. Maybe mention this already for the earlier figures.
- L. 269: Regarding this little discussion around complexity: It is always tempting to say that the classical period is less complex than the romantic because of lower chromaticism. While this is probably true, your method does not really care about chromaticism, but about changes in the transition matrices (which, I assume, are based on an identical chord vocabulary?). You mention yourself above, that the Baroque was quite a chromatic period, which would mean that Mozart is innovative because he is NOT chromatic.
- L. 286: This is a really cool and interesting result about the minor keys!

Discussion

- This is a good discussion, mentioning both the benefits and challenges of your approach. As all our approaches are limited, your discussion helps to contextualize the meaning of your results and points towards points of action for further improvement. Thank you!

Suggested references

[1] Moss, F. C., Neuwirth, M., & Rohrmeier, M. (2022). The line of fifths and the co-evolution of tonal pitch-classes. *Journal of Mathematics and Music*, *17*(2), 173–197. https://doi.org/10.1080/17459737.2022.2044927

[2] Hentschel, J., Neuwirth, M., & Rohrmeier, M. (2021). The Annotated Mozart Sonatas: Score, Harmony, and Cadence. *Transactions of the International Society for Music Information Retrieval*, *4*(1). https://doi.org/10.5334/tismir.63

[3] Temperley, D. (2000). The Line of Fifths. *Music Analysis*, *19*(3), 289–319. https://doi.org/10.2307/854457

[4] Moss, F. C., & Nakamura, E. (2024). Modeling the evolution of harmony in popular music from different cultural contexts. In W. Haverals, M. Koolen, & R. D. Thompson (Eds.), *Proceedings of the Fifth Conference on Computational Humanities Research (CHR 2024)* (Vol. 3834, pp. 137–152). CEUR. https://ceur-ws.org/Vol-3834/paper133.pdf
