# Supplementary material for: Quantifying the evolution of harmony and novelty in western classical music
Source: PLoS One. 2026 Aug 20;21(8):e0356548. doi: 10.1371/journal.pone.0356548 (PMC13492798; doi:10.1371/journal.pone.0356548)
Supplement: Supplementary file 12 [file pone.0356548.s013.pdf]

REVIEWER 2

We thank the referee for the careful reading of our manuscript and for the constructive comments, which we address point by point below.

General remarks

I quite like your paper and believe that it is a great contribution to the quantitative study of harmony. I have added a number of specific comments to your sections below.

Thank you. We are currently working on projects that extend this approach.

My biggest general remark is that in the version we reviewers got the Figures had a VERY LOW resolution and were hard to read. For guessing the general trends it was fine, but make sure that you upload high-resolution figures for final publication!

This is an artifact of the PLOS compilation process; we have seen it in other reviews. Clicking a figure in the PLOS-generated PDF opens the full-resolution version in a separate window. For final publication we supply all figures as high-resolution files.

I am also glad to see that you make all your code open access! Thank you!

Specific comments

Abstract

Maybe use "bar" for the musical unit, as it could be confused with "measures" as a metric ("...measure by measure. We further develop measures...").

We agree, and have replaced "measure" with "bar" throughout the manuscript.

Introduction

L 29: It is not quite adequate to describe R. Gjerdingen as a psychologist - although he has written about music perception, but from his perspective as a music theorist. There are plenty of prominent authors to cite here. The first ones that come to mind are Carol Krumhansl, lan Cross, Tuomas Eerola, Lisa Margulis. You should have no problem in finding suitable references here.

The referee is right. We have corrected the description and added appropriate references.

Materials and methods

Key representation in the spiral array Fig. 1: Your labeling system is not quite standard. I assume you indicate the local keys of C and F major simply with "C" and "F", and chords within this local key as "G/C" for instance. As this notation looks too closely to lead-sheet conventions (where "G/C" would mean "G major chord with C in the bass), you should briefly explain it in the main text (around line 61) and caption of Figure 1.

Good point. We have removed the notation from the figure and now explain it in the text and the Figure 1 caption, to avoid confusion with lead-sheet conventions.

L 75: You should specify here whether you assume enharmonic equivalence (otherwise the spiral would be a torus) and octave equivalence. In fact, the spiral array conforms now to a 3D-embedding of the line of fifths, see [1, 3]. You mention this further below, L. 96, that you do indeed assume enharmonic equivalence (a necessity drawn from the encoding of your data, not on theoretical grounds), but it should already mentioned here.

We have added the enharmonic-equivalence assumption where the spiral array is first introduced, and we note there that it follows from our data encoding rather than from theory.

L 80: I suggest you use lowercase p also in Eq. 1. This way there is no confusion between sets and individual notes, although you also use p for probabilities later...

Addressed as part of the notation revision described in our reply to the next comment.

Eq 2: The symbol for the center of effect $\vec{C}_e$ vector is very close to that of major and minor chords, which could be confusing. Maybe use E or another letter? Also, in lines 86 and following, you use a lowercase c. For the local keys, the T's are now boldfaced, which was not the case in line 77. Although it is perfectly clear what you are doing (especially when one knows the spiral array), you should ensure that your formalism is consistent throughout the paper and the appendix!

Point taken. We have changed the notation to match E. Chew's original paper and made it consistent throughout the manuscript and appendix.

L. 104: What is the reference for the Mozart sonatas? Is it [2]?

Yes; we have added this reference. Thank you.

Key diversity and uncertainty Stating how to normalize counts in Eq 4 is not really necessary, but you could mention that you define key diversity further below (I was wondering why you didn't give a formula first).

We now point forward to the definition of key diversity where Eq. 4 is introduced.

Eq 5: It is not clear to me how is defined, your explanation only talks about the factors involving T_i.

This definition was underspecified in the original. We have added an explanation of both entropies, with more detail on the definition and the intuition behind it.

Regarding your operationalization of key uncertainty: Since you calculate it measure-wise, how is the number of chords per measure taken into account? If I understand it correctly, a measure with a higher number of chords (such as at cadential moments that usually have accelerated harmonic rhythm) will increase key uncertainty, although all chords might perfectly well express a certain chord (and key). Should you thus maybe normalize by the number of chords present in a measure?

This is a fair point. The window we use to compute the center of effect can contain more information than can be reliably attributed to one key, and the referee's reading is correct: competing chords that pull the center in different directions raise the uncertainty about a single key. Normalizing by the number of chords per bar, or by window, is a reasonable alternative (we already normalize by note duration in the center-of-effect computation), but it is outside the scope of this study. We chose a fixed, time-based unit that is musically meaningful, the bar. We note in the text that varying the window or the normalization is a promising direction for further work.

I understand that, for your analysis, you need a single number expressing key uncertainty, but the median is really reductive, especially since I would assume that the distribution of chords/keys and their uncertainties in most pieces is not normal or even unimodal, but rather a rather complex thing. This deserves a little discussion at least.

We share this reservation; in our experience the median is not the best single summary of this feature. We are developing a more dynamic representation that tracks key uncertainty at higher resolution, which we expect to capture this "uncertainty profile" more appropriately. We have added text making this point and noting the need for studies that address it.

Innovation in key transitions L. 142: Remove the final period (".") from the Meyer quote, as the sentence continues.

Removed.

L. 149: The opening parenthesis is missing.

Fixed.

L. 152: "seen as the outcome of a Markov chain" is slightly imprecise. It can be seen as a Markov chain, which is, in turn, the outcome of a Markov process.

We have reworded the sentence: the key sequence can be seen as a Markov chain, which is itself the outcome of a Markov process.

L. 161 and following: I like this account of innovation very much. It's simple but effective. Nice work! But wouldn't Mutual Information make more sense then KL divergence?

Thank you. Mutual information measures statistical dependence and is normally used for the dependence within a single sequence, not for comparing two different sequences. Prompted by this question, we added a new section that uses the Jensen–Shannon divergence (JSD) to compare key transitions across composers; the results give further insight into the measures we develop. We also added a paragraph justifying our use of the KL divergence.

Eq 8: One of the sums should iterate over $e_i \in V_\Omega$, not V_Ξ and the other one should have a lower-case e as index, I believe, because it is piece-wise. Does μ_i need an index? As the entire formula is defined for a specific piece, it should be the same for all rows of P, no? Maybe I'm missing something. Just make sure Eq. 8 is correct. The meaning is clear.

The equation is correct as written. It is the KL-divergence *rate* between two Markov chains, not an ordinary KL divergence, which is why μ_i carries an index: μ_i is the i-th entry of the stationary distribution of the piece's transition matrix P (a vector over the 24 keys), and the outer sum is the stationary-weighted average of each row's divergence, the relative-entropy analogue of the entropy rate $H_R(P) = \sum_i \mu_i H_i$. This is also why we find no strong relationship between the KLD rate and the number of key transitions. We have made the index sets and the summation explicit in the revised text.

L. 173ff: This is similar to the procedure in [1] and [4]. You might want to have a look.

These two studies address the evolution of harmony but measure different objects, which is why we treat them separately. [4] infers population-level chord-substitution probabilities under a cultural-transmission model, and [1] describes tonal organization through the static, undirected co-occurrence of pitch classes along the line of fifths. Neither gives a directed, per-piece, per-step measure of how far a score's key-transition dynamics depart from its accumulated past, which is what our KL-divergence rate captures. We now cite both in the Methods and Discussion, with a comparison.

Corpus I suggest to cite Kunder der Fuge simply in a footnote, not as a proper reference, as the "Various Authors" field entry looks weird in this citation style.

We were not able to implement this edit because PLoS guidelines don't allow for the use of footnotes, and they suggest including links as references.

I tried to run your notebooks (I am a Python person) and managed to get it to run, but it's a bit strange that the data is not contained in the GitHub repo. Could you add a little script so that one can easily pull the data from a different source so that the notebooks run smoothly? I've had some trouble with adding the requirements and especially PyCall (for music21). Could you maybe have a colleague test whether they can reproduce your code?

We have removed the PyCall dependency and added a script that pulls the data and runs the code with minimal requirements. We also checked that the notebooks run from a clean install.

Results

Key diversity and uncertainty

Figure 2: I suggest that you use Arabic numerals (1, 2, 3, etc.) for the historical periods, because you already use Roman numerals for the chords. Unfortunately, the resolution of all of your figures is so bad so that I can see neither the median line, nor the different quantiles. I only see a broad orange band, but that is enough to imagine the rest. Ensure that your final graphics are all produced in a high resolution! I've had a similar problem with PLOS ONE before myself.

Done. We now use Arabic numerals for the historical periods and keep Roman numerals for the chords. On resolution, see our reply above: clicking the figure in the PLOS PDF opens the full-resolution version, and the final files are high-resolution.

L. 230: It's a nice service to the readers to provide some YouTube links for listening. Especially useful for a general-purpose journal.

Thank you. We agree that hearing the pieces conveys what our measures capture, and we have kept these links.

I am missing an interpretation of the positive but weak correlation between diversity and uncertainty. I would think that this indicates that both measures are not complementary, but partly measure the same thing, tonal complexity, on different levels of granularity (measures vs pieces).

We agree. The weak positive correlation probably reflects some shared structure, since both measures relate to tonal complexity at different scales (per bar for uncertainty, per piece for diversity). Part of it may also be noise: a high-uncertainty bar is one where the algorithm's most likely key is not the intended one, which adds noise to the key sequence and can carry into the diversity measure. Our framework cannot separate the shared tonal structure from this algorithmic noise, so we do not push the question further here, but we agree it is worth pursuing. We have added a sentence to the manuscript noting this.

Innovation in key transitions

L. 244: "actual key name" –¿ "absolute key". Maybe also list minor keys as examples.

Done. We also added minor-key examples.

L. 249: "secular variation": I have no idea what this means in this context. Do you mean "temporal"? Because "secular" is usually the antonym to "religious".

The referee is right that the term is confusing. Although "secular" is standard in time-series analysis, it is not suited to this audience, and we have replaced it with "long-term."

Fig. 5: If transposition to the tonic meant simply adding or subtracting a particular interval, the two curves should be translations of one another. This is not the case. Can you say why? I suspect that there is a "confounding" factor, namely that not only the local key transitions change over time, but also the usage of certain keys. So, in the earlier periods we see less difference between the two curves because the global tonics come from the same small vocabulary of keys, which increases over time as well, so that innovation is larger simply because new keys are used. In other words: if a 19-th century piece would be an exact copy of a Mozart sonata, but in the key of Abb-minor (yes, double-flat) than relative innovation would be quite high in absolute terms but not in relative ones (there would be no innovation at all). This should at least be mentioned explicitly. Also, I believe this is the first time I read about the 20-year window. Maybe mention this already for the earlier figures.

Thank you; we were also puzzled that the curves were not translations of one another. After filtering the pieces and recomputing the novelties (and fixing a bug we found in the process, thank you for prompting this), the two curves are now translations of one another. The confounding effect the referee describes may still be present, but it is not visible in the corrected result. We also now introduce the 20-year window at its first use, so it appears with the earlier figures.

L. 269: Regarding this little discussion around complexity: It is always tempting to say that the classical period is less complex than the romantic because of lower chromaticism. While this is probably true, your method does not really care about chromaticism, but about changes in the transition matrices (which, I assume, are based on an identical chord vocabulary?). You mention yourself above, that the Baroque was quite a chromatic period, which would mean that Mozart is innovative because he is NOT chromatic.

We have revised the wording: we no longer call this "complexity" and instead refer to "tonal ambiguity," which is less easily misread. The referee's intuition is compatible with our framework. Chromatic writing should in principle raise the uncertainty in the center-of-effect computation, but that is not exactly what we measure, and it enters as noise in the innovation value in the way we described in our reply on the

diversity–uncertainty correlation. We do not quantify how much it contributes to innovation. One plausible reading is that, because harmony is more clearly defined in the Classical period, the center-of-effect algorithm more readily finds a low-uncertainty key; the innovation would then be driven by whatever key-transition conventions were common in that period. We think innovation reflects a combination of modulation, tonal ambiguity, and repetition within the key sequence, and it is hard to attribute to any single factor.

L. 286: This is a really cool and interesting result about the minor keys!

Thank you. Because of the new section, we have moved this plot to the Supplement to keep the main text readable, but we kept the paragraph that discusses it so readers can find it.

Discussion

This is a good discussion, mentioning both the benefits and challenges of your approach. As all our approaches are limited, your discussion helps to contextualize the meaning of your results and points towards points of action for further improvement. Thank you!

Thank you.

Suggested references

[1] Moss, F. C., Neuwirth, M., & Rohrmeier, M. (2022). The line of fifths and the co-evolution of tonal pitch-classes. Journal of Mathematics and Music, 17(2), 173-197. `https://doi.org/10.1080/17459737.2022.2044927` [2] Hentschel, J., Neuwirth, M., & Rohrmeier, M. (2021). The Annotated Mozart Sonatas: Score, Harmony, and Cadence. Transactions of the International Society for Music Information Retrieval, 4(1). `https://doi.org/10.5334/tismir.63` [3] Temperley, D. (2000). The Line of Fifths. Music Analysis, 19(3), 289-319. `https://doi.org/10.2307/854457` [4] Moss, F. C., & Nakamura, E. (2024). Modeling the evolution of harmony in popular music from different cultural contexts. In W. Haverals, M. Koolen, & R. D. Thompson (Eds.), Proceedings of the Fifth Conference on Computational Humanities Research (CHR 2024) (Vol. 3834, pp. 137-152). CEUR. `https://ceur-ws.org/Vol-3834/paper133.pdf`
