# Supplementary material for: Quantifying the evolution of harmony and novelty in western classical music
Source: PLoS One. 2026 Aug 20;21(8):e0356548. doi: 10.1371/journal.pone.0356548 (PMC13492798; doi:10.1371/journal.pone.0356548)
Supplement: Peer review history

# Peer review: Decision Letter 0

**DOI:** 10.1371/journal.pone.0356548.r001

**Authors:** Bruno Mesz

22 May 2026

PONE-D-26-14871

Quantifying the evolution of harmony and novelty in Western classical music

PLOS One

Dear Dr. González-Espinoza,

Thank you for submitting your manuscript to PLOS ONE. After careful consideration, we feel that it has merit but does not fully meet PLOS ONE’s publication criteria as it currently stands. Therefore, we invite you to submit a revised version of the manuscript that addresses the points raised during the review process.

Your submission has been evaluated by three expert referees in computational musicology and statistical style analysis, and their detailed comments are appended below<source-footnote _nghost-ng-c2549839294="" ng-version="0.0.0-PLACEHOLDER"></source-footnote>. While your application of information-theoretic metrics to a geometric model of tonality across a massive historical corpus is "a great contribution to the qunatitative study of harmony", as Reviewer 2 says, the manuscript currently suffers from critical conceptual ambiguities, parameter vulnerabilities, and a structural layout error in the supplementary graphics that must be addressed before publication.

In addtion to the comments from the reviewers, both Reviewer 3 and I are concerned by the structural flattening caused by your reliance on a first-order Markov chain proxy<source-footnote _nghost-ng-c2549839294="" ng-version="0.0.0-PLACEHOLDER"></source-footnote><source-footnote _nghost-ng-c2549839294="" ng-version="0.0.0-PLACEHOLDER"></source-footnote><source-footnote _nghost-ng-c2549839294="" ng-version="0.0.0-PLACEHOLDER"></source-footnote><source-footnote _nghost-ng-c2549839294="" ng-version="0.0.0-PLACEHOLDER"></source-footnote>. I ask you to implement a formal Markov order control test, such as the Akaike Information Criterion or Bayesian Information Criterion, across a small, representative subset of landmark files from your corpus (for example, selecting 20 prominent works each from J.S. Bach, Mozart, Wagner, and Stravinsky).  If this test reveals that the optimal Markovian memory depth shifts systematically across history it would be an interesting finding meriting discussion in terms of musical innovation.

Another issue is the omission of an specific value of the smoothing parameter beta in the computation of KL divergence. Moreover, with the expansion of vocabulary along history, this parameter can introduce distorsions. You must run a sensitivity analysis demonstrating that your historical novelty peaks are robust across disparate values of beta or use a data-driven smoothing algorithm.

Please check the mismatch between your Supporting Information caption inventory in the text (Pages 20–21) and the figures.

Please submit your revised manuscript by Jul 06 2026 11:59PM. If you will need more time than this to complete your revisions, please reply to this message or contact the journal office at plosone@plos.org. When you're ready to submit your revision, log on to https://www.editorialmanager.com/pone/ and select the 'Submissions Needing Revision' folder to locate your manuscript file.

Please include the following items when submitting your revised manuscript:

- A letter that responds to each point raised by the academic editor and reviewer(s). You should upload this letter as a separate file labeled 'Response to Reviewers'.
- A marked-up copy of your manuscript that highlights changes made to the original version. You should upload this as a separate file labeled 'Revised Manuscript with Track Changes'.
- An unmarked version of your revised paper without tracked changes. You should upload this as a separate file labeled 'Manuscript'.

If you would like to make changes to your financial disclosure, please include your updated statement in your cover letter. Guidelines for resubmitting your figure files are available below the reviewer comments at the end of this letter.

If applicable, we recommend that you deposit your laboratory protocols in protocols.io to enhance the reproducibility of your results. Protocols.io assigns your protocol its own identifier (DOI) so that it can be cited independently in the future. For instructions see: https://journals.plos.org/plosone/s/submission-guidelines#loc-laboratory-protocols. Additionally, PLOS ONE offers an option for publishing peer-reviewed Lab Protocol articles, which describe protocols hosted on protocols.io. Read more information on sharing protocols at https://plos.org/protocols?utm_medium=editorial-email&utm_source=authorletters&utm_campaign=protocols.

As the corresponding author, your ORCID iD is verified in the submission system and will appear in the published article. PLOS supports the use of ORCID, and we encourage all coauthors to register for an ORCID iD and use it as well. Please encourage your coauthors to verify their ORCID iD within the submission system before final acceptance, as unverified ORCID iDs will not appear in the published article. *Only*  the individual author can complete the verification step; PLOS staff *cannot*  verify ORCID iDs on behalf of authors.

We look forward to receiving your revised manuscript.

Kind regards,

Bruno Alejandro Mesz, Ph.D.

Academic Editor

PLOS One

Journal Requirements:

When submitting your revision, we need you to address these additional requirements.

1. Please ensure that your manuscript meets PLOS ONE's style requirements, including those for file naming. The PLOS ONE style templates can be found at

https://journals.plos.org/plosone/s/file?id=wjVg/PLOSOne_formatting_sample_main_body.pdf and

https://journals.plos.org/plosone/s/file?id=ba62/PLOSOne_formatting_sample_title_authors_affiliations.pdf

2. Please note that PLOS One has specific guidelines on code sharing for submissions in which author-generated code underpins the findings in the manuscript. In these cases, we expect all author-generated code to be made available without restrictions upon publication of the work. Please review our guidelines at https://journals.plos.org/plosone/s/materials-and-software-sharing#loc-sharing-code and ensure that your code is shared in a way that follows best practice and facilitates reproducibility and reuse.

3. We notice that your supplementary figures are uploaded with the file type 'Figure'. Please amend the file type to 'Supporting Information'. Please ensure that each Supporting Information file has a legend listed in the manuscript after the references list.

4. We notice that your supplementary tables are included in the manuscript file. Please remove them and upload them with the file type 'Supporting Information'. Please ensure that each Supporting Information file has a legend listed in the manuscript after the references list.

5. If the reviewer comments include a recommendation to cite specific previously published works, please review and evaluate these publications to determine whether they are relevant and should be cited. There is no requirement to cite these works unless the editor has indicated otherwise.

[Note: HTML markup is below. Please do not edit.]

Reviewers' comments:

Reviewer's Responses to Questions

**Comments to the Author**

1. Is the manuscript technically sound, and do the data support the conclusions?

The manuscript must describe a technically sound piece of scientific research with data that supports the conclusions. Experiments must have been conducted rigorously, with appropriate controls, replication, and sample sizes. The conclusions must be drawn appropriately based on the data presented.

Reviewer #1: Yes

Reviewer #2: Yes

Reviewer #3: Partly

**********

2. Has the statistical analysis been performed appropriately and rigorously?

Reviewer #1: Yes

Reviewer #2: Yes

Reviewer #3: Yes

**********

3. Have the authors made all data underlying the findings in their manuscript fully available?

The PLOS Data policy requires authors to make all data underlying the findings described in their manuscript fully available without restriction, with rare exception (please refer to the Data Availability Statement in the manuscript PDF file). The data should be provided as part of the manuscript or its supporting information, or deposited to a public repository. For example, in addition to summary statistics, the data points behind means, medians and variance measures should be available. If there are restrictions on publicly sharing data—e.g. participant privacy or use of data from a third party—those must be specified.

Reviewer #1: Yes

Reviewer #2: Yes

Reviewer #3: Yes

**********

4. Is the manuscript presented in an intelligible fashion and written in standard English?

PLOS ONE does not copyedit accepted manuscripts, so the language in submitted articles must be clear, correct, and unambiguous. Any typographical or grammatical errors should be corrected at revision, so please note any specific errors here.

Reviewer #1: Yes

Reviewer #2: Yes

Reviewer #3: Yes

**********

5. Review Comments to the Author

Please use the space provided to explain your answers to the questions above. You may also include additional comments for the author, including concerns about dual publication, research ethics, or publication ethics. (Please upload your review as an attachment if it exceeds 20,000 characters)

Reviewer #1: This is a very comprehensive paper that combines a methodology rooted in modern techniques of musical analysis with rigorous statistical analysis and quantification. Although the results are somewhat to be expected, given the amount of studies devoted to this problem in the musicological and music theoretical literature, they reinforce the validity of the approach. The paper is well written and comprehensible also for not musically trained readers. If anything, the authors should make sure that the graphical quality of the images is at par with the journal requirements.

Reviewer #2: See attached PDF. See attached PDF. See attached PDF. See attached PDF. See attached PDF. See attached PDF. See attached PDF. See attached PDF. See attached PDF. See attached PDF. See attached PDF. See attached PDF.

Reviewer #3: The authors implement a variant of the center of effect algorithm to identify locally the key in a given piece of music. Then they proceed to use probabilistic, statistical and information theoretical methods to analyze harmonic features of the piece. They propose several measures to analyze what they call the harmonic complexity changes in a corpus spanning several centuries. These measures include the KL divergence to characterize the novelty of a piece.

Although there are interesting proposals, in my opinion there are also important conceptual misunderstandings, both musical and mathematical:

1. The most important one is the confusion of harmonic complexity with modulatory complexity. Their work focuses on the latter, as the CoE algorithm identifies local keys, that is, the modulatory structure of the piece, not the harmonic structure (that might be very complex, even if the whole piece remains in the same key). Therefore their assertions concerning comparisons of harmonic complexity should be revised.

2. Their comparison of different genres introduces biases in the analysis. There are genres intrinsically more harmonically complex than others. It would be preferable to work with a smaller corpus, but more consistent in this regard.

3. The use of Markov models, as the authors themselves acknowledge, oversimplifies the global structure of a work. In any case, their use might be justified in shorter genres, but in longer ones, I think that taking into account memory effects or long time correlations cannot be avoided.

On the other hand, the musical conclusions do not offer anything new. They confirm what has been known by traditional harmonic analysis and music history for a long time.

Additionally, the use of the KL divergence in quantifying stylistic differences between pieces is not new. in this respect, a more thorough revision of the literature would be advisable.

In summary, a careful revision, both terminological and conceptual, should be made in order to compare harmonic features and modulatory features consistently.

It would be interesting to explore a point briefly touched upon by the authors, namely, the connection between the textural features of a piece and its modulatory structure (a question that is interesting and that has not been addressed in the literature)

Having said this, I think that the paper contains interesting ideas and that and it could be published after incorporating the suggestions made above.

**********

6. PLOS authors have the option to publish the peer review history of their article (what does this mean?). If published, this will include your full peer review and any attached files.

If you choose “no”, your identity will remain anonymous but your review may still be made public.

**Do you want your identity to be public for this peer review?**  For information about this choice, including consent withdrawal, please see our Privacy Policy.

Reviewer #1: No

Reviewer #2: **Yes:** Fabian C. Moss

Reviewer #3: No

**********

[NOTE: If reviewer comments were submitted as an attachment file, they will be attached to this email and accessible via the submission site. Please log into your account, locate the manuscript record, and check for the action link "View Attachments". If this link does not appear, there are no attachment files.]

To ensure your figures meet our technical requirements, please review our figure guidelines: https://journals.plos.org/plosone/s/figures

You may also use PLOS’s free figure tool, NAAS, to help you prepare publication quality figures: https://journals.plos.org/plosone/s/figures#loc-tools-for-figure-preparation.

NAAS will assess whether your figures meet our technical requirements by comparing each figure against our figure specifications.

Attachment Submitted filename: Review - Quantifying the evolution of harmony.pdf

# Author comment: Author response to Decision Letter 1

**DOI:** 10.1371/journal.pone.0356548.r002

28 Jul 2026

We added an individual response (pdf) to each reviewer in this submission (see files submitted).

Attachment Submitted filename: Response_Reviewer_2.pdf

# Editor report: Decision Letter 1

**DOI:** 10.1371/journal.pone.0356548.r003

**Authors:** Bruno Mesz

4 Aug 2026

Quantifying the evolution of harmony and novelty in Western classical music

PONE-D-26-14871R1

Dear Dr. González-Espinoza,

We’re pleased to inform you that your manuscript has been judged scientifically suitable for publication and will be formally accepted for publication once it meets all outstanding technical requirements.

Within one week, you’ll receive an e-mail detailing the required amendments. When these have been addressed, you’ll receive a formal acceptance letter and your manuscript will be scheduled for publication.

An invoice will be generated when your article is formally accepted. Please note, if your institution has a publishing partnership with PLOS and your article meets the relevant criteria, all or part of your publication costs will be covered. Please make sure your user information is up-to-date by logging into Editorial Manager at Editorial Manager® and clicking the ‘Update My Information' link at the top of the page. For questions related to billing, please contact billing support.

If your institution or institutions have a press office, please notify them about your upcoming paper to help maximize its impact. If they’ll be preparing press materials, please inform our press team as soon as possible -- no later than 48 hours after receiving the formal acceptance. Your manuscript will remain under strict press embargo until 2 pm Eastern Time on the date of publication. For more information, please contact onepress@plos.org.

Kind regards,

Bruno Alejandro Mesz, Ph.D.

Academic Editor

PLOS One

Additional Editor Comments (optional):

Reviewers' comments:

# Editor report: Acceptance letter

**DOI:** 10.1371/journal.pone.0356548.r004

**Authors:** Bruno Mesz

PONE-D-26-14871R1

PLOS One

Dear Dr. González-Espinoza,

I'm pleased to inform you that your manuscript has been deemed suitable for publication in PLOS One. Congratulations! Your manuscript is now being handed over to our production team.

At this stage, our production department will prepare your paper for publication. This includes ensuring the following:

* All references, tables, and figures are properly cited

* All relevant supporting information is included in the manuscript submission,

* There are no issues that prevent the paper from being properly typeset

You will receive further instructions from the production team, including instructions on how to review your proof when it is ready. Please keep in mind that we are working through a large volume of accepted articles, so please give us a few days to review your paper and let you know the next and final steps.

Lastly, if your institution or institutions have a press office, please let them know about your upcoming paper now to help maximize its impact. If they'll be preparing press materials, please inform our press team within the next 48 hours. Your manuscript will remain under strict press embargo until 2 pm Eastern Time on the date of publication. For more information, please contact onepress@plos.org.

You will receive an invoice from PLOS for your publication fee after your manuscript has reached the completed accept phase. If you receive an email requesting payment before acceptance or for any other service, this may be a phishing scheme. Learn how to identify phishing emails and protect your accounts at https://explore.plos.org/phishing.

If we can help with anything else, please email us at customercare@plos.org.

Thank you for submitting your work to PLOS One and supporting open access.

Kind regards,

PLOS ONE Editorial Office Staff

on behalf of

Dr. Bruno Alejandro Mesz

Academic Editor

PLOS One
